# Elevated aggression is associated with uncertainty in a network of dog dominance interactions

**DOI:** 10.1098/rspb.2019.0536

**Published:** 2019-07-03

**Authors:** Matthew J. Silk, Michael A. Cant, Simona Cafazzo, Eugenia Natoli, Robbie A. McDonald

**Affiliations:** 1 Environment and Sustainability Institute, University of Exeter, Penryn TR10 9FE, Cornwall, UK; 2 Centre for Ecology and Conservation, University of Exeter, Penryn TR10 9FE, Cornwall, UK; 3 Wolf Science Center, Dörfles 48, 2115 Ernstbrunn, Austria; 4 Canile Sovrazonale, Servizio Veterinario, ASL Roma 3, Roma, Italy

**Keywords:** dominance hierarchy, social network, agonistic interaction, social stability, exponential random graph model

## Abstract

Dominance hierarchies are widespread in animal societies and reduce the costs of within-group conflict over resources and reproduction. Variation in stability across a social hierarchy may result in asymmetries in the benefits obtained from hierarchy formation. However, variation in the stability and behavioural costs of dominance interactions with rank remain poorly understood. Previous theoretical models have predicted that the intensity of dominance interactions and aggression should increase with rank, but these models typically assume high reproductive skew, and so their generality remains untested. Here we show in a pack of free-living dogs with a sex–age-graded hierarchy that the central region of the hierarchy was dominated by more unstable social relationships and associated with elevated aggression. Our results reveal unavoidable costs of ascending a dominance hierarchy, run contrary to theoretical predictions for the relationship between aggression and social rank in high-skew societies, and widen our understanding of how heterogeneous benefits of hierarchy formation arise in animal societies.

## 1. Introduction

Dominance hierarchies, in which high social rank confers priority of access to resources, are a feature of animal societies from insects to primates [1–4]. In many societies, dominant individuals are easily recognized because they engage in conspicuous displays or frequent acts of aggression towards other, subordinate group members [5,6]. In other societies, dominance is more difficult to infer because dominant individuals maintain their rank without resorting to obvious aggression [7–9], or because dominant individuals are not necessarily the most aggressive in the group [10].

Theoretical attempts to explain inter- and intraspecific variation in patterns of agonistic behaviour proceed by making an explicit assumption of the function of aggression, dominance or submission. The assumed function of agonistic interactions determines their predicted patterns within groups. For example, where aggressive interactions serve directly to outcompete or damage rivals, and submission signals a lack of motivation to challenge, one might predict most aggression (and perhaps most submission) where competitors are most unevenly matched. By contrast, if aggressive interactions primarily serve an information function, such as to advertise resource holding potential (RHP), to reveal the quality of opponents or, in the case of submissive behaviour, to conceal information about the motivation to challenge, one might predict most aggression and submission where the pay-off of winning is greatest and where competitors are most evenly matched [11,12].

While most models of dominance aggression assume a fixed hierarchy and examine the costs and benefits of aggression to individuals of different rank, patterns of aggression and submission may reflect instability or flux in social relationships within the group, or the clarity of the hierarchy to its members. Unstable regions of the hierarchy can be detected by there being fewer transitive relationships (A beats B, B beats C and A beats C) and more cyclical relationships (A beats B and B beats C, but C beats A) than would be expected, based on an overall network of hierarchical interactions [13]. Rank instability may be a costly but unavoidable feature of life in heterogeneous social groups in a dynamic social and ecological environment. Particular regions of social hierarchies may be more or less susceptible to rank instability, reducing or increasing the fitness pay-offs associated with given ranks.

To investigate both the function of agonistic behaviour and patterns of stability requires data on how patterns of aggression, dominance and submission behaviours vary within social hierarchies. Here, we use data on social interactions in free-living dogs *Canis familiaris* to test how dominance hierarchy stability varies with social rank and whether this carries behavioural costs to individuals within particular regions of the hierarchy. Free-living dogs frequently form multi-male, multi-female social groups consisting of both related and unrelated members [14,15]. While they behave cooperatively [16,17], they typically exhibit a promiscuous mating system [18], which would be expected to reduce reproductive skew. Free-living dogs have previously been reported to exhibit a linear dominance hierarchy [14,19–21], not dissimilar to that in wolves *Canis lupus* [22,23], in which older individuals are dominant over younger ones and males are dominant over females of similar age. However, unlike free-living dogs, wolves frequently live in closely related family groups, in which only the dominant pair reproduce [23]. Aggressive interactions in group-living canids are often influenced by motivation and context, for example, by reproductive activity [24,25], and as a result tend to deviate more from the expected linear hierarchy [19].

We employ social network analysis to investigate patterns of aggression, ritualized dominance (here defined as ritualized behaviours intended to assert dominance without resorting to aggression) and submission behaviours. Specifically, we (1) construct social networks based on aggressive, ritualized dominance and submissive behaviours, (2) test how ritualized dominance and aggressive behaviours vary with social rank, (3) determine regions of instability in the network and (4) examine whether rank instability is costly to individuals through increasing the frequency of aggressive interactions. Our study of social behaviour in dogs, where dominance is conspicuous and the costs of aggression can include prolonged, energetically costly interactions (such as chasing and physical fighting [19]) that carry a potential risk of injury [21], provides evidence for greater instability in dominance relationships and increased aggression in the centre of dominance hierarchies. We suggest that the patterns exhibited by dogs living in a complex social network may be a feature of groups composed of animals of different ages and sexes, and have important implications for the evolution of behavioural strategies within such groups, by generating rank-specific variation in the benefits of hierarchy formation.

## 2. Material and methods

### (a) Study system

Behavioural observations were conducted on a free-living pack of domestic dogs in Rome, Italy between April 2005 and May 2006 (197 days of observations in total). Individuals in the pack were not owned by humans, nor did they socialize with humans, and so they could move and breed freely, but were dependent on humans for food (provided daily by volunteer dog caretakers). Over the course of the study, pack size ranged from 25 to 40 dogs. Our analysis focused on the 27 individuals that remained in the pack long enough to provide sufficient behavioural data, comprising six adult males, five adult females, four subadult males, one subadult female, six juvenile males and five juvenile females. The age of individual dogs was ascertained from the knowledge of when they were born, if this was known. When not known, age was estimated for trapped individuals by local veterinary public health officials using standard veterinary methods (e.g. status of fur and tooth wear), or by trained field observers using physical characteristics (e.g. individuals that were not fully grown when first seen were aged as juveniles, while individuals with worn teeth or grey muzzle hair were aged as adults) [19].

### (b) Data collection

Behavioural observations were carried out in three different social contexts: in the presence of food, in the presence of receptive females and in the absence of any source of competition [19]. Data were collected using (i) a focal animal sampling method in the absence of sources of competition, (ii) a subgroup animal sampling method was used in the presence of food and receptive females (totalling 282.5 h of observation), and (iii) an ad libitum sampling method for behavioural interactions occurring outside focal sampling sessions, which were considered important for the aim of the study (totalling 630.4 h of observation) [19]. Focal observations of each individual were equally distributed over that full study period, as well as across daytime between 06.00 and 18.00 h. Aggressive behaviour was defined as threats (pointing, staring at, curling of the lips, baring of the canines, raising the hackles, snarling, growling and barking), chasing, physical fighting and biting. Ritualized dominance behaviour included individuals displaying an upright and stiff body posture with the head and tail held high and the ears pricked, individuals tail wagging with the tail held high and individuals placing their muzzle or paw on another individual's back. Submissive behaviour (often associated with threats) comprised avoiding eye contact, holding the head down, flattening the ears, holding the tail down or tightly between the hind legs and against the belly, cringing, lying down and exposing the ventral side of the chest or abdomen, avoiding and retreating. For all behavioural interactions, the initiator and recipient of the behaviour were recorded.

Directed and undirected networks for these three behavioural categories were calculated separately. Undirected networks used the total frequency of interactions between two individuals (i.e. the total number of interactions, regardless of initiator/receptor) to capture differences in the amount that different pairs of individuals interacted. Directed networks connected the initiator of a behaviour to its receptor. Both binary (whether an interaction occurred or not) and weighted (frequency of interactions) versions of the directed networks were analysed.

### (c) Calculation of rank

The social rank of individuals was calculated according to the methods of [26], using data on all submissive interactions. Submissive interactions provide the clearest distinction of ‘winners’ and ‘losers’ and have been used in other studies in social canids, including this study system [19]. This method uses an algorithm that seeks to minimize the number of inconsistencies in the rank order of individuals (i.e. where an individual of lower rank in a dyad wins more interactions than the higher-ranking individual) and the strength of these inconsistencies (the difference in rank between two individuals in an inconsistent dyad).

### (d) Social network analysis

Exponential random graph models were fitted to networks of interactions. These model the probability of an interaction occurring (binary networks) or the frequency of interactions (weighted networks) as a function of structural properties of the network, traits of the individuals (nodes) and of the relationships between them (edges) [27,28]. We fitted two models for each of our three behavioural categories containing a mixture of structural and individual-based terms: (i) a model of the binary directed network using individual attributes (sex and age) to explain the interactions an individual initiates, and (ii) a model of the weighted directed network using individual attributes (sex and age) to explain the interactions that an individual initiates. We then fitted two additional models to networks of ritualized dominance and aggressive interactions: (iii) a model of the weighted directed network using rank (as calculated using non-network methods as above) to explain the interactions that an individual initiates, and (iv) a model of the weighted undirected network using rank to explain the frequency of interactions between dyads. We did not fit these latter models to submissive interaction networks, as these data were used to assign the social ranks used as explanatory variables in them. Exponential random graph models (ERGMs) were fitted in R 3.2.0 [29] using the packages *ergm* [30,31] and *ergm.count* [32], following the methods of [33]. Statistical inference was based on the results from the full models. Model convergence was tested using the function *mcmc.diagnostics* [33]. Full details of the models are provided in the electronic supplementary material.

We then used model iii (rank-based) to explore how the ability to predict dominance-related interactions changed across the hierarchy. The model was used to simulate 1000 directed networks for aggressive and ritualized dominance interactions using the function *simulate* in *ergm*.*count* [32]. This uses the parameters of the fitted model to simulate networks with equivalent structural properties and enables the identification of regions of the network that are least well explained by the model. The proportion of behaviours performed by the more dominant individual in each dyad was then calculated for all null networks. This statistic calculated from all simulated networks in which an interaction took place was then compared to the equivalent proportion in the observed network, and the median value of this comparison provided a measure of model of goodness of fit that was used to determine how rank affected hierarchy stability. Goodness of fit provided a measure of how well the model was able to predict the initiators of behavioural interactions for dyads differing (a) in their position in the dominance hierarchy and (b) in their relative difference in rank. This provided a measure of how well hierarchical relationships in particular regions of the hierarchy matched the overall model, with the model over-fitting unstable regions and under-fitting regions of increased stability.

## 3. Results

### (a) The structure of free-living dog social networks

**Figure 1.**
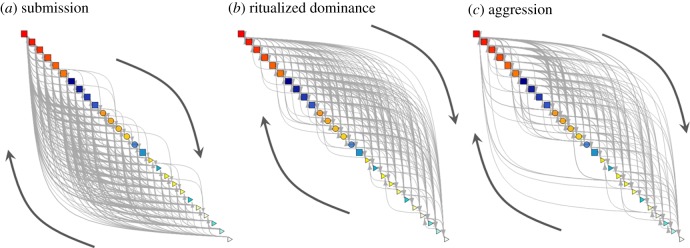
Directed networks of agonistic behaviour in a pack of free-living dogs, for (*a*) submissive interactions, (*b*) ritualized dominance interactions and (*c*) aggressive interactions. Edges are weighted in proportion to the frequency of interactions. Nodes are coloured according to sex (males are red/yellow and females are blue/green) and shaded to represent position in a hierarchy quantified using submissive interactions. Square nodes represent adults, circles are subadults and triangles are juveniles.

We identified evidence for a sex–age-graded linear dominance hierarchy from directed networks of submissive interactions (figure 1). For all three interaction networks, transitive interactions were significantly more likely and cyclical interactions significantly less likely than expected by chance, and this influenced both the probability of interactions occurring and the frequency of these interactions (table 1). Networks of submissive interactions were most linear, having the most negative estimates for cyclical interactions (meaning there were fewer triads where A > B, B > C and C > A) and a significant negative estimate for reciprocity (meaning there were fewer dyads where both individuals initiated a behaviour). As expected, networks of aggressive interactions were the least linear, showing more cyclical and reciprocal interactions than either of the ritualized dominance or submissive networks (least negative estimates for cyclical interactions and a positive rather than a negative estimate for mutual interactions). Adults occupied the top ranks of a hierarchy based on submissive interactions and tended to perform the most aggressive and ritualized dominance behaviours and the fewest submissive behaviours, directing their submissive behaviours more towards other adults. Juveniles occupied the bottom ranks of this hierarchy and initiated the fewest aggressive and ritualized dominance interactions and the most submissive interactions, directing ritualized dominance and aggressive interactions more towards other juveniles. Males within each age class occupied higher ranks than females, and tended to perform more ritualized dominance and fewer submissive behaviours than females, with their submissive interactions more likely to be directed at other males.

**Table 1.** Summary of variation in the probability and frequency of submissive, ritualized dominance and aggressive interactions in directed networks of free-living dog social interactions. Positive model estimates for the probability models mean that a given network configuration occurs more than expected, and positive estimates in the frequency models mean given network configurations have greater edge weights than expected. Negative model estimates mean that given network configurations occur less (probability model) or have lower edge weights (frequency model) than expected. Mutual terms were not fitted in the final weighted models as they caused the models to fail to converge. Estimates that were significant are in italics (with asterisks showing the level of significance, *p < 0.05, **p < 0.01, ***p < 0.001).

	submissive interactions		dominance interactions		aggressive interactions	
term	probability	frequency	probability	frequency	probability	frequency
transitive interactions	0.07 ± 0.01***	0.07 ± 0.02***	0.07 ± 0.01***	0.14 ± 0.04***	0.07 ± 0.01***	0.12 ± 0.03***
cyclical interactions	−0.61 ± 0.09***	−0.36 ± 0.03***	−0.47 ± 0.09***	−0.35 ± 0.04***	−0.18 ± 0.08*	−0.11 ± 0.03***
mutual interactions	−1.52 ± 0.41***	n.a.	−0.47 ± 0.40	n.a.	0.43 ± 0.34	n.a.
node match: age—adult	−0.28 ± 0.31	0.23 ± 0.10*	−0.15 ± 0.34	0.04 ± 0.03	−1.20 ± 0.28***	0.003 ± 0.03
node match: age—juvenile	−0.57 ± 0.35	−0.13 ± 0.04**	1.59 ± 1.02	1.58 ± 0.95	2.34 ± 0.56***	0.69 ± 0.18***
node match: age—subadult	2.51 ± 0.63***	0.04 ± 0.02	1.46 ± 0.63*	0.21 ± 0.05***	1.57 ± 0.61*	0.20 ± 0.04***
node match: sex—female	−1.04 ± 0.31***	−0.03 ± 0.04	−0.19 ± 0.29	0.004 ± 0.07	−0.51 ± 0.26	0.03 ± 0.08
node match: sex—male	1.07 ± 0.33**	0.13 ± 0.02***	0.26 ± 0.27	0.09 ± 0.03**	1.28 ± 0.24***	0.50 ± 0.08***
interactions: male versus female	−0.28 ± 0.14*	−0.02 ± 0.02	0.46 ± 0.20*	0.12 ± 0.04**	−0.84 ± 0.23***	−0.16 ± 0.09
interactions: juvenile versus adult	0.64 ± 0.19***	0.23 ± 0.10*	−3.20 ± 1.02**	−1.99 ± 0.95*	−3.25 ± 0.55***	−0.81 ± 0.18***
interactions: subadult versus adult	0.38 ± 0.16*	0.23 ± 0.10*	−0.46 ± 0.22*	−0.14 ± 0.04***	−0.82 ± 0.26**	−0.12 ± 0.04**

Subadults targeted aggression, ritualized dominance and submission disproportionately towards other subadults (significant node match: age—subadult terms). Although males typically out-ranked females of the same age class in hierarchies based on submissive interactions, they tended to initiate aggressive interactions towards fewer different individuals than females, and those that were initiated were targeted predominantly at other males.

### (b) Variation in hierarchy stability according to rank and behaviour

**Figure 2.**
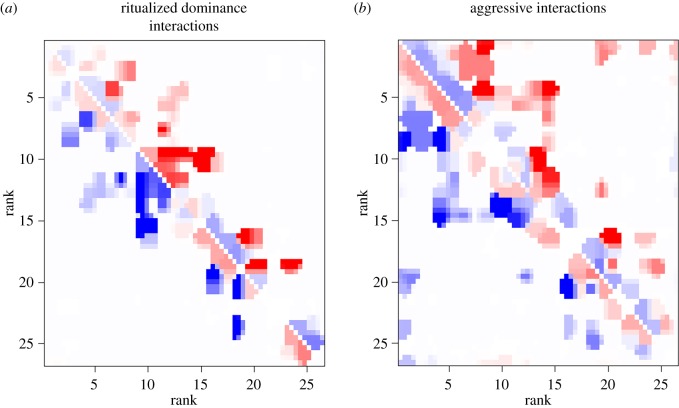
Similarity in the proportion of (*a*) ritualized dominance and (*b*) aggressive interactions initiated by an individual in a pack of free-living dogs when compared with networks simulated from rank-based exponential random graph models. Goodness of fit of the observed data to the simulated network model is the median difference between proportion of behaviours initiated in the observed network and 1000 simulated networks. Red represents initiations of behaviour being more likely in the observed network than simulated networks and blue the initiations of interactions being less likely.

Overall, simulated networks of behavioural interactions, using dominance ranks based on submissive behaviour, accurately predicted the initiation of other dominance interactions, especially for ritualized dominance behaviours. For all types of interaction, the goodness of fit for predicted initiations from these simulations was, however, lowest for individuals that were close in rank (figure 2). For aggressive interactions, reduced goodness of fit extended to individuals further apart in rank than for dominance interactions. For ritualized dominance interactions, when two individuals were adjacent in rank, the higher-ranked individual was often more likely to initiate a behaviour than the modelled expectations. By contrast, when two individuals were close but not adjacent in rank and were towards the centre of the hierarchy, the expected individual was less likely to initiate a ritualized dominance interaction than expected (figure 2*a*,*b*).

Networks of aggressive interactions were harder to predict accurately, and there was less systematic variation in when individuals did not behave as expected (figure 2*c*). However, there was some tendency for the expected (higher-ranking) individual to initiate fewer aggressive interactions than expected towards the top of the hierarchy, and for dyads further apart, than for ritualized dominance interactions, which may reflect the fact that males are less likely to initiate aggressive interactions than females.

### (c) Effects of rank on the levels of dominance behaviour and aggression

**Figure 3.**
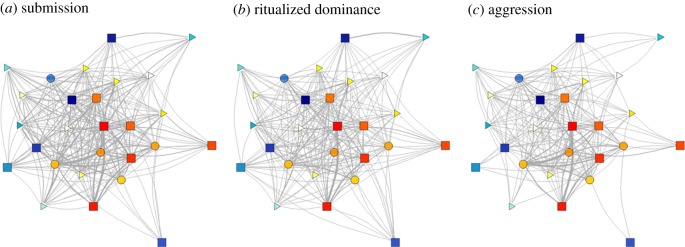
Undirected networks showing the frequency of behavioural interactions in a pack of free-living dogs for (*a*) submissive, (*b*) ritualized dominance and (*c*) aggressive interactions. Edges are weighted in proportion to the frequency of interactions. Nodes are coloured according to sex (males are red/yellow and females are blue/green) and shaded to represent position in the hierarchy quantified using submissive interactions. Square nodes represent adults, circles subadults and triangles juveniles.

**Figure 4.**
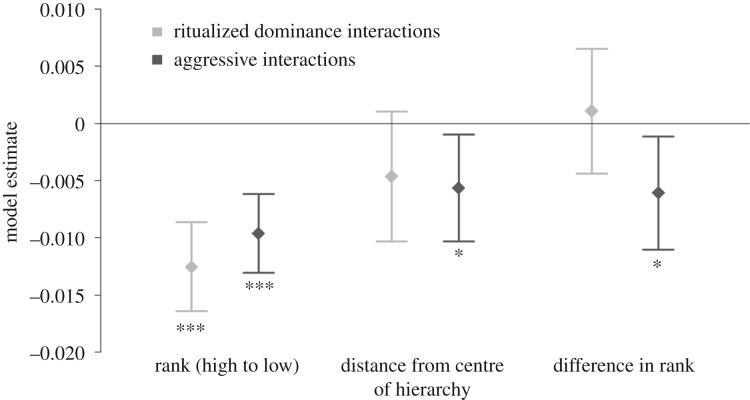
The effect of rank, rank distance from the centre of the hierarchy and difference in rank between two individuals on the frequency of involvement in ritualized dominance and aggressive interactions in a pack of free-living dogs. Models are from undirected networks of dominance-related interactions, and therefore individuals are recorded as interacting if they either initiated or were the recipient of a behaviour. Points represent the conditional estimates from the model and the error bars are the 95% confidence intervals of these estimates. Model estimates below zero mean that a change in the covariate reduces the number of interactions expected, and model estimates above zero mean that a change in the covariate increases the number of interactions expected.

Analysis of undirected interaction networks of ritualized dominance and aggressive interactions (figure 3) revealed that for both behaviours, interactions tended to be more frequent for dogs closer to the top of the hierarchy. For aggressive interactions, the frequency of interactions was also higher for individuals closer to the middle of the hierarchy and closer in rank (figure 4).

## 4. Discussion

Our network analysis found that the structure of dog hierarchy was less stable for individuals close, but not adjacent, to one another in rank, especially in the central region of the hierarchy. This central region of the hierarchy was characterized by elevated aggression that is likely to reduce the benefits of hierarchical living, leading to heterogeneity in the benefits obtained from hierarchy formation, and representing a cost of ascending rank in groups without strong reproductive skew.

As predicted, networks of all agonistic interactions showed elevated transitivity and reduced cyclicity of interactions as would be expected from a linear social hierarchy [13], and patterns in the frequency or strength and assortativity of interactions were almost universally supportive of the sex–age-graded model of dominance relationships applying to this population [19]. In other social canids, social hierarchies are also often influenced by sex and age [23,34]. Wolves differ in having hierarchies independently for males and females although still graded by age, especially when packs are small and closely related [23]. Dominance hierarchies are important in determining access to resources in free-living dogs [35], perhaps because of their promiscuous mating system [18] and tendency to live in unrelated as well as related groups [14,15]. In our study population, for example, dominant individuals were occasionally observed stealing food from subordinates, with no behavioural reaction from the subordinate individual [19].

Patterns of aggressive interactions (and to a lesser extent ritualized dominance interactions) were less transitive and more cyclical than those of submissive interactions, suggesting that they are be more dependent on context and motivation, and not always strictly tests of dominance. In this population, aggressive interactions are rarely initiated in the absence of a focus for competition, such as food [19]. Contrary to the expected pattern in vertebrate societies [36], female dogs tended to be aggressive to a greater number of different individuals than males. We also found that males aimed the bulk of their aggressive and ritualized dominance behaviour at other males, avoiding overtly aggressive encounters with females. Anecdotal observations support this pattern: males in a different group of free-ranging dogs were reported to ‘withdraw when the female made claims concerning food or a resting site’ [24]. Further, reduced male aggression has also been demonstrated in other social canids [37]. The targeting of aggression towards other males might also be expected if affiliative/non-aggressive social relationships increased breeding opportunities in a pack that is promiscuous [18]. In this situation, the costs of overt aggression are greater for males than females, according to the ‘docile male hypothesis', that postulates that male aggression toward females can harm reproductive success in some social systems [38–40].

We also showed how hierarchy stability varied with both rank, and difference in rank, for both ritualized dominance and aggressive behaviours. In general, the initiation of aggressive interactions was harder to predict than that of ritualized dominance interactions. This highlights that not all aggressive behaviour is related to dominance interactions in this system [19], and suggests that aggression is more likely for less well-established dominance relationships. For individuals immediately adjacent in rank, the initiation of interactions (ritualized dominance and aggressive) tended to be more one-sided than predicted by models, with the expected individual being more likely to initiate an interaction than anticipated, suggestive of winner–loser effects mediating dyadic behaviour among the most closely matched individuals [41,42]. By contrast, for individuals close in rank, but not adjacent to one another, in the central region of the hierarchy, dyadic relationships were less stable than would be expected. This difference in dyadic relationships between individuals adjacent in rank and those close but not adjacent in rank, would most likely be explained by individuals not adjacent in rank remaining relatively well matched, but having reduced information about their ‘opponent's’ relative strength or motivation to challenge [43]. These unstable regions may therefore arise as a consequence of temporal or contextual variation in factors associated with the initiation or outcome of contests [41–43]. In these free-living dogs, instability in this region of the hierarchy may be explained by it containing predominantly subadult individuals that are still establishing their dominance relationships, as is described in other canids [44]. This is supported by the tendency for subadults to target more dominance interactions (of all types) at other subadult individuals.

Our results indicate that regions of instability in a dominance hierarchy may undermine the benefit of reduced aggression for the individuals occupying those regions and may generate differences among individuals in the benefits obtained from hierarchy formation. Previous theoretical models have suggested that aggression should be greatest among dominant individuals as the benefits of gaining rank are greater [5], and that aggression can be used as a threat by dominant individuals to deter dominance challenges [12]. The behavioural [5,12,45] and consequent physiological [46,47] costs of maintaining dominance are well established, and in this pack of free-ranging dogs, high social rank was associated with an increased frequency of involvement in all types of behavioural interaction. However, in our study, the central region of the hierarchy, in which hierarchical relationships were most difficult to predict and less stable than expected, was also associated with elevated frequencies of aggressive interactions. Therefore, for individuals of middling rank, rank instability and its associated high levels of aggression may be an unavoidable cost incurred in moving up the ranks and progressing towards higher social status.

The impact of rank stability is likely to vary depending on the nature of dominance hierarchies. Many mammalian societies, especially those with more stable groups, are characterized by matrilineal hierarchies in which changes in dominance are highly unusual [48]. However, a similar elevation of aggression among middle-ranking individuals has been found in birds, in the sociable weaver *Philetairus socius* [49], and was suggested to be generated by either the increased benefits of improved rank, or as a result of more numerous social relationships. Similarly, in the cichlid fish *Neolamprologus pulcher*, increases in social rank were found to be associated with temporary increases in aggression [45]. It is therefore clear that across a taxonomically diverse range of societies, high levels of aggression can be seen away from the top of hierarchies, and that this variation in the expression of aggression is related not solely to ascent in rank but to instability and uncertainty in the dynamics of hierarchical relationships. Further work determining how this is related to the nature and fluidity of social structure would be highly valuable, and this would benefit greatly from analytical approaches that can incorporate modelling of the dynamics of dominance hierarchies [50,51].

We propose three mechanisms that may explain the pattern of instability in dyadic dominance relationships in these free-living dogs. First, reduced stability might occur because less information is available to assess dyadic relationships in a particular region of a hierarchy. Hierarchical relationships tend to be more stable when individuals have more information available to assess interaction outcomes [43,52]. As highlighted, in our hierarchy of free-living dogs, the unstable central region of the hierarchy was dominated by subadult individuals, and it might be expected that these individuals are still in the process of forming their social relationships. Second, if RHP (or a trait that correlates with RHPs, such as body size) is normally distributed then we expect a preponderance of dyads with reduced RHP asymmetries in the centre of a hierarchy. This may be analogous to the suggestion that social relationships are more complex and numerous in the central part of a hierarchy [49]. Third, the central region might represent an area where dyadic dominance relationships are highly dynamic and either social relationships within dyads change faster than it is possible to measure, or these dynamic social relationships result in less accurate information about the relative RHP of individuals. This is likely to be especially true if RHPs peak at a particular age before declining [53]. Since the unstable central region of the dominance hierarchy in our study pack consists primarily of subadult individuals, this third mechanism is perhaps less likely than those discussed previously.

We have revealed reduced linearity of dominance relationships and elevated aggression for middle-ranking individuals. The pattern of elevated aggression in the central region of a dominance hierarchy ran contrary to theoretical models of animal conflict developed for animal societies with high reproductive skew, in which aggression is expected to increase with hierarchical rank. Therefore, our results suggest that individuals in hierarchical societies, especially those with low reproductive skew, pay an unavoidable cost in order to assess social relationships, if or when they progress to higher ranks. A more general understanding of the roles of dominance relationships in mediating the costs of group living requires theoretical frameworks and empirical approaches that recognize dominance relationships as dynamic entities.

## Supplementary Material

Supplementary Methods and Code

## Supplementary Material

Supplementary Data

## Data accessibility

Data and R code are provided in the electronic supplementary material.
