# Supplementary material for: Elevated aggression is associated with uncertainty in a network of dog dominance interactions
Source: Proc Biol Sci. 2019 Jul 3;286(1906):20190536. doi: 10.1098/rspb.2019.0536 (PMC6650704; doi:10.1098/rspb.2019.0536)

Dog dominance hierarchy - ERGM models

Matthew Silk

19 July 2018

Supplementary methods

Exponential Random Graph Model details

Model i) used the terms ctriple, ttriple, mutual, nodematch(“age”, diff=TRUE), nodematch(“sex”, diff=TRUE), nodeofactor(“sex”) and nodeofactor(“age”).

Model ii) used the terms nonzero, sum, cyclicalweights(twopath = “min”, combine=“max”,affect=“min”), transitiveweights (twopath=“min”, combine=“max”, affect=“min”), nodematch(“age”, diff=TRUE), nodematch(“sex”, diff=TRUE), nodeofactor(“sex”) and nodeofactor(“age”). A geometric reference distribution for edge weight was used.

Model iii) used the terms nonzero, sum, cyclicalweights(twopath = “min”, combine=“max”,affect=“min”), transitiveweights (twopath=“min”, combine=“max”, affect=“min”), nodeocov(“rank”), absdiff(“rank”) and nodeocov(“centrank”). A Geometric reference distribution for edge weight was used.

Model iv) used the terms nonzero, sum, nodeocov(“rank”), absdiff(“rank”) and nodeocov(“distance from centre of rank”). A Geometric reference distribution for edge weight was used.

The term “non-zero” was included in models of weighted networks to control for zero-inflation generated by the networks being relatively sparse and the term “sum” was included as the equivalent to the intercept in a GLM. The terms “ttriple” and “ctriple” and “transitiveweights()” and “cyclicalweights()” model the number of transitive and cyclical triads in binary and weighted networks respectively. The term “mutual” models the likelihood of mutual interactions (i.e. A interacts with B and B interacts with A) in binary networks. “Nodematch” is used to model the assortativity according to the trait included as an argument. The additional argument diff=TRUE results in different parameters being estimated for each value of a trait. “Nodeofactor” models the differences in the probability/weight of interactions initiated among levels of a factor. “Nodeocov” models the effect of a continuous trait on the probability/weight of interactions an individual initiates. “Absdiff” models the probability/weight of an edge according to the difference in the values of a covariate between two individual in a dyad.

R code

Set path

```
direc<-"C:/Users/matth/Dropbox/BADGERS/dogs/"
```

Load packages required for initial data preparation

```
set.seed(12)

##load sna for network regressions
library(sna)
library(tnet)
library(ergm)
library(ergm.count)
library(latticeExtra)
library(fields)
```

Read in attribute data

```
#read in attribute data
attributes<-read.csv(paste0(direc,"attributedat.csv"),header=T)
names(attributes)<-c("ID","Rank","Sex","AgeClass","Age")

ranksquared<-attributes$Rank^2
centrank<-abs(attributes$Rank-median(attributes$Rank))
centrankcat<-c(rep(1,9),rep(2,9),rep(3,9))
centrankcat2<-c(rep(1,9),rep(2,9),rep(1,9))
```

Read in dominance matrix and turn it into a network object

```
data<-read.csv(paste0(direc,"DogDatadommat.csv"),header=T)
data<-as.matrix(data[,2:28])
rownames(data)<-colnames(data)
dom<-data
domint.edgelist<-as.tnet(data)
domint<-network(domint.edgelist[,1:2])

network::set.edge.attribute(domint,"weight",as.vector(domint.edgelist[,3]))

network::set.vertex.attribute(domint,"rank",as.vector(attributes$Rank))
network::set.vertex.attribute(domint,"ranksq",as.vector(ranksquared))
network::set.vertex.attribute(domint,"rankce",as.vector(centrank))
network::set.vertex.attribute(domint,"sex",as.numeric(attributes$Sex))
network::set.vertex.attribute(domint,"age",as.numeric(attributes$AgeClass))
network::set.vertex.attribute(domint,"rankcecat",as.vector(centrankcat))
network::set.vertex.attribute(domint,"rankcecat2",as.vector(centrankcat2))
```

Read in aggression matrix and turn it into a network object

```
data<-read.csv(paste0(direc,"DogDataagmat.csv"),header=T)
data<-as.matrix(data[,2:28])
rownames(data)<-colnames(data)
ag<-data
agint.edgelist<-as.tnet(data)
agint<-network(agint.edgelist[,1:2])

network::set.edge.attribute(agint,"weight",as.vector(agint.edgelist[,3]))

network::set.vertex.attribute(agint,"rank",as.vector(attributes$Rank))
network::set.vertex.attribute(agint,"ranksq",as.vector(ranksquared))
network::set.vertex.attribute(agint,"rankce",as.vector(centrank))
network::set.vertex.attribute(agint,"sex",as.numeric(attributes$Sex))
network::set.vertex.attribute(agint,"age",as.numeric(attributes$AgeClass))
network::set.vertex.attribute(agint,"age2",as.numeric(as.numeric(attributes$AgeClass)>1)+1)
network::set.vertex.attribute(agint,"rankcecat",as.vector(centrankcat))
network::set.vertex.attribute(agint,"rankcecat2",as.vector(centrankcat2))
```

Read in the submission matrix and turn into a network object

```
data<-read.csv(paste0(direc,"DogDatasubmat.csv"),header=T)
data<-as.matrix(data[,2:28])
rownames(data)<-colnames(data)
sub<-data
subint.edgelist<-as.tnet(data)
subint<-network(subint.edgelist[,1:2])

network::set.edge.attribute(subint,"weight",as.vector(subint.edgelist[,3]))

network::set.vertex.attribute(subint,"rank",as.vector(attributes$Rank))
network::set.vertex.attribute(subint,"ranksq",as.vector(ranksquared))
network::set.vertex.attribute(subint,"rankce",as.vector(centrank))
network::set.vertex.attribute(subint,"sex",as.numeric(attributes$Sex))
#nb age is now just adult or not adult as three cats cause problems
network::set.vertex.attribute(subint,"age",as.numeric(attributes$AgeClass))
network::set.vertex.attribute(subint,"age2",as.numeric(as.numeric(attributes$AgeClass)>1)+1)
network::set.vertex.attribute(subint,"rankcecat",as.vector(centrankcat))
network::set.vertex.attribute(subint,"rankcecat2",as.vector(centrankcat2))
```

Read in proportion matrices and rank differences

```
##read in proportion matrices
propag<-read.csv(paste0(direc,"agpropmat.csv"),header=T)
propag<-as.matrix(propag[,2:28])
rownames(propag)<-colnames(propag)
propsub<-read.csv(paste0(direc,"subpropmat.csv"),header=T)
propsub<-as.matrix(propsub[,2:28])
rownames(propsub)<-colnames(propsub)
propdom<-read.csv(paste0(direc,"dompropmat.csv"),header=T)
propdom<-as.matrix(propdom[,2:28])
rownames(propdom)<-colnames(propdom)

##read in rank differences and attributes
rds<-read.csv(paste0(direc,"rankdiffmat.csv"),header=T)
rds<-abs(as.matrix(rds[,2:28]))
rownames(rds)<-colnames(rds)
rds<-rds/max(rds)
```

Now set up matrices of outcome differences (prop matrices - 0.5)

```
outdom<-array(0,dim=c(27,27))
outag<-array(0,dim=c(27,27))
outsub<-array(0,dim=c(27,27))

for(i in 1:27){
for(j in 1:27){

if(dom[i,j]>0|dom[j,i]>0){
outdom[i,j]<-abs(propdom[i,j]-0.5)
}
if(ag[i,j]>0|ag[j,i]>0){
outag[i,j]<-abs(propag[i,j]-0.5)
}
if(sub[i,j]|sub[j,i]>0){
outsub[i,j]<-abs(propsub[i,j]-0.5)
}

}
}

##create undirected sum interaction matrices for all interactions actally
##they will be handy

sumag<-array(0,dim=dim(ag))

for (i in 1:length(ag[,1])){
   for (j in 1:length(ag[,1])){
      sumag[i,j]<-ag[i,j]+ag[j,i]
   }
}

sumag2<-sumag/max(sumag)

sumdom<-array(0,dim=dim(dom))

for (i in 1:length(dom[,1])){
   for (j in 1:length(dom[,1])){
      sumdom[i,j]<-dom[i,j]+dom[j,i]
   }
}

sumdom2<-sumdom/max(sumdom)

sumsub<-array(0,dim=dim(sub))

for (i in 1:length(sub[,1])){
   for (j in 1:length(sub[,1])){
      sumsub[i,j]<-sub[i,j]+sub[j,i]
   }
}

sumsub2<-sumsub/max(sumsub)
```

Analysis time

Run binary ERGMs Then run MCMC diagnostics on these models

```
#starting binary (and in alphabetical order

FAb<-ergm(agint~ctriple+ttriple+mutual+nodematch("age",diff=TRUE)+nodematch("sex",diff=TRUE)+nodeofactor("se
x")+nodeofactor("age"),silent=TRUE)
FDb<-ergm(domint~ctriple+ttriple+mutual+nodematch("age",diff=TRUE)+nodematch("sex",diff=TRUE)+nodeofactor("s
ex")+nodeofactor("age"),silent=TRUE)
FSb<-ergm(subint~ctriple+ttriple+mutual+nodematch("age",diff=TRUE)+nodematch("sex",diff=TRUE)+nodeofactor("s
ex")+nodeofactor("age"),silent=TRUE)

mcmc.diagnostics(FAb)
```

```
## Sample statistics summary:
##
## Iterations = 16384:4209664
## Thinning interval = 1024
## Number of chains = 1
## Sample size per chain = 4096
##
## 1. Empirical mean and standard deviation for each variable,
##    plus standard error of the mean:
##
##                         Mean      SD Naive SE Time-series SE
## ctriple              -3.68506  28.112  0.43925        0.57266
## ttriple             -42.50879 178.981  2.79658        4.81801
## mutual               -0.70508   5.187  0.08105        0.10600
## nodematch.age.1       0.44922   5.011  0.07830        0.09838
## nodematch.age.2      -0.33203   5.240  0.08187        0.10762
## nodematch.age.3      -0.08984   1.823  0.02848        0.04406
## nodematch.sex.1      -0.27881   4.643  0.07254        0.08967
## nodematch.sex.2      -1.84204   7.382  0.11534        0.18838
## nodeofactor.sex.2    -1.87231   9.955  0.15555        0.24047
## nodeofactor.age.2    -0.31006   5.560  0.08688        0.10906
## nodeofactor.age.3    -1.26709   6.119  0.09561        0.14221
##
## 2. Quantiles for each variable:
##
##                     2.5%    25% 50% 75%  97.5%
## ctriple              -55  -24.0  -5  15  54.62
## ttriple             -361 -167.2 -54  71 331.00
## mutual               -11   -4.0  -1   3  10.00
## nodematch.age.1       -9   -3.0   0   4  10.00
## nodematch.age.2      -11   -4.0   0   3  10.00
## nodematch.age.3       -4   -1.0   0   1   3.00
## nodematch.sex.1       -9   -4.0   0   3   9.00
## nodematch.sex.2      -17   -7.0  -2   3  12.00
## nodeofactor.sex.2    -22   -9.0  -2   5  17.62
## nodeofactor.age.2    -11   -4.0  -1   3  11.00
## nodeofactor.age.3    -13   -6.0  -1   3  11.00
##
##
## Sample statistics cross-correlations:
##                       ctriple   ttriple    mutual nodematch.age.1
## ctriple             1.0000000 0.7334765 0.7941757     0.376854197
## ttriple             0.7334765 1.0000000 0.6636579     0.466652577
## mutual              0.7941757 0.6636579 1.0000000     0.334688161
## nodematch.age.1     0.3768542 0.4666526 0.3346882     1.000000000
## nodematch.age.2     0.2842815 0.4416537 0.4097691     0.027897579
## nodematch.age.3     0.2813294 0.1988923 0.2333042     0.002200378
## nodematch.sex.1     0.1810673 0.3090656 0.2205463     0.239364817
## nodematch.sex.2     0.6523086 0.7613557 0.5896821     0.311392272
## nodeofactor.sex.2 0.7178503 0.8345334 0.6543547     0.347235537
## nodeofactor.age.2 0.3875339 0.4113966 0.4573891     0.014982091
## nodeofactor.age.3 0.5377762 0.6062027 0.4721468     0.022110227
##                     nodematch.age.2 nodematch.age.3 nodematch.sex.1
## ctriple                  0.28428155      0.281329389      0.18106734
## ttriple                  0.44165369      0.198892254      0.30906557
## mutual                   0.40976913      0.233304198      0.22054632
```

```
## nodematch.age.1        0.02789758     0.002200378      0.23936482
## nodematch.age.2        1.00000000     0.015206963      0.24560501
## nodematch.age.3        0.01520696     1.000000000      0.01400611
## nodematch.sex.1        0.24560501     0.014006110      1.00000000
## nodematch.sex.2        0.30081767     0.117111274      0.03266551
## nodeofactor.sex.2      0.38996368     0.151325449      0.07563950
## nodeofactor.age.2      0.91349925    -0.001062584      0.24344160
## nodeofactor.age.3      0.11555025     0.310923000      0.12667774
##                   nodematch.sex.2 nodeofactor.sex.2 nodeofactor.age.2
## ctriple                0.65230861         0.7178503       0.387533884
## ttriple                0.76135569         0.8345334       0.411396623
## mutual                 0.58968206         0.6543547       0.457389075
## nodematch.age.1        0.31139227         0.3472355       0.014982091
## nodematch.age.2        0.30081767         0.3899637       0.913499250
## nodematch.age.3        0.11711127         0.1513254      -0.001062584
## nodematch.sex.1        0.03266551         0.0756395       0.243441598
## nodematch.sex.2        1.00000000         0.8017725       0.303797477
## nodeofactor.sex.2      0.80177247         1.0000000       0.366071272
## nodeofactor.age.2      0.30379748         0.3660713       1.000000000
## nodeofactor.age.3      0.47846819         0.5744505       0.060728592
##                   nodeofactor.age.3
## ctriple                  0.53777621
## ttriple                  0.60620266
## mutual                   0.47214677
## nodematch.age.1          0.02211023
## nodematch.age.2          0.11555025
## nodematch.age.3          0.31092300
## nodematch.sex.1          0.12667774
## nodematch.sex.2          0.47846819
## nodeofactor.sex.2        0.57445048
## nodeofactor.age.2        0.06072859
## nodeofactor.age.3        1.00000000
##
## Sample statistics auto-correlation:
## Chain 1
##               ctriple    ttriple     mutual nodematch.age.1
## Lag 0     1.000000000 1.00000000 1.00000000     1.000000000
## Lag 1024  0.204540994 0.46857546 0.21086124     0.202442971
## Lag 2048  0.096875347 0.24723803 0.09609342     0.062725756
## Lag 3072  0.027341902 0.12810446 0.04761282     0.032421362
## Lag 4096  0.002315820 0.07403845 0.02143814     0.023729955
## Lag 5120 -0.005606301 0.04583005 0.02228467     0.002064413
##          nodematch.age.2 nodematch.age.3 nodematch.sex.1 nodematch.sex.2
## Lag 0         1.00000000      1.00000000     1.000000000      1.00000000
## Lag 1024      0.23415754      0.41052730     0.208744309      0.42763853
## Lag 2048      0.08751909      0.16630644     0.039894009      0.21008338
## Lag 3072      0.03984154      0.07493558     0.014588192      0.10206086
## Lag 4096      0.02385250      0.03832280    -0.001386292      0.05190004
## Lag 5120      0.01887109      0.01582048     0.002341983      0.01910078
##          nodeofactor.sex.2 nodeofactor.age.2 nodeofactor.age.3
## Lag 0           1.00000000        1.00000000        1.00000000
## Lag 1024        0.36543776        0.19330032        0.33949450
## Lag 2048        0.17878846        0.06760340        0.15353185
## Lag 3072        0.08149799        0.01749925        0.05202817
## Lag 4096        0.04469651        0.01933844        0.02377459
## Lag 5120        0.01729030        0.01450697        0.03965704
##
## Sample statistics burn-in diagnostic (Geweke):
## Chain 1
##
## Fraction in 1st window = 0.1
## Fraction in 2nd window = 0.5
##
##           ctriple           ttriple            mutual   nodematch.age.1
##          -0.50095          -0.01929          -0.97307          -0.07029
##   nodematch.age.2   nodematch.age.3   nodematch.sex.1   nodematch.sex.2
##           0.37172           0.77129          -0.30355           0.15781
## nodeofactor.sex.2 nodeofactor.age.2 nodeofactor.age.3
##           0.25080          -0.13161           0.04085
##
## Individual P-values (lower = worse):
##           ctriple           ttriple            mutual   nodematch.age.1
##         0.6164049         0.9846108         0.3305175         0.9439623
```

```
##    nodematch.age.2    nodematch.age.3    nodematch.sex.1    nodematch.sex.2
##          0.7101020          0.4405354          0.7614693          0.8746065
## nodeofactor.sex.2 nodeofactor.age.2 nodeofactor.age.3
##          0.8019662          0.8952948          0.9674149
## Joint P-value (lower = worse):  0.8871169 .
```

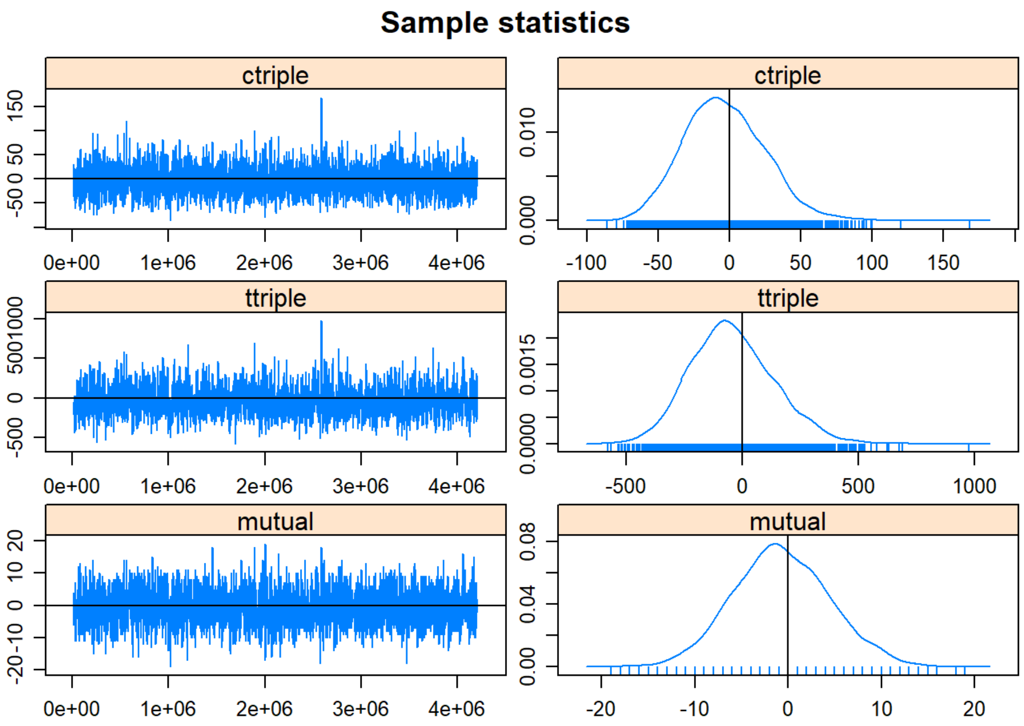

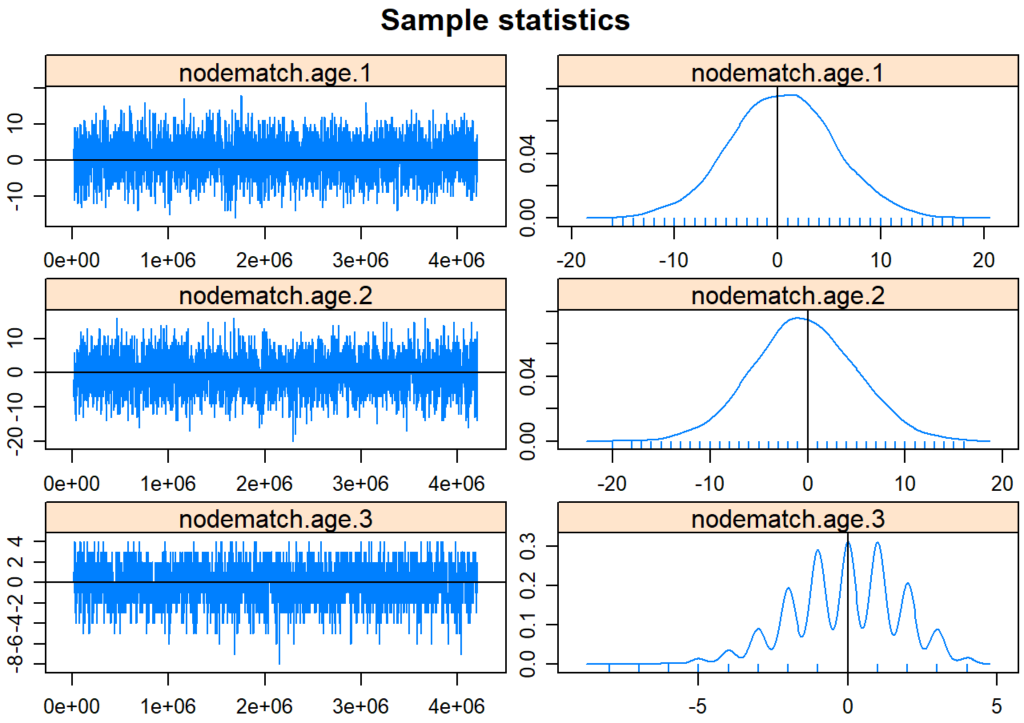

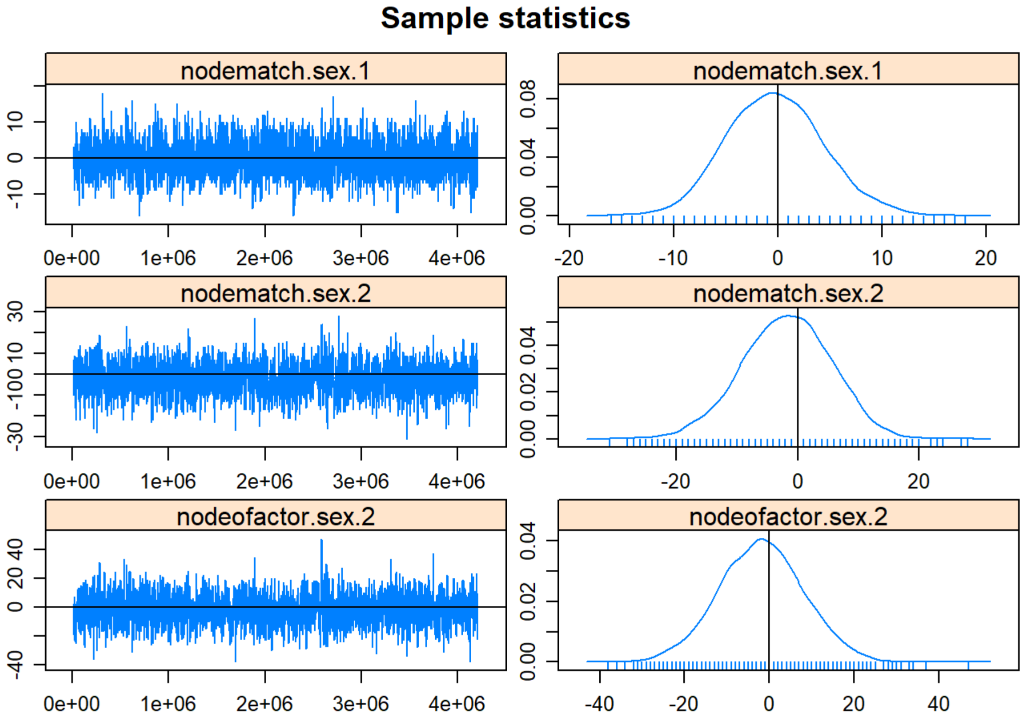

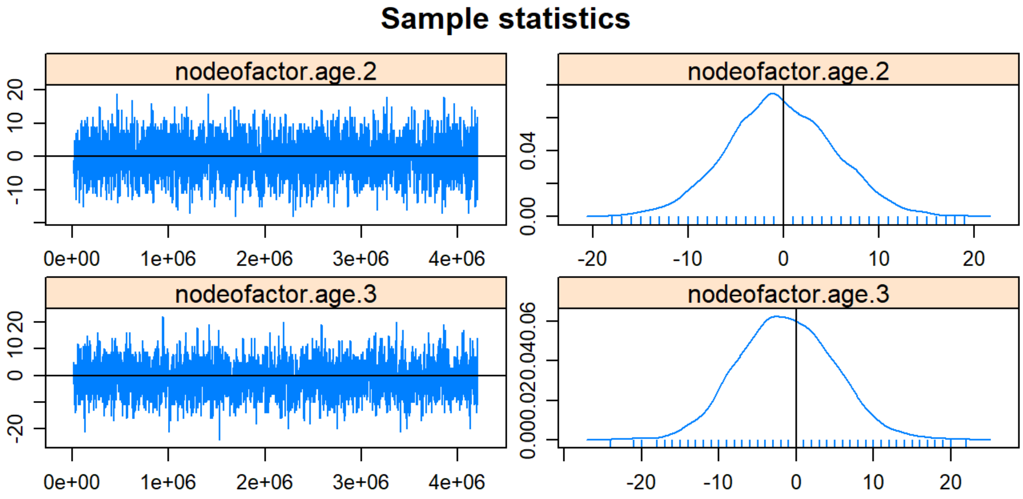


```
##
## MCMC diagnostics shown here are from the last round of simulation, prior to computation of final paramete
r estimates. Because the final estimates are refinements of those used for this simulation run, these diagno
stics may understate model performance. To directly assess the performance of the final model on in-model st
atistics, please use the GOF command: gof(ergmFitObject, GOF=~model).
```

```
mcmc.diagnostics(FDb)
```

```
## Sample statistics summary:
##
## Iterations = 16384:4209664
## Thinning interval = 1024
## Number of chains = 1
## Sample size per chain = 4096
##
## 1. Empirical mean and standard deviation for each variable,
##    plus standard error of the mean:
```

```
##
##                         Mean      SD Naive SE Time-series SE
## ctriple            0.09180  15.966  0.24947        0.24564
## ttriple            9.69385 181.138  2.83028        5.04410
## mutual             0.02075   3.705  0.05790        0.06378
## nodematch.age.1    0.30493   4.058  0.06340        0.09523
## nodematch.age.2   -0.14258   4.172  0.06518        0.07805
## nodematch.age.3   -0.17554   1.743  0.02724        0.04186
## nodematch.sex.1    0.30566   4.212  0.06581        0.09094
## nodematch.sex.2    0.38696   5.470  0.08547        0.13988
## nodeofactor.sex.2  1.12524   9.499  0.14842        0.29435
## nodeofactor.age.2 -0.06104   4.179  0.06530        0.07602
## nodeofactor.age.3  0.93555   6.617  0.10339        0.23741
##
## 2. Quantiles for each variable:
##
##                      2.5%  25% 50% 75% 97.5%
## ctriple            -28.0  -11  -1  10  34.0
## ttriple           -331.6 -117   7 125 382.2
## mutual              -7.0   -3   0   3   7.0
## nodematch.age.1     -8.0   -2   0   3   8.0
## nodematch.age.2     -8.0   -3   0   3   8.0
## nodematch.age.3     -4.0   -1   0   1   3.0
## nodematch.sex.1     -8.0   -3   0   3   9.0
## nodematch.sex.2    -11.0   -3   0   4  11.0
## nodeofactor.sex.2  -18.0   -5   1   8  19.0
## nodeofactor.age.2   -8.0   -3   0   3   8.0
## nodeofactor.age.3  -11.0   -4   1   5  15.0
##
##
## Sample statistics cross-correlations:
##                     ctriple   ttriple    mutual nodematch.age.1
## ctriple           1.0000000 0.3554145 0.6502944      0.28264183
## ttriple           0.3554145 1.0000000 0.4634229      0.51870325
## mutual            0.6502944 0.4634229 1.0000000      0.36524059
## nodematch.age.1   0.2826418 0.5187032 0.3652406      1.00000000
## nodematch.age.2   0.1410306 0.4570183 0.2842598      0.04597648
## nodematch.age.3   0.2150832 0.2048507 0.2688770      0.07142919
## nodematch.sex.1   0.1154377 0.4389114 0.1692717      0.27977711
## nodematch.sex.2   0.3289661 0.6310176 0.4003025      0.25661975
## nodeofactor.sex.2 0.2704692 0.7288806 0.3480408      0.32842597
## nodeofactor.age.2 0.1748823 0.4289366 0.2990046      0.03482317
## nodeofactor.age.3 0.3329232 0.3199634 0.2249716     -0.10834796
##                   nodematch.age.2 nodematch.age.3 nodematch.sex.1
## ctriple                0.14103062     0.215083179      0.11543773
## ttriple                0.45701832     0.204850689      0.43891142
## mutual                 0.28425979     0.268877004      0.16927167
## nodematch.age.1        0.04597648     0.071429190      0.27977711
## nodematch.age.2        1.00000000     0.016669751      0.23448041
## nodematch.age.3        0.01666975     1.000000000      0.01881548
## nodematch.sex.1        0.23448041     0.018815481      1.00000000
## nodematch.sex.2        0.32436715     0.199131918      0.04206604
## nodeofactor.sex.2      0.40212936     0.092428179      0.13962030
## nodeofactor.age.2      0.96584848    -0.004788877      0.22593264
## nodeofactor.age.3      0.09113194     0.084562260      0.13832209
##                   nodematch.sex.2 nodeofactor.sex.2 nodeofactor.age.2
## ctriple                0.32896607        0.27046925       0.174882313
## ttriple                0.63101765        0.72888060       0.428936627
## mutual                 0.40030253        0.34804079       0.299004569
## nodematch.age.1        0.25661975        0.32842597       0.034823173
## nodematch.age.2        0.32436715        0.40212936       0.965848476
## nodematch.age.3        0.19913192        0.09242818      -0.004788877
## nodematch.sex.1        0.04206604        0.13962030       0.225932638
## nodematch.sex.2        1.00000000        0.67060817       0.308351618
## nodeofactor.sex.2      0.67060817        1.00000000       0.399410322
## nodeofactor.age.2      0.30835162        0.39941032       1.000000000
## nodeofactor.age.3      0.25247610        0.48177537       0.073074458
##                   nodeofactor.age.3
## ctriple                  0.33292317
## ttriple                  0.31996344
## mutual                   0.22497162
## nodematch.age.1         -0.10834796
## nodematch.age.2          0.09113194
```

```
## nodematch.age.2          [illegible]
## nodematch.age.3          0.08456226
## nodematch.sex.1          0.13832209
## nodematch.sex.2          0.25247610
## nodeofactor.sex.2        0.48177537
## nodeofactor.age.2        0.07307446
## nodeofactor.age.3        1.00000000
##
## Sample statistics auto-correlation:
## Chain 1
##              ctriple    ttriple       mutual nodematch.age.1
## Lag 0     1.00000000 1.00000000  1.000000000      1.00000000
## Lag 1024  0.03819886 0.48373979  0.036269415      0.28772569
## Lag 2048  0.04101151 0.27207563  0.041889290      0.13288110
## Lag 3072 -0.01909780 0.15974965 -0.015469303      0.10271953
## Lag 4096  0.01236266 0.10116604  0.038208023      0.04755661
## Lag 5120 -0.02578096 0.04928758  0.007136189      0.03528181
##          nodematch.age.2 nodematch.age.3 nodematch.sex.1 nodematch.sex.2
## Lag 0        1.000000000      1.00000000      1.00000000      1.00000000
## Lag 1024     0.112171016      0.30616395      0.30845761      0.36188170
## Lag 2048     0.038674238      0.12660136      0.13101511      0.17257479
## Lag 3072     0.047355360      0.06540648      0.06020627      0.09247268
## Lag 4096     0.001223838      0.05354160      0.01650976      0.08630145
## Lag 5120    -0.013182692      0.05490493     -0.02642620      0.06038375
##          nodeofactor.sex.2 nodeofactor.age.2 nodeofactor.age.3
## Lag 0            1.0000000      1.0000000000         1.0000000
## Lag 1024         0.5143415      0.0980456457         0.5690317
## Lag 2048         0.3079396      0.0289826261         0.3645777
## Lag 3072         0.2025789      0.0380180798         0.2568963
## Lag 4096         0.1456322      0.0008690041         0.2006099
## Lag 5120         0.1025985     -0.0150683277         0.1599533
##
## Sample statistics burn-in diagnostic (Geweke):
## Chain 1
##
## Fraction in 1st window = 0.1
## Fraction in 2nd window = 0.5
##
##           ctriple           ttriple            mutual   nodematch.age.1
##           0.04577           1.40658           0.12702           1.25617
##   nodematch.age.2   nodematch.age.3   nodematch.sex.1   nodematch.sex.2
##           0.66897           2.30972           0.64712           1.28646
## nodeofactor.sex.2 nodeofactor.age.2 nodeofactor.age.3
##           1.14636           0.82707          -0.22142
##
## Individual P-values (lower = worse):
##           ctriple           ttriple            mutual   nodematch.age.1
##        0.96349328        0.15955142        0.89892411        0.20905595
##   nodematch.age.2   nodematch.age.3   nodematch.sex.1   nodematch.sex.2
##        0.50351537        0.02090348        0.51755485        0.19828331
## nodeofactor.sex.2 nodeofactor.age.2 nodeofactor.age.3
##        0.25164578        0.40819775        0.82476370
## Joint P-value (lower = worse):  0.4411865 .
```

Sample statistics

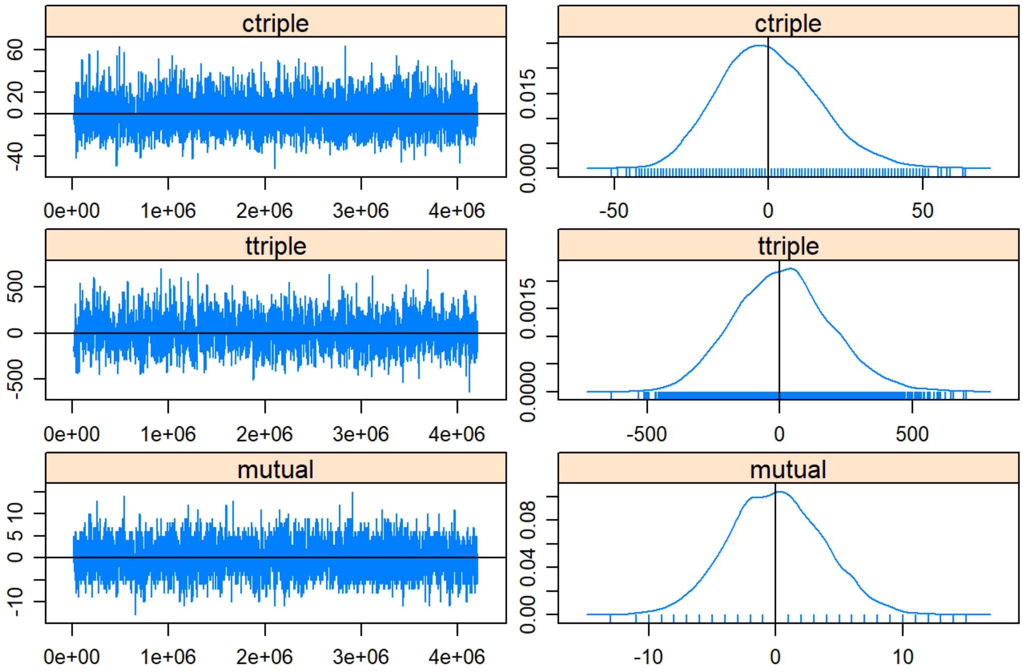

Sample statistics

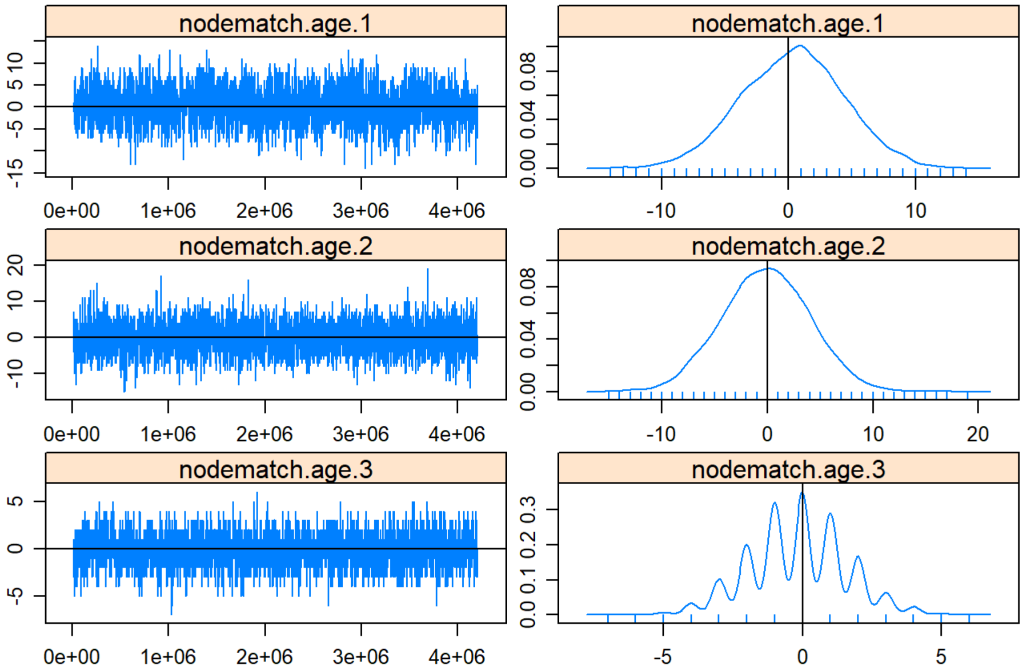

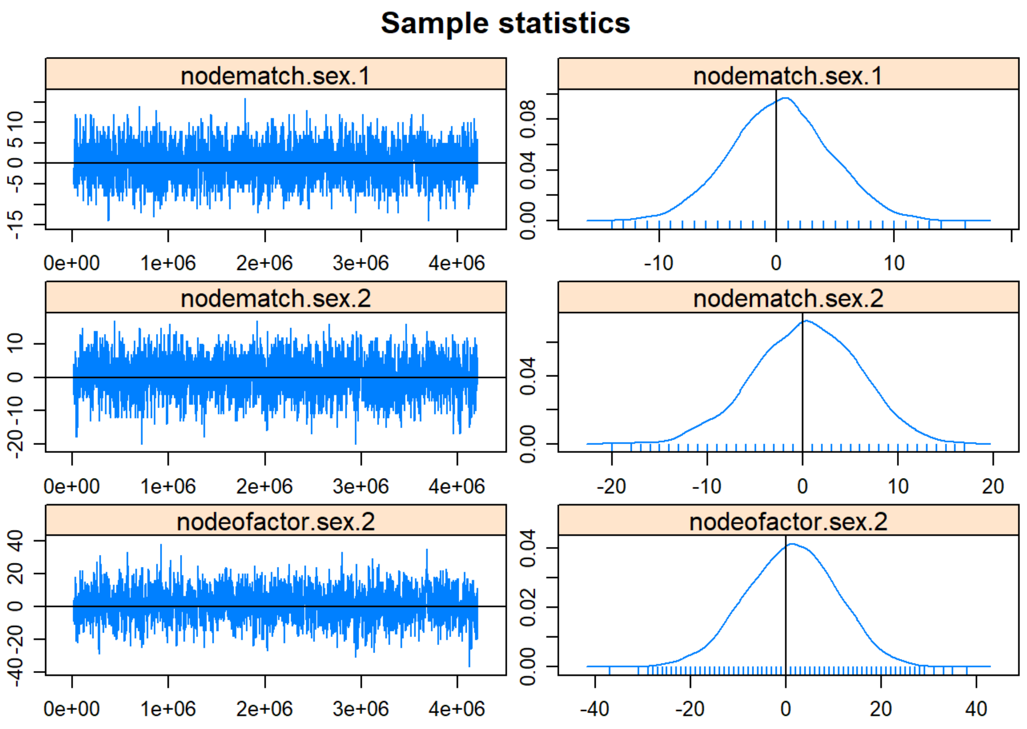

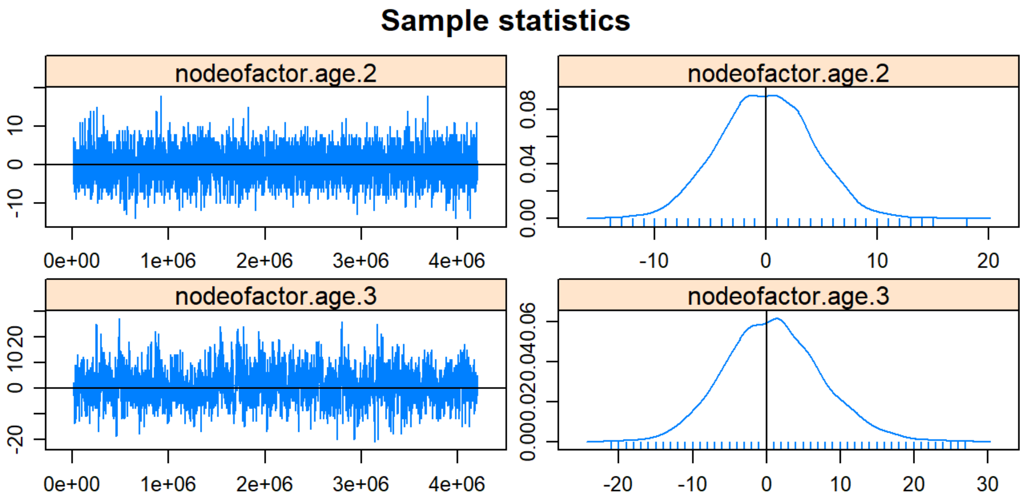


```
##
## MCMC diagnostics shown here are from the last round of simulation, prior to computation of final paramete
r estimates. Because the final estimates are refinements of those used for this simulation run, these diagno
stics may understate model performance. To directly assess the performance of the final model on in-model st
atistics, please use the GOF command: gof(ergmFitObject, GOF=~model).
```

```
mcmc.diagnostics(FSb)
```

```
## Sample statistics summary:
##
## Iterations = 16384:4209664
## Thinning interval = 1024
## Number of chains = 1
## Sample size per chain = 4096
##
## 1. Empirical mean and standard deviation for each variable,
##    plus standard error of the mean:
```

```
##
##                        Mean      SD Naive SE Time-series SE
## ctriple             1.06348  13.400  0.20938        0.24025
## ttriple           -11.25708 173.425  2.70976        5.53062
## mutual             -0.16553   3.246  0.05071        0.05738
## nodematch.age.1    -0.07031   4.045  0.06320        0.10332
## nodematch.age.2    -0.07056   3.800  0.05937        0.10207
## nodematch.age.3    -0.28125   1.721  0.02688        0.07518
## nodematch.sex.1    -0.21436   4.216  0.06587        0.10051
## nodematch.sex.2    -0.14453   4.028  0.06295        0.09722
## nodeofactor.sex.2  -2.41919  10.256  0.16025        0.56342
## nodeofactor.age.2  -1.61230  10.899  0.17030        0.59346
## nodeofactor.age.3  -0.89233   9.806  0.15322        0.90789
##
## 2. Quantiles for each variable:
##
##                     2.5%  25% 50%   75% 97.5%
## ctriple            -23.0   -8   0  10.0  29.0
## ttriple           -341.6 -132 -14 103.2 338.6
## mutual              -6.0   -2   0   2.0   6.0
## nodematch.age.1     -8.0   -3   0   3.0   8.0
## nodematch.age.2     -8.0   -3   0   3.0   7.0
## nodematch.age.3     -3.0   -2   0   1.0   3.0
## nodematch.sex.1     -8.0   -3   0   3.0   8.0
## nodematch.sex.2     -8.0   -3   0   3.0   8.0
## nodeofactor.sex.2  -22.0  -10  -2   5.0  18.0
## nodeofactor.age.2  -24.0   -9  -1   6.0  19.0
## nodeofactor.age.3  -20.0   -7  -1   6.0  19.0
##
##
## Sample statistics cross-correlations:
##                       ctriple   ttriple     mutual nodematch.age.1
## ctriple            1.00000000 0.1641710 0.52889881      0.04824304
## ttriple            0.16417100 1.0000000 0.39132119      0.48423287
## mutual             0.52889881 0.3913212 1.00000000      0.16270194
## nodematch.age.1    0.04824304 0.4842329 0.16270194      1.00000000
## nodematch.age.2    0.08067448 0.5087657 0.21991306      0.02626034
## nodematch.age.3    0.23131707 0.2109271 0.33643206      0.09092349
## nodematch.sex.1    0.02040166 0.5153171 0.06686592      0.27567577
## nodematch.sex.2    0.29353914 0.5425568 0.44646920      0.26465374
## nodeofactor.sex.2  0.16216507 0.4127331 0.27165089      0.24498377
## nodeofactor.age.2 -0.01315865 0.5833160 0.13554602      0.15802819
## nodeofactor.age.3  0.11979919 0.1584871 0.13264148      0.08215104
##                   nodematch.age.2 nodematch.age.3 nodematch.sex.1
## ctriple                0.08067448      0.23131707      0.02040166
## ttriple                0.50876575      0.21092713      0.51531713
## mutual                 0.21991306      0.33643206      0.06686592
## nodematch.age.1        0.02626034      0.09092349      0.27567577
## nodematch.age.2        1.00000000      0.05318056      0.30869572
## nodematch.age.3        0.05318056      1.00000000      0.01431048
## nodematch.sex.1        0.30869572      0.01431048      1.00000000
## nodematch.sex.2        0.21361679      0.20024432      0.03071440
## nodeofactor.sex.2      0.13615746      0.21233156      0.03572755
## nodeofactor.age.2      0.55657710      0.14777662      0.39468031
## nodeofactor.age.3     -0.09531468      0.08539465      0.04429821
##                   nodematch.sex.2 nodeofactor.sex.2 nodeofactor.age.2
## ctriple                 0.2935391        0.16216507       -0.01315865
## ttriple                 0.5425568        0.41273312        0.58331603
## mutual                  0.4464692        0.27165089        0.13554602
## nodematch.age.1         0.2646537        0.24498377        0.15802819
## nodematch.age.2         0.2136168        0.13615746        0.55657710
## nodematch.age.3         0.2002443        0.21233156        0.14777662
## nodematch.sex.1         0.0307144        0.03572755        0.39468031
## nodematch.sex.2         1.0000000        0.33064119        0.17265385
## nodeofactor.sex.2       0.3306412        1.00000000        0.34421296
## nodeofactor.age.2       0.1726538        0.34421296        1.00000000
## nodeofactor.age.3       0.1315534        0.33952669       -0.38965947
##                   nodeofactor.age.3
## ctriple                  0.11979919
## ttriple                  0.15848714
## mutual                   0.13264148
## nodematch.age.1          0.08215104
## nodematch.age.2         -0.09531468
```

```
## nodematch.age.2          [illegible]
## nodematch.age.3          0.08539465
## nodematch.sex.1          0.04429821
## nodematch.sex.2          0.13155340
## nodeofactor.sex.2        0.33952669
## nodeofactor.age.2       -0.38965947
## nodeofactor.age.3        1.00000000
##
## Sample statistics auto-correlation:
## Chain 1
##              ctriple    ttriple      mutual nodematch.age.1
## Lag 0     1.00000000 1.00000000 1.000000000      1.00000000
## Lag 1024  0.06537174 0.55148124 0.051719939      0.29307403
## Lag 2048  0.02791443 0.35082469 0.037110938      0.16220086
## Lag 3072  0.02093004 0.23506232 0.040235973      0.10820955
## Lag 4096  0.03308771 0.14349655 0.007723308      0.06476894
## Lag 5120 -0.00204429 0.09804802 0.007417317      0.03800778
##          nodematch.age.2 nodematch.age.3 nodematch.sex.1 nodematch.sex.2
## Lag 0         1.00000000       1.0000000      1.00000000     1.000000000
## Lag 1024      0.32076747       0.5264871      0.31107710     0.335785793
## Lag 2048      0.16890070       0.3838603      0.15114538     0.179687569
## Lag 3072      0.15257558       0.3262725      0.09905951     0.110047651
## Lag 4096      0.09751014       0.2858462      0.03699960     0.035960211
## Lag 5120      0.07964210       0.2355772      0.02374409     0.007075492
##          nodeofactor.sex.2 nodeofactor.age.2 nodeofactor.age.3
## Lag 0            1.0000000         1.0000000         1.0000000
## Lag 1024         0.7515315         0.7527169         0.8828030
## Lag 2048         0.6345445         0.6248365         0.8172255
## Lag 3072         0.5545079         0.5369555         0.7608812
## Lag 4096         0.4760706         0.4598680         0.7128541
## Lag 5120         0.4209009         0.4113329         0.6702470
##
## Sample statistics burn-in diagnostic (Geweke):
## Chain 1
##
## Fraction in 1st window = 0.1
## Fraction in 2nd window = 0.5
##
##           ctriple           ttriple            mutual   nodematch.age.1
##            0.3549           -0.3462            0.6857            0.3277
##   nodematch.age.2   nodematch.age.3   nodematch.sex.1   nodematch.sex.2
##           -0.8710            1.2522           -0.5232           -0.6131
## nodeofactor.sex.2 nodeofactor.age.2 nodeofactor.age.3
##            0.8574           -1.4778            1.4574
##
## Individual P-values (lower = worse):
##           ctriple           ttriple            mutual   nodematch.age.1
##         0.7226801         0.7291766         0.4928937         0.7431185
##   nodematch.age.2   nodematch.age.3   nodematch.sex.1   nodematch.sex.2
##         0.3837326         0.2104906         0.6008604         0.5397998
## nodeofactor.sex.2 nodeofactor.age.2 nodeofactor.age.3
##         0.3912244         0.1394575         0.1450078
## Joint P-value (lower = worse):  0.60375 .
```

Sample statistics

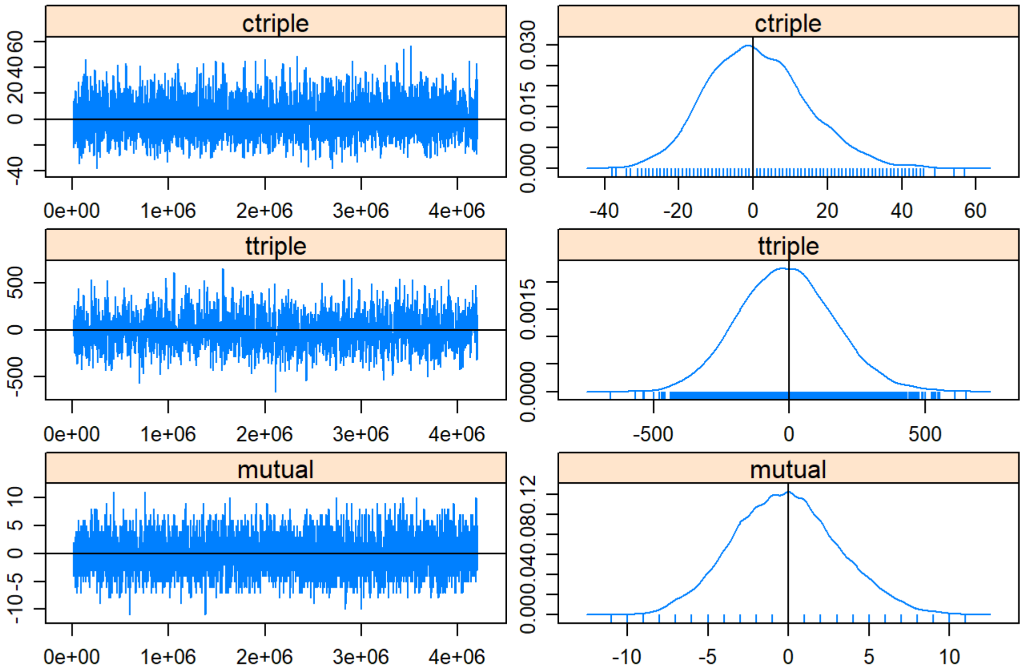

Sample statistics

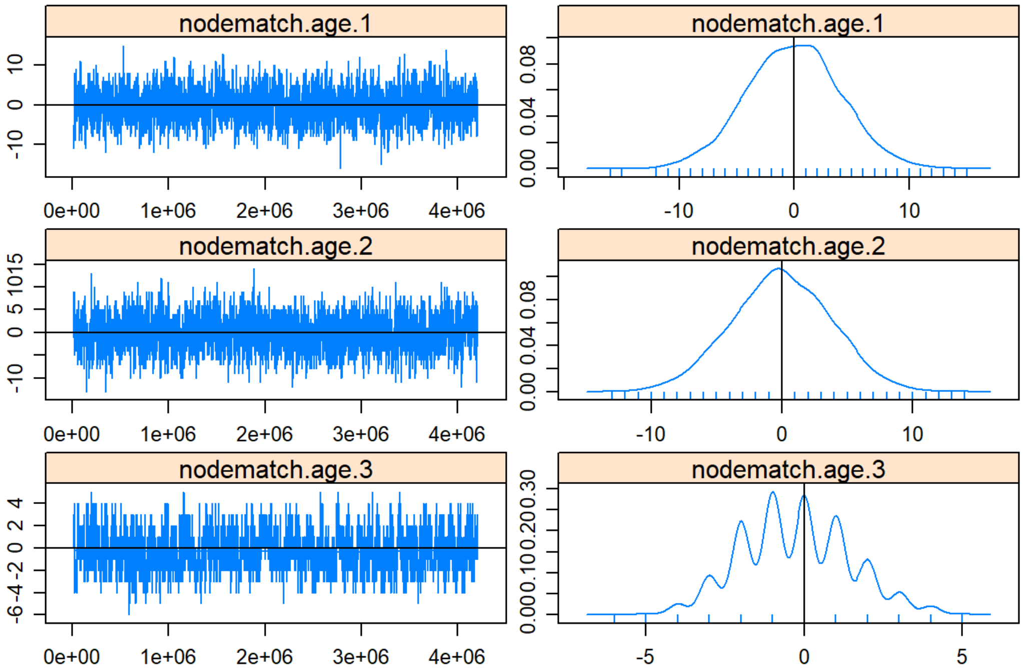

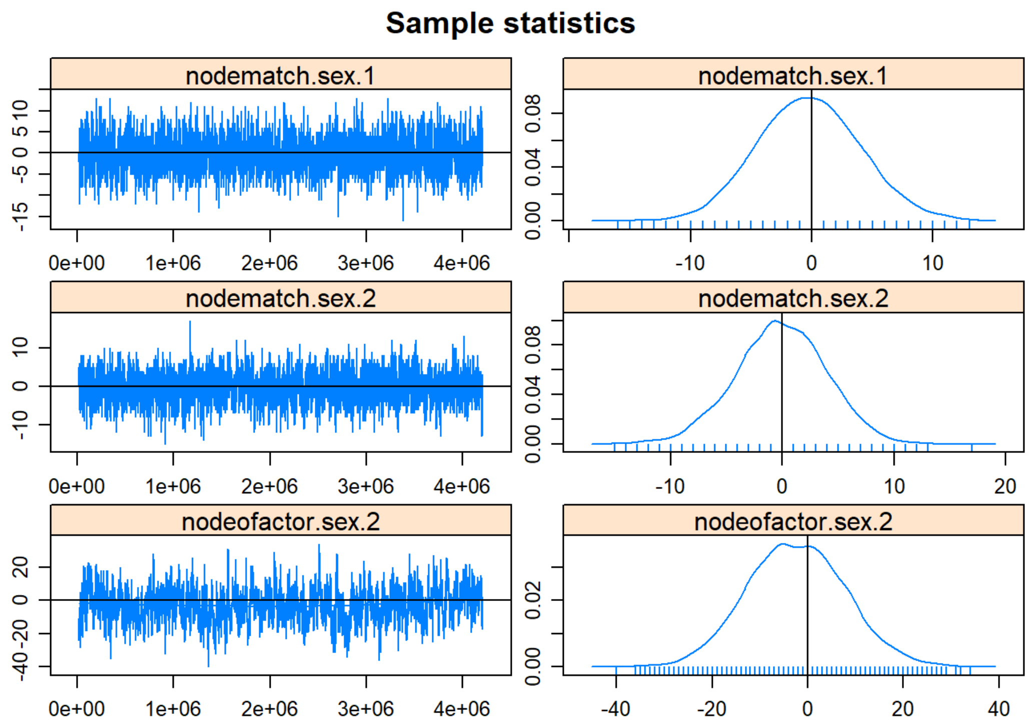

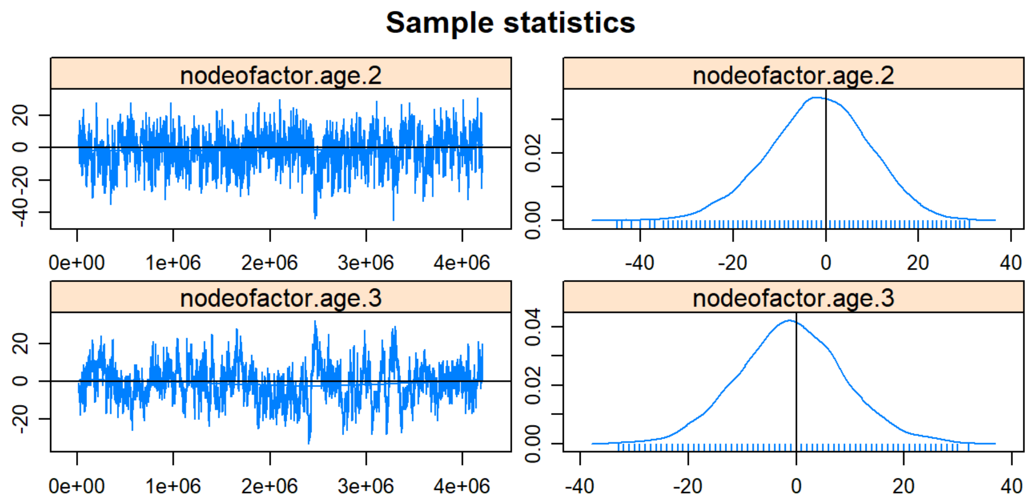


```
##
## MCMC diagnostics shown here are from the last round of simulation, prior to computation of final paramete
r estimates. Because the final estimates are refinements of those used for this simulation run, these diagno
stics may understate model performance. To directly assess the performance of the final model on in-model st
atistics, please use the GOF command: gof(ergmFitObject, GOF=~model).
```

```
summary(FDb)
```

```
##
## ==========================
## Summary of model fit
## ==========================
##
## Formula:   domint ~ ctriple + ttriple + mutual + nodematch("age", diff = TRUE) +
##     nodematch("sex", diff = TRUE) + nodeofactor("sex") + nodeofactor("age")
##
## Iterations:  3 out of 20
##
## Monte Carlo MLE Results:
##                   Estimate Std. Error MCMC %  p-value
## ctriple           -0.46640    0.08893      0  < 1e-04 ***
## ttriple            0.06757    0.01143      0  < 1e-04 ***
## mutual            -0.46258    0.39214      0 0.238559
## nodematch.age.1   -0.16006    0.34932      0 0.646954
## nodematch.age.2    1.64088    0.97077      0 0.091424 .
## nodematch.age.3    1.47390    0.61548      0 0.016900 *
## nodematch.sex.1   -0.19802    0.29240      0 0.498486
## nodematch.sex.2    0.24703    0.27623      0 0.371479
## nodeofactor.sex.2  0.45838    0.20083      0 0.022767 *
## nodeofactor.age.2 -3.25479    0.97584      0 0.000897 ***
## nodeofactor.age.3 -0.47597    0.21072      0 0.024209 *
## ---
## Signif. codes:  0 '***' 0.001 '**' 0.01 '*' 0.05 '.' 0.1 ' ' 1
##
##      Null Deviance: 973.2  on 702  degrees of freedom
##  Residual Deviance: 579.7  on 691  degrees of freedom
##
## AIC: 601.7    BIC: 651.8    (Smaller is better.)
```

```
summary(FAb)
```

```
##
## ==========================
## Summary of model fit
## ==========================
##
## Formula:   agint ~ ctriple + ttriple + mutual + nodematch("age", diff = TRUE) +
##     nodematch("sex", diff = TRUE) + nodeofactor("sex") + nodeofactor("age")
##
## Iterations:  4 out of 20
##
## Monte Carlo MLE Results:
##                   Estimate Std. Error MCMC %  p-value
## ctriple           -0.18525    0.07711      0 0.016550 *
## ttriple            0.06667    0.01449      0  < 1e-04 ***
## mutual             0.42868    0.34262      0 0.211288
## nodematch.age.1   -1.19693    0.27416      0  < 1e-04 ***
## nodematch.age.2    2.33164    0.54614      0  < 1e-04 ***
## nodematch.age.3    1.54227    0.59935      0 0.010283 *
## nodematch.sex.1   -0.50335    0.25344      0 0.047422 *
## nodematch.sex.2    1.28058    0.24626      0  < 1e-04 ***
## nodeofactor.sex.2 -0.83793    0.22391      0 0.000198 ***
## nodeofactor.age.2 -3.24729    0.53243      0  < 1e-04 ***
## nodeofactor.age.3 -0.80854    0.25947      0 0.001908 **
## ---
## Signif. codes:  0 '***' 0.001 '**' 0.01 '*' 0.05 '.' 0.1 ' ' 1
##
##      Null Deviance: 973.2  on 702  degrees of freedom
##  Residual Deviance: 667.0  on 691  degrees of freedom
##
## AIC: 689    BIC: 739.1    (Smaller is better.)
```

```
summary(FSb)
```

```
##
## ==========================
## Summary of model fit
## ==========================
##
## Formula:   subint ~ ctriple + ttriple + mutual + nodematch("age", diff = TRUE) +
##     nodematch("sex", diff = TRUE) + nodeofactor("sex") + nodeofactor("age")
##
## Iterations:  7 out of 20
##
## Monte Carlo MLE Results:
##                    Estimate Std. Error MCMC %  p-value
## ctriple            -0.61129    0.08952      0  < 1e-04 ***
## ttriple             0.06837    0.01233      0  < 1e-04 ***
## mutual             -1.51588    0.41152      0 0.000248 ***
## nodematch.age.1    -0.28327    0.30432      0 0.352259
## nodematch.age.2    -0.57088    0.34821      0 0.101567
## nodematch.age.3     2.53073    0.63245      0  < 1e-04 ***
## nodematch.sex.1    -1.02453    0.30932      0 0.000974 ***
## nodematch.sex.2     1.06985    0.33737      0 0.001586 **
## nodeofactor.sex.2 -0.26956    0.13875      0 0.052458 .
## nodeofactor.age.2  0.64469    0.18628      0 0.000572 ***
## nodeofactor.age.3  0.39133    0.15877      0 0.013951 *
## ---
## Signif. codes:  0 '***' 0.001 '**' 0.01 '*' 0.05 '.' 0.1 ' ' 1
##
##      Null Deviance: 973.2  on 702  degrees of freedom
##  Residual Deviance: 530.4  on 691  degrees of freedom
##
## AIC: 552.4    BIC: 602.5    (Smaller is better.)
```

Run weighted, directed ERGMs with individual traits Then examine convergence

```
FDg<-ergm(domint~sum+nonzero+cyclicalweights(twopath="min",combine="max",affect="min")+transitiveweights(two
path="min",combine="max",affect="min")+nodematch("age",diff=TRUE)+nodematch("sex",diff=TRUE)+nodeofactor("se
x")+nodeofactor("age"),reference=~Geometric,response="weight",silent=TRUE)
```

```
## Starting contrastive divergence estimation via CD-MCMLE:
```

```
## Iteration 1 of at most 60:
```

```
## Convergence test P-value:0e+00
```

```
## The log-likelihood improved by1.213
```

```
## Iteration 2 of at most 60:
```

```
## Convergence test P-value:4.2e-189
```

```
## The log-likelihood improved by1.031
```

```
## Iteration 3 of at most 60:
```

```
## Convergence test P-value:1.2e-66
```

```
## The log-likelihood improved by0.2254
```

```
## Iteration 4 of at most 60:
```

```
## Convergence test P-value:3.8e-20
```

```
## The log-likelihood improved by0.06332
```

```
## Iteration 5 of at most 60:
```

```
## Convergence test P-value:6e-05
```

```
## The log-likelihood improved by0.02012
```

```
## Iteration 6 of at most 60:
```

```
## Convergence test P-value:2.5e-01
```

```
## The log-likelihood improved by0.007304
```

```
## Iteration 7 of at most 60:
```

```
## Convergence test P-value:6e-01
```

```
## Convergence detected. Stopping.
```

```
## The log-likelihood improved by0.004952
```

```
## Starting maximum likelihood estimation via MCMLE:
```

```
## Iteration 1 of at most 20:
```

```
## Optimizing with step length 0.676175783058006.
```

```
## The log-likelihood improved by 2.554.
```

```
## Iteration 2 of at most 20:
```

```
## Optimizing with step length 1.
```

```
## The log-likelihood improved by 0.9989.
```

```
## Step length converged once. Increasing MCMC sample size.
```

```
## Iteration 3 of at most 20:
```

```
## Optimizing with step length 1.
```

```
## The log-likelihood improved by 0.06321.
```

```
## Step length converged twice. Stopping.
```

```
## Note: Null model likelihood calculation is not implemented for valued ERGMs at this time.
```

```
## Evaluating log-likelihood at the estimate. Using 20 bridges: 1 2 3 4 5 6 7 8 9 10 11 12 13 14 15 16 17 18
19 20 .
## This model was fit using MCMC.  To examine model diagnostics and check for degeneracy, use the mcmc.diagn
ostics() function.
```

```
FAg<-ergm(agint~sum+nonzero+cyclicalweights(twopath="min",combine="max",affect="min")+transitiveweights(twop
ath="min",combine="max",affect="min")+nodematch("age",diff=TRUE)+nodematch("sex",diff=TRUE)+nodeofactor("sex
")+nodeofactor("age"),reference=~Geometric,response="weight",silent=TRUE)
```

```
## Starting contrastive divergence estimation via CD-MCMLE:
```

```
## Starting contrastive divergence estimation via CD-MCMLE:
## Iteration 1 of at most 60:
## Convergence test P-value:0e+00
## The log-likelihood improved by1.436
## Iteration 2 of at most 60:
## Convergence test P-value:5e-183
## The log-likelihood improved by0.7815
## Iteration 3 of at most 60:
## Convergence test P-value:1.6e-66
## The log-likelihood improved by0.2212
## Iteration 4 of at most 60:
## Convergence test P-value:1.9e-11
## The log-likelihood improved by0.04413
## Iteration 5 of at most 60:
## Convergence test P-value:2.7e-06
## The log-likelihood improved by0.02573
## Iteration 6 of at most 60:
## Convergence test P-value:3.4e-02
## The log-likelihood improved by0.01037
## Iteration 7 of at most 60:
## Convergence test P-value:1.7e-01
## The log-likelihood improved by0.008314
## Iteration 8 of at most 60:
## Convergence test P-value:5.7e-01
## Convergence detected. Stopping.
## The log-likelihood improved by0.005227
## Starting maximum likelihood estimation via MCMLE:
## Iteration 1 of at most 20:
## Optimizing with step length 0.484115124907071.
## The log-likelihood improved by 3.112.
## Iteration 2 of at most 20:
## Optimizing with step length 0.165357774946163.
## The log-likelihood improved by 1.646.
## Iteration 3 of at most 20:
## Optimizing with step length 0.106472467473856.
## The log-likelihood improved by 1.908.
## Iteration 4 of at most 20:
## Optimizing with step length 0.0192295130494557.
## The log-likelihood improved by 0.9054.
## Iteration 5 of at most 20:
## Optimizing with step length 0.0560101182526424.
## The log-likelihood improved by 4.955.
## Iteration 6 of at most 20:
## Optimizing with step length 0.396523436239739.
## The log-likelihood improved by 2.603.
## Iteration 7 of at most 20:
## Optimizing with step length 0.92340929295428.
## The log-likelihood improved by 2.241.
## Iteration 8 of at most 20:
## Optimizing with step length 1.
## The log-likelihood improved by 2.439.
## Step length converged once. Increasing MCMC sample size.
## Iteration 9 of at most 20:
## Optimizing with step length 0.929278847841131.
## The log-likelihood improved by 3.729.
## Iteration 10 of at most 20:
## Optimizing with step length 0.090704307627167.
## The log-likelihood improved by 2.086.
## Iteration 11 of at most 20:
## Optimizing with step length 0.0127398362347362.
## The log-likelihood improved by 2.313.
## Iteration 12 of at most 20:
## Optimizing with step length 0.999999999999992.
## The log-likelihood improved by 0.4313.
## Iteration 13 of at most 20:
## Optimizing with step length 1.
## The log-likelihood improved by 2.443.
## Step length converged once. Increasing MCMC sample size.
## Iteration 14 of at most 20:
## Optimizing with step length 0.789020344386753.
## The log-likelihood improved by 5.978.
## Iteration 15 of at most 20:
## Optimizing with step length 0.201027151186728.
```

```
## The log-likelihood improved by 2.269.
## Iteration 16 of at most 20:
## Optimizing with step length 0.0223853552391763.
## The log-likelihood improved by 1.492.
## Iteration 17 of at most 20:
## Optimizing with step length 0.183479954847064.
## The log-likelihood improved by 1.376.
## Iteration 18 of at most 20:
## Optimizing with step length 0.492037788983597.
## The log-likelihood improved by 2.135.
## Iteration 19 of at most 20:
## Optimizing with step length 1.
## The log-likelihood improved by 0.2699.
## Iteration 20 of at most 20:
## Optimizing with step length 1.
## The log-likelihood improved by 0.1191.
## Step length converged once. Increasing MCMC sample size.
## MCMLE estimation did not converge after 20 iterations. The estimated coefficients may not be accurate. Es
timation may be resumed by passing the coefficients as initial values; see 'init' under ?control.ergm for de
tails.
## Note: Null model likelihood calculation is not implemented for valued ERGMs at this time.
## Evaluating log-likelihood at the estimate. Using 20 bridges: 1 2 3 4 5 6 7 8 9 10 11 12 13 14 15 16 17 18
19 20 .
## This model was fit using MCMC.  To examine model diagnostics and check for degeneracy, use the mcmc.diagn
ostics() function.
```

```
FSg<-ergm(subint~sum+nonzero+cyclicalweights(twopath="min",combine="max",affect="min")+transitiveweights(two
path="min",combine="max",affect="min")+nodematch("age",diff=TRUE)+nodematch("sex",diff=TRUE)+nodeofactor("se
x")+nodeofactor("age"),reference=~Geometric,response="weight",silent=TRUE)
```

```
## Starting contrastive divergence estimation via CD-MCMLE:
## Iteration 1 of at most 60:
## Convergence test P-value:0e+00
## The log-likelihood improved by1.444
## Iteration 2 of at most 60:
## Convergence test P-value:3.2e-191
## The log-likelihood improved by0.9577
## Iteration 3 of at most 60:
## Convergence test P-value:6e-35
## The log-likelihood improved by0.1168
## Iteration 4 of at most 60:
## Convergence test P-value:3.8e-09
## The log-likelihood improved by0.03466
## Iteration 5 of at most 60:
## Convergence test P-value:1.5e-02
## The log-likelihood improved by0.01273
## Iteration 6 of at most 60:
## Convergence test P-value:6.1e-02
## The log-likelihood improved by0.01034
## Iteration 7 of at most 60:
## Convergence test P-value:6.5e-01
## Convergence detected. Stopping.
## The log-likelihood improved by0.004776
## Starting maximum likelihood estimation via MCMLE:
## Iteration 1 of at most 20:
## Optimizing with step length 0.430317410088826.
## The log-likelihood improved by 3.767.
## Iteration 2 of at most 20:
## Optimizing with step length 0.422649330456232.
## The log-likelihood improved by 2.422.
## Iteration 3 of at most 20:
## Optimizing with step length 0.669219852573564.
## The log-likelihood improved by 1.9.
## Iteration 4 of at most 20:
## Optimizing with step length 1.
## The log-likelihood improved by 2.119.
## Step length converged once. Increasing MCMC sample size.
## Iteration 5 of at most 20:
## Optimizing with step length 1.
## The log-likelihood improved by 0.2013.
## Step length converged twice. Stopping.
## Note: Null model likelihood calculation is not implemented for valued ERGMs at this time.
## Evaluating log-likelihood at the estimate. Using 20 bridges: 1 2 3 4 5 6 7 8 9 10 11 12 13 14 15 16 17 18
19 20 .
## This model was fit using MCMC.  To examine model diagnostics and check for degeneracy, use the mcmc.diagn
ostics() function.
```

```
mcmc.diagnostics(FAg)
```

```
## Sample statistics summary:
##
## Iterations = 16384:1063936
## Thinning interval = 1024
## Number of chains = 1
## Sample size per chain = 1024
##
## 1. Empirical mean and standard deviation for each variable,
##    plus standard error of the mean:
##
##                                  Mean     SD Naive SE Time-series SE
## sum                            -7.859 105.67   3.3022        10.6659
## nonzero                         1.467  11.90   0.3717         0.6601
## cyclicalweights.min.max.min    15.068  53.60   1.6749         3.3477
## transitiveweights.min.max.min  -3.340  84.32   2.6351         7.7565
## nodematch.sum.age.1             4.312  39.33   1.2291         3.3662
## nodematch.sum.age.2             1.920  20.82   0.6505         1.2786
## nodematch.sum.age.3           -14.688  51.42   1.6070         7.4951
## nodematch.sum.sex.1            -0.250  14.69   0.4591         0.8533
## nodematch.sum.sex.2           -10.444 102.47   3.2023        10.2289
## nodeofactor.sum.sex.2          -8.446 102.73   3.2102        10.5124
## nodeofactor.sum.age.2           2.712  21.67   0.6772         1.3196
```

```
## nodeofactor.sum.age.3          -13.867  53.82   1.6818         6.3249
##
## 2. Quantiles for each variable:
##
##                                   2.5%    25% 50%   75%  97.5%
## sum                            -184.12 -80.25 -13 55.00 235.12
## nonzero                         -22.42  -6.25   1  9.00  26.00
## cyclicalweights.min.max.min     -85.00 -23.00  15 50.00 123.00
## transitiveweights.min.max.min -148.43 -62.25 -10 52.00 170.70
## nodematch.sum.age.1             -69.00 -21.00   2 28.25  87.42
## nodematch.sum.age.2             -35.00 -13.00   1 15.00  46.42
## nodematch.sum.age.3            -100.85 -50.25 -21 18.25  98.42
## nodematch.sum.sex.1             -28.00 -10.00  -1  9.00  30.42
## nodematch.sum.sex.2            -191.43 -84.00 -18 55.00 212.42
## nodeofactor.sum.sex.2          -186.85 -83.00 -13 54.25 211.42
## nodeofactor.sum.age.2           -38.42 -12.25   2 17.00  48.42
## nodeofactor.sum.age.3          -110.42 -51.00 -17 21.00  98.00
##
##
## Sample statistics cross-correlations:
##                                     sum     nonzero
## sum                            1.0000000  0.35531781
## nonzero                        0.3553178  1.00000000
## cyclicalweights.min.max.min    0.3451114  0.57664875
## transitiveweights.min.max.min 0.9032177  0.45098733
## nodematch.sum.age.1            0.5429080  0.23741135
## nodematch.sum.age.2            0.2645266  0.33080870
## nodematch.sum.age.3            0.3773168 -0.07808339
## nodematch.sum.sex.1            0.0713014  0.31891167
## nodematch.sum.sex.2            0.9608689  0.18670063
## nodeofactor.sum.sex.2          0.9690379  0.23110525
## nodeofactor.sum.age.2          0.2624674  0.41197576
## nodeofactor.sum.age.3          0.4656002  0.04653518
##                                cyclicalweights.min.max.min
## sum                                             0.34511144
## nonzero                                         0.57664875
## cyclicalweights.min.max.min                     1.00000000
## transitiveweights.min.max.min                   0.51251173
## nodematch.sum.age.1                             0.30419450
## nodematch.sum.age.2                             0.14022239
## nodematch.sum.age.3                             0.08881526
## nodematch.sum.sex.1                             0.18065355
## nodematch.sum.sex.2                             0.25646085
## nodeofactor.sum.sex.2                           0.28094865
## nodeofactor.sum.age.2                           0.23629964
## nodeofactor.sum.age.3                           0.19237494
##                                transitiveweights.min.max.min
## sum                                                0.9032177
## nonzero                                            0.4509873
## cyclicalweights.min.max.min                        0.5125117
## transitiveweights.min.max.min                      1.0000000
## nodematch.sum.age.1                                0.5355778
## nodematch.sum.age.2                                0.2043603
## nodematch.sum.age.3                                0.2993057
## nodematch.sum.sex.1                                0.0534777
## nodematch.sum.sex.2                                0.8606938
## nodeofactor.sum.sex.2                              0.8682231
## nodeofactor.sum.age.2                              0.2128206
## nodeofactor.sum.age.3                              0.3923257
##                                nodematch.sum.age.1 nodematch.sum.age.2
## sum                                    0.542907989          0.26452665
## nonzero                                0.237411348          0.33080870
## cyclicalweights.min.max.min            0.304194504          0.14022239
## transitiveweights.min.max.min          0.535577800          0.20436031
## nodematch.sum.age.1                    1.000000000          0.03555239
## nodematch.sum.age.2                    0.035552392          1.00000000
## nodematch.sum.age.3                   -0.009659267         -0.14890795
## nodematch.sum.sex.1                    0.109529646          0.17717584
## nodematch.sum.sex.2                    0.485414876          0.20023069
## nodeofactor.sum.sex.2                  0.493698340          0.21036689
## nodeofactor.sum.age.2                  0.052749311          0.95128217
## nodeofactor.sum.age.3                 -0.021709038         -0.09060799
##                                nodematch.sum.age.3 nodematch.sum.sex.1
```

```
##                             nodematch.sum.age.3 nodematch.sum.sex.1
## sum                                 0.377316841          0.07130140
## nonzero                            -0.078083392          0.31891167
## cyclicalweights.min.max.min         0.088815260          0.18065355
## transitiveweights.min.max.min       0.299305722          0.05347770
## nodematch.sum.age.1                -0.009659267          0.10952965
## nodematch.sum.age.2                -0.148907950          0.17717584
## nodematch.sum.age.3                 1.000000000         -0.11202457
## nodematch.sum.sex.1                -0.112024572          1.00000000
## nodematch.sum.sex.2                 0.406714695         -0.09118614
## nodeofactor.sum.sex.2               0.407029358         -0.08382285
## nodeofactor.sum.age.2              -0.149839175          0.20720649
## nodeofactor.sum.age.3               0.877515048         -0.05662279
##                             nodematch.sum.sex.2 nodeofactor.sum.sex.2
## sum                                  0.96086886            0.96903787
## nonzero                              0.18670063            0.23110525
## cyclicalweights.min.max.min          0.25646085            0.28094865
## transitiveweights.min.max.min        0.86069377            0.86822309
## nodematch.sum.age.1                  0.48541488            0.49369834
## nodematch.sum.age.2                  0.20023069            0.21036689
## nodematch.sum.age.3                  0.40671470            0.40702936
## nodematch.sum.sex.1                 -0.09118614           -0.08382285
## nodematch.sum.sex.2                  1.00000000            0.99121906
## nodeofactor.sum.sex.2                0.99121906            1.00000000
## nodeofactor.sum.age.2                0.18965582            0.20045779
## nodeofactor.sum.age.3                0.47183351            0.47781017
##                             nodeofactor.sum.age.2 nodeofactor.sum.age.3
## sum                                    0.26246742            0.46560016
## nonzero                                0.41197576            0.04653518
## cyclicalweights.min.max.min            0.23629964            0.19237494
## transitiveweights.min.max.min          0.21282056            0.39232566
## nodematch.sum.age.1                    0.05274931           -0.02170904
## nodematch.sum.age.2                    0.95128217           -0.09060799
## nodematch.sum.age.3                   -0.14983918            0.87751505
## nodematch.sum.sex.1                    0.20720649           -0.05662279
## nodematch.sum.sex.2                    0.18965582            0.47183351
## nodeofactor.sum.sex.2                  0.20045779            0.47781017
## nodeofactor.sum.age.2                  1.00000000           -0.10337861
## nodeofactor.sum.age.3                 -0.10337861            1.00000000
## 
## Sample statistics auto-correlation:
## Chain 1 
##                sum    nonzero cyclicalweights.min.max.min
## Lag 0    1.0000000 1.00000000                  1.00000000
## Lag 1024 0.8137588 0.51814022                  0.59927127
## Lag 2048 0.6531955 0.25952959                  0.36281238
## Lag 3072 0.5331233 0.15632928                  0.23249231
## Lag 4096 0.4210164 0.07594175                  0.13296456
## Lag 5120 0.3563935 0.03369456                  0.08234128
##          transitiveweights.min.max.min nodematch.sum.age.1
## Lag 0                        1.0000000           1.0000000
## Lag 1024                     0.7833385           0.7350577
## Lag 2048                     0.6303841           0.5710109
## Lag 3072                     0.5256577           0.4465428
## Lag 4096                     0.4088643           0.3480759
## Lag 5120                     0.3325141           0.2912322
##          nodematch.sum.age.2 nodematch.sum.age.3 nodematch.sum.sex.1
## Lag 0             1.00000000           1.0000000          1.00000000
## Lag 1024          0.58841047           0.8790465          0.55064232
## Lag 2048          0.35391245           0.7880896          0.32741812
## Lag 3072          0.22261445           0.7136793          0.19507935
## Lag 4096          0.12903241           0.6589797          0.12220250
## Lag 5120          0.09775321           0.6029253          0.09493025
##          nodematch.sum.sex.2 nodeofactor.sum.sex.2 nodeofactor.sum.age.2
## Lag 0              1.0000000             1.0000000             1.0000000
## Lag 1024           0.8351376             0.8291578             0.5827968
## Lag 2048           0.6839963             0.6739253             0.3355295
## Lag 3072           0.5693339             0.5584792             0.2023993
## Lag 4096           0.4610405             0.4484657             0.1056807
## Lag 5120           0.3828996             0.3750199             0.0706256
##          nodeofactor.sum.age.3
## Lag 0                1.0000000
## Lag 1024             0.8197069
```

```
## Lag 2048             0.6866847
## Lag 3072             0.5802730
## Lag 4096             0.5236052
## Lag 5120             0.4694548
##
## Sample statistics burn-in diagnostic (Geweke):
## Chain 1
##
## Fraction in 1st window = 0.1
## Fraction in 2nd window = 0.5
##
##                           sum                      nonzero
##                       -2.4915                      -1.5559
##   cyclicalweights.min.max.min transitiveweights.min.max.min
##                       -0.4665                      -1.6620
##           nodematch.sum.age.1          nodematch.sum.age.2
##                       -1.0579                      -0.4647
##           nodematch.sum.age.3          nodematch.sum.sex.1
##                       -0.4384                      -0.2033
##           nodematch.sum.sex.2         nodeofactor.sum.sex.2
##                       -2.2520                      -2.4390
##         nodeofactor.sum.age.2         nodeofactor.sum.age.3
##                       -0.2607                      -0.9869
##
## Individual P-values (lower = worse):
##                           sum                      nonzero
##                    0.01272080                   0.11973491
##   cyclicalweights.min.max.min transitiveweights.min.max.min
##                    0.64084906                   0.09651216
##           nodematch.sum.age.1          nodematch.sum.age.2
##                    0.29009976                   0.64213831
##           nodematch.sum.age.3          nodematch.sum.sex.1
##                    0.66111790                   0.83887834
##           nodematch.sum.sex.2         nodeofactor.sum.sex.2
##                    0.02431949                   0.01472600
##         nodeofactor.sum.age.2         nodeofactor.sum.age.3
##                    0.79432262                   0.32367159
## Joint P-value (lower = worse):  0.7466634 .
```

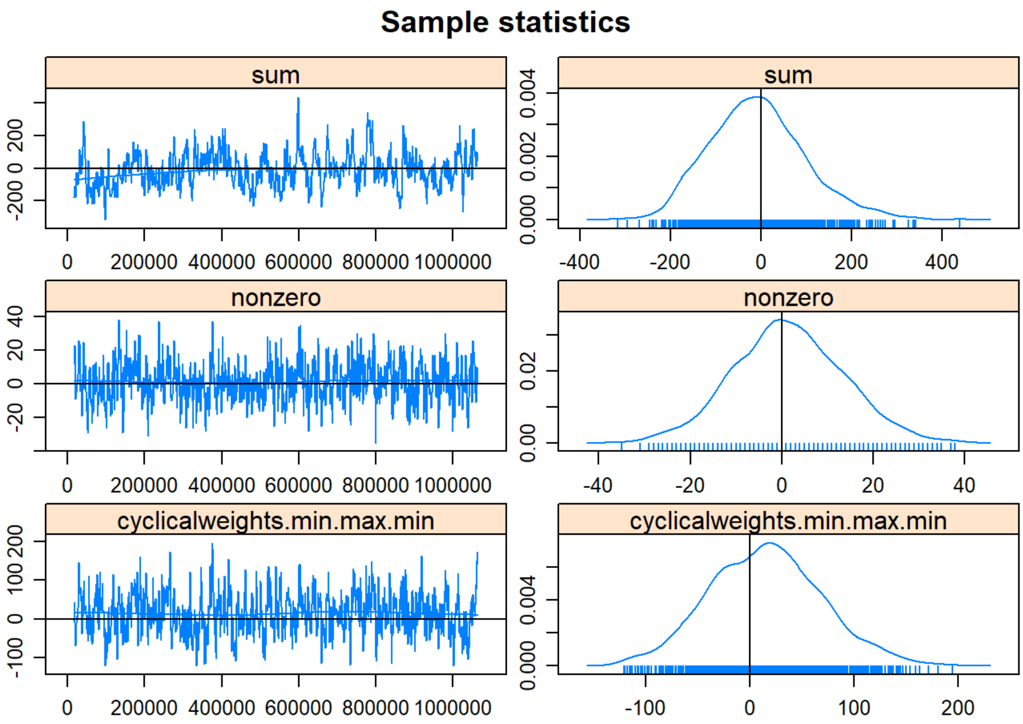

Sample statistics

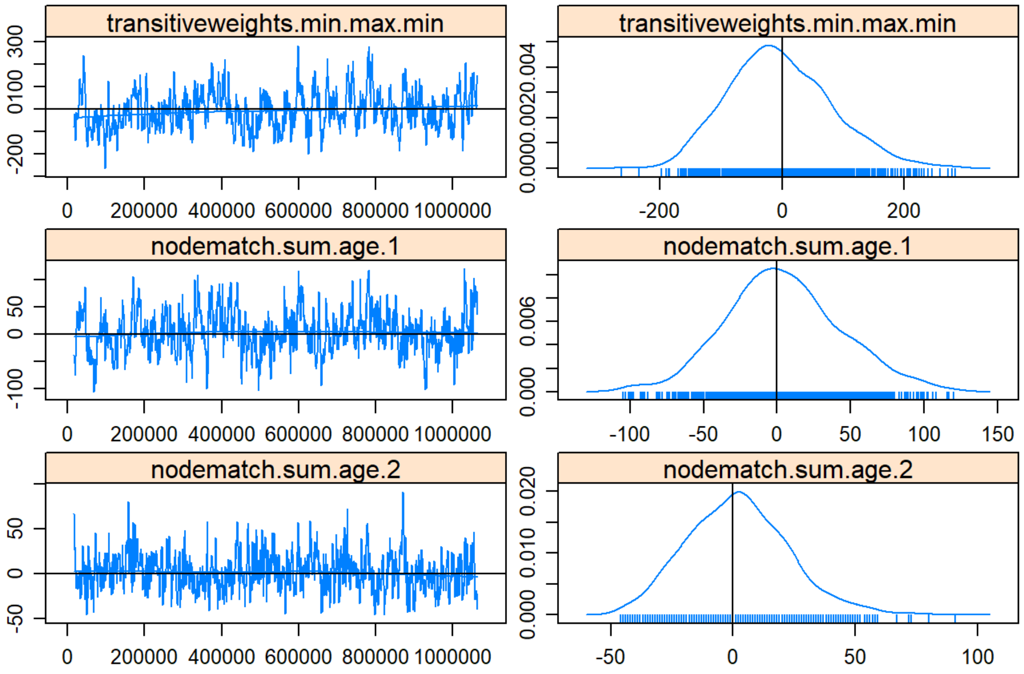

Sample statistics

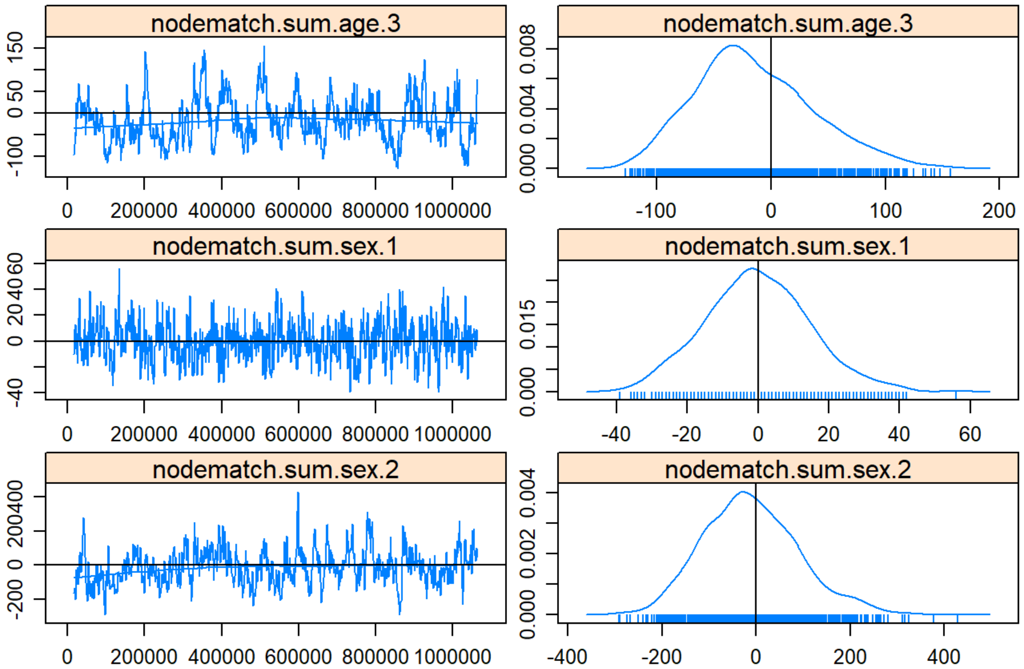

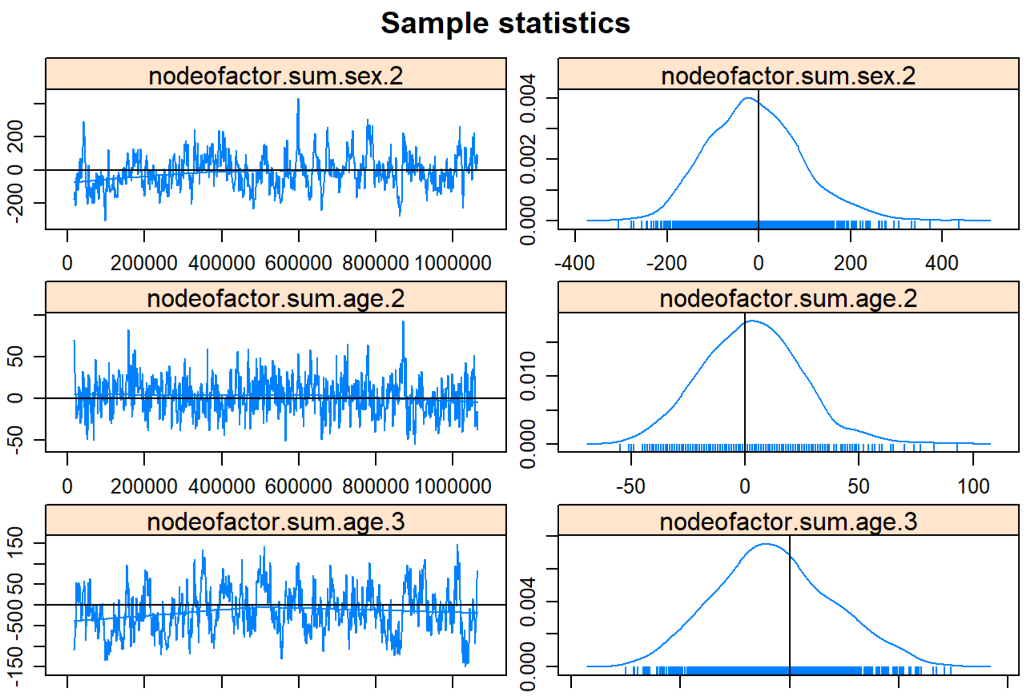


```
##
## MCMC diagnostics shown here are from the last round of simulation, prior to computation of final paramete
r estimates. Because the final estimates are refinements of those used for this simulation run, these diagno
stics may understate model performance. To directly assess the performance of the final model on in-model st
atistics, please use the GOF command: gof(ergmFitObject, GOF=~model).
```

```
mcmc.diagnostics(FDg)
```

```
## Sample statistics summary:
##
## Iterations = 16384:4209664
## Thinning interval = 1024
## Number of chains = 1
## Sample size per chain = 4096
##
## 1. Empirical mean and standard deviation for each variable,
##    plus standard error of the mean:
##
##                                   Mean    SD Naive SE Time-series SE
## sum                           -7.09570 79.73   1.2458         3.7516
## nonzero                        0.37183 10.38   0.1621         0.3301
## cyclicalweights.min.max.min    0.63745 32.75   0.5117         1.6221
## transitiveweights.min.max.min -0.02197 62.73   0.9802         2.8999
## nodematch.sum.age.1            2.65869 41.51   0.6486         1.9733
## nodematch.sum.age.2           -0.66211 11.35   0.1774         0.2972
## nodematch.sum.age.3            3.11328 23.77   0.3715         1.1308
## nodematch.sum.sex.1            1.02588 18.29   0.2857         0.5704
## nodematch.sum.sex.2           -5.62158 64.59   1.0092         3.1064
## nodeofactor.sum.sex.2         -8.36304 76.39   1.1936         3.7810
## nodeofactor.sum.age.2         -0.68384 11.32   0.1769         0.2949
## nodeofactor.sum.age.3          2.07764 34.64   0.5412         1.5447
##
## 2. Quantiles for each variable:
##
##                                  2.5%    25%  50% 75% 97.5%
## sum                           -158.62 -62.00 -7.0  47   152
## nonzero                        -20.00  -7.00  0.0   7    21
## cyclicalweights.min.max.min    -62.00 -22.00  1.0  23    64
## transitiveweights.min.max.min -120.00 -43.00  0.0  41   126
## nodematch.sum.age.1            -72.00 -26.00  0.0  29    93
## nodematch.sum.age.2            -22.00  -8.00 -1.0   7    22
## nodematch.sum.age.3            -38.00 -14.00  1.0  18    54
## nodematch.sum.sex.1            -32.00 -12.00  0.0  13    39
## nodematch.sum.sex.2           -125.62 -51.00 -7.5  38   126
```

```
## nodematch.sum.sex.2           [illegible]
## nodeofactor.sum.sex.2         -151.00 -61.25 -8.0  43   144
## nodeofactor.sum.age.2          -21.00  -9.00 -1.0   7    22
## nodeofactor.sum.age.3          -61.62 -22.00  0.0  24    73
##
##
## Sample statistics cross-correlations:
##                                     sum     nonzero
## sum                           1.0000000 0.350415801
## nonzero                       0.3504158 1.000000000
## cyclicalweights.min.max.min  -0.0137620 0.576600033
## transitiveweights.min.max.min  0.9010340 0.436116632
## nodematch.sum.age.1           0.5776575 0.185667107
## nodematch.sum.age.2           0.1692170 0.252198171
## nodematch.sum.age.3           0.3281884 0.005273639
## nodematch.sum.sex.1           0.2516035 0.216225269
## nodematch.sum.sex.2           0.8095794 0.140472652
## nodeofactor.sum.sex.2         0.9231002 0.225529968
## nodeofactor.sum.age.2         0.1661824 0.250624672
## nodeofactor.sum.age.3         0.3289403 0.172319038
##                              cyclicalweights.min.max.min
## sum                                          -0.01376200
## nonzero                                       0.57660003
## cyclicalweights.min.max.min                   1.00000000
## transitiveweights.min.max.min                 0.06328431
## nodematch.sum.age.1                           0.03893115
## nodematch.sum.age.2                           0.06304759
## nodematch.sum.age.3                          -0.06210161
## nodematch.sum.sex.1                          -0.01037649
## nodematch.sum.sex.2                          -0.04965260
## nodeofactor.sum.sex.2                        -0.07223936
## nodeofactor.sum.age.2                         0.07778626
## nodeofactor.sum.age.3                         0.08399573
##                              transitiveweights.min.max.min
## sum                                             0.90103404
## nonzero                                         0.43611663
## cyclicalweights.min.max.min                     0.06328431
## transitiveweights.min.max.min                   1.00000000
## nodematch.sum.age.1                             0.55198427
## nodematch.sum.age.2                             0.15254049
## nodematch.sum.age.3                             0.26031944
## nodematch.sum.sex.1                             0.22078365
## nodematch.sum.sex.2                             0.72361305
## nodeofactor.sum.sex.2                           0.82631787
## nodeofactor.sum.age.2                           0.14971141
## nodeofactor.sum.age.3                           0.29652986
##                              nodematch.sum.age.1 nodematch.sum.age.2
## sum                                  0.577657472         0.169216995
## nonzero                              0.185667107         0.252198171
## cyclicalweights.min.max.min          0.038931147         0.063047594
## transitiveweights.min.max.min        0.551984272         0.152540487
## nodematch.sum.age.1                  1.000000000         0.008591378
## nodematch.sum.age.2                  0.008591378         1.000000000
## nodematch.sum.age.3                 -0.016376499         0.005317925
## nodematch.sum.sex.1                  0.184642029         0.090360234
## nodematch.sum.sex.2                  0.424964309         0.084979695
## nodeofactor.sum.sex.2                0.563965749         0.137000548
## nodeofactor.sum.age.2                0.010050838         0.995090136
## nodeofactor.sum.age.3               -0.070849222         0.012517495
##                              nodematch.sum.age.3 nodematch.sum.sex.1
## sum                                  0.328188447         0.251603496
## nonzero                              0.005273639         0.216225269
## cyclicalweights.min.max.min         -0.062101614        -0.010376491
## transitiveweights.min.max.min        0.260319439         0.220783652
## nodematch.sum.age.1                 -0.016376499         0.184642029
## nodematch.sum.age.2                  0.005317925         0.090360234
## nodematch.sum.age.3                  1.000000000         0.009663911
## nodematch.sum.sex.1                  0.009663911         1.000000000
## nodematch.sum.sex.2                  0.348686574        -0.011956527
## nodeofactor.sum.sex.2                0.331958199         0.037402599
## nodeofactor.sum.age.2                0.001701810         0.087698156
## nodeofactor.sum.age.3                0.689860206         0.029490360
##                              nodematch.sum.sex.2 nodeofactor.sum.sex.2
[illegible]
```

```
## sum                                      0.80957940           0.92310021
## nonzero                                  0.14047265           0.22552997
## cyclicalweights.min.max.min             -0.04965260          -0.07223936
## transitiveweights.min.max.min            0.72361305           0.82631787
## nodematch.sum.age.1                      0.42496431           0.56396575
## nodematch.sum.age.2                      0.08497969           0.13700055
## nodematch.sum.age.3                      0.34868657           0.33195820
## nodematch.sum.sex.1                     -0.01195653           0.03740260
## nodematch.sum.sex.2                      1.00000000           0.84734438
## nodeofactor.sum.sex.2                    0.84734438           1.00000000
## nodeofactor.sum.age.2                    0.08291563           0.13612615
## nodeofactor.sum.age.3                    0.27991984           0.35691974
##                               nodeofactor.sum.age.2 nodeofactor.sum.age.3
## sum                                     0.166182352           0.328940329
## nonzero                                 0.250624672           0.172319038
## cyclicalweights.min.max.min             0.077786261           0.083995731
## transitiveweights.min.max.min           0.149711405           0.296529859
## nodematch.sum.age.1                     0.010050838          -0.070849222
## nodematch.sum.age.2                     0.995090136           0.012517495
## nodematch.sum.age.3                     0.001701810           0.689860206
## nodematch.sum.sex.1                     0.087698156           0.029490360
## nodematch.sum.sex.2                     0.082915627           0.279919839
## nodeofactor.sum.sex.2                   0.136126152           0.356919742
## nodeofactor.sum.age.2                   1.000000000           0.006745894
## nodeofactor.sum.age.3                   0.006745894           1.000000000
##
## Sample statistics auto-correlation:
## Chain 1
##                sum    nonzero cyclicalweights.min.max.min
## Lag 0    1.0000000 1.00000000                   1.0000000
## Lag 1024 0.7397272 0.44795417                   0.7134590
## Lag 2048 0.5695630 0.24712597                   0.5488558
## Lag 3072 0.4591988 0.16513989                   0.4487442
## Lag 4096 0.3695880 0.12518446                   0.3667407
## Lag 5120 0.3169210 0.09254696                   0.3194949
##          transitiveweights.min.max.min nodematch.sum.age.1
## Lag 0                        1.0000000           1.0000000
## Lag 1024                     0.7048499           0.7617688
## Lag 2048                     0.5442147           0.5907761
## Lag 3072                     0.4379433           0.4659058
## Lag 4096                     0.3649720           0.3768991
## Lag 5120                     0.3104394           0.3217067
##          nodematch.sum.age.2 nodematch.sum.age.3 nodematch.sum.sex.1
## Lag 0            1.000000000           1.0000000           1.0000000
## Lag 1024         0.474547978           0.7662191           0.5987211
## Lag 2048         0.230580303           0.5973048           0.3641656
## Lag 3072         0.124642765           0.4770257           0.2117811
## Lag 4096         0.055560577           0.3896783           0.1228345
## Lag 5120         0.002176102           0.3269928           0.0736775
##          nodematch.sum.sex.2 nodeofactor.sum.sex.2 nodeofactor.sum.age.2
## Lag 0              1.0000000             1.0000000           1.000000000
## Lag 1024           0.7847653             0.7743892           0.470922120
## Lag 2048           0.6278288             0.6210755           0.226879436
## Lag 3072           0.5106446             0.5143057           0.123409141
## Lag 4096           0.4077695             0.4189488           0.055326722
## Lag 5120           0.3373018             0.3548203           0.003443252
##          nodeofactor.sum.age.3
## Lag 0                1.0000000
## Lag 1024             0.7168020
## Lag 2048             0.5287661
## Lag 3072             0.4074419
## Lag 4096             0.3253090
## Lag 5120             0.2699772
##
## Sample statistics burn-in diagnostic (Geweke):
## Chain 1
##
## Fraction in 1st window = 0.1
## Fraction in 2nd window = 0.5
##
##                           sum                       nonzero
##                       -0.7454                       -1.2388
##   cyclicalweights.min.max.min transitiveweights.min.max.min
```

```
##                           -0.1443                         -1.0446
##              nodematch.sum.age.1             nodematch.sum.age.2
##                            0.2744                          0.1752
##              nodematch.sum.age.3             nodematch.sum.sex.1
##                           -0.7864                         -1.0549
##              nodematch.sum.sex.2           nodeofactor.sum.sex.2
##                           -0.6886                         -0.6495
##            nodeofactor.sum.age.2           nodeofactor.sum.age.3
##                            0.1987                          0.1774
##
## Individual P-values (lower = worse):
##                               sum                         nonzero
##                         0.4560058                       0.2154189
##   cyclicalweights.min.max.min transitiveweights.min.max.min
##                         0.8852650                       0.2961887
##              nodematch.sum.age.1             nodematch.sum.age.2
##                         0.7837912                       0.8609081
##              nodematch.sum.age.3             nodematch.sum.sex.1
##                         0.4316396                       0.2914922
##              nodematch.sum.sex.2           nodeofactor.sum.sex.2
##                         0.4911003                       0.5160420
##            nodeofactor.sum.age.2           nodeofactor.sum.age.3
##                         0.8425163                       0.8592179
## Joint P-value (lower = worse):  0.4018895 .
```

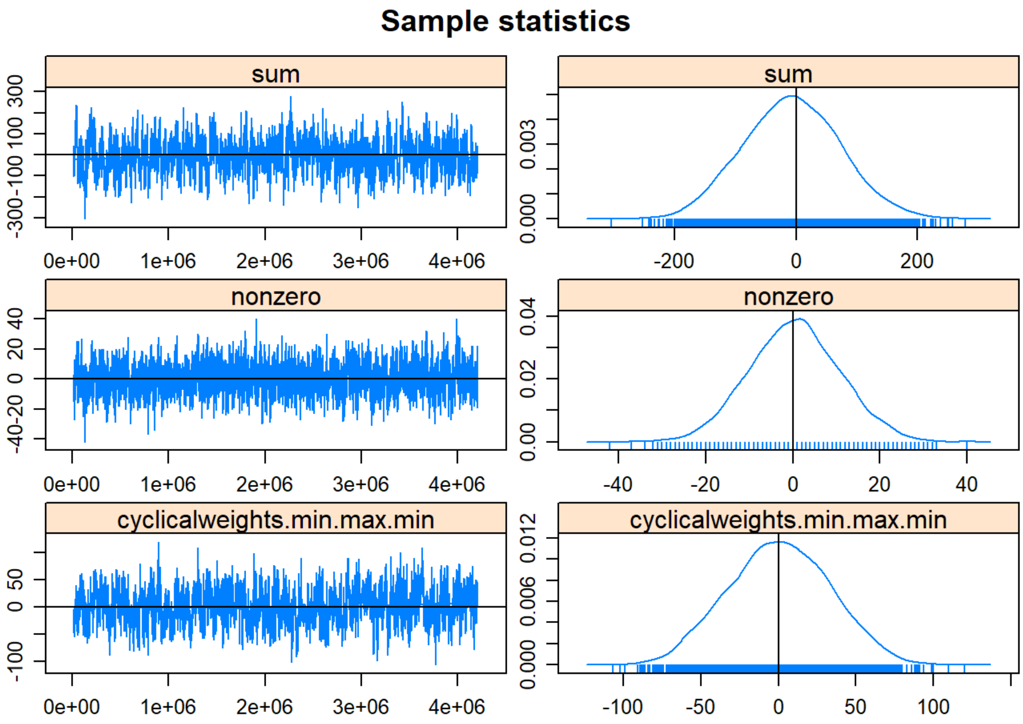

Sample statistics

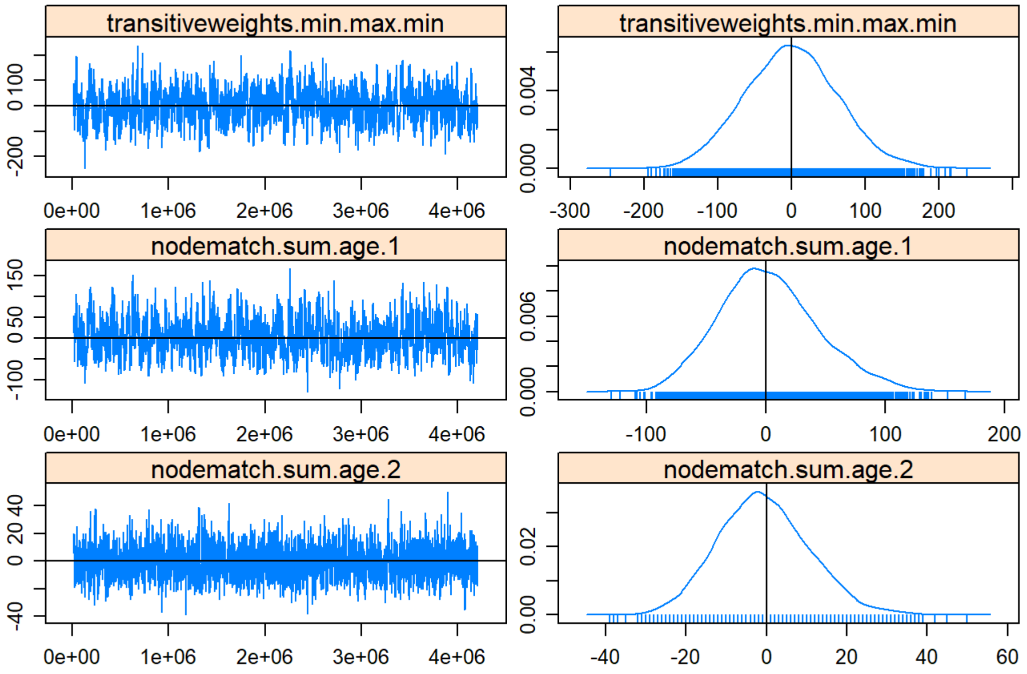

Sample statistics

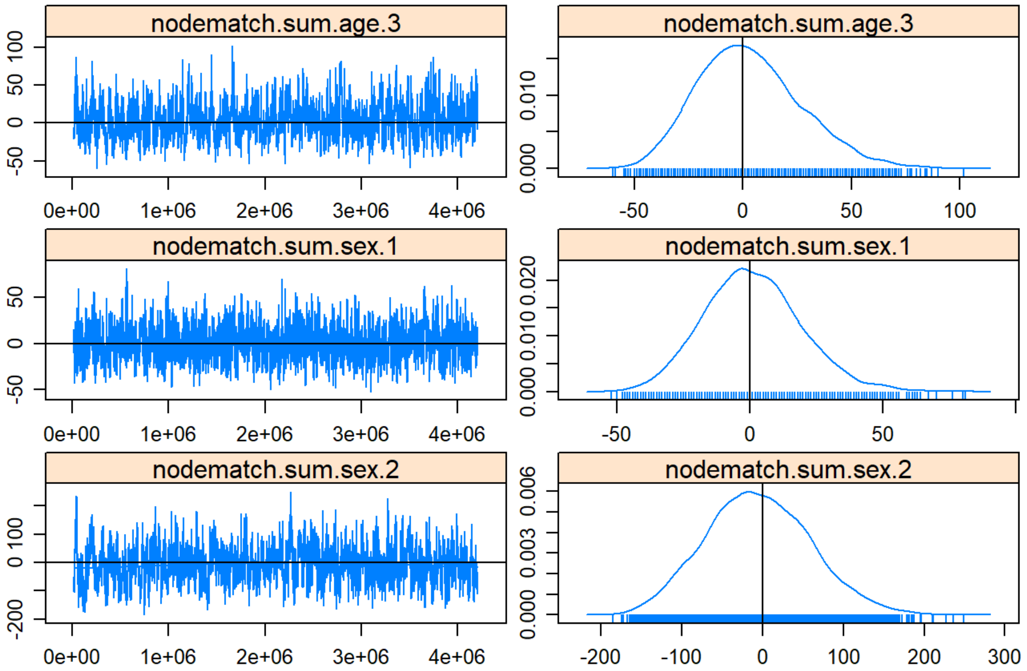

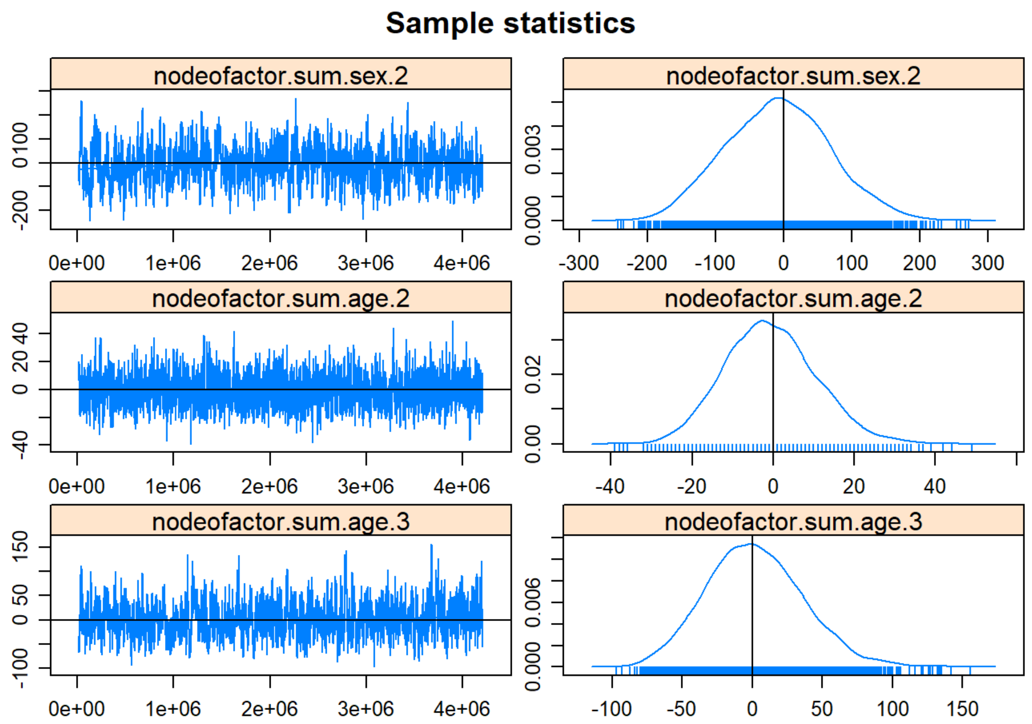


```
##
## MCMC diagnostics shown here are from the last round of simulation, prior to computation of final paramete
r estimates. Because the final estimates are refinements of those used for this simulation run, these diagno
stics may understate model performance. To directly assess the performance of the final model on in-model st
atistics, please use the GOF command: gof(ergmFitObject, GOF=~model).
```

```
mcmc.diagnostics(FSg)
```

```
## Sample statistics summary:
##
## Iterations = 16384:4209664
## Thinning interval = 1024
## Number of chains = 1
## Sample size per chain = 4096
##
## 1. Empirical mean and standard deviation for each variable,
##    plus standard error of the mean:
##
##                                  Mean      SD Naive SE Time-series SE
## sum                            1.1116 175.416   2.7409        10.2397
## nonzero                       -2.1023   9.357   0.1462         0.2783
## cyclicalweights.min.max.min   -0.7297  36.269   0.5667         1.4023
## transitiveweights.min.max.min -8.2334 143.643   2.2444         9.0838
## nodematch.sum.age.1            2.0793  59.891   0.9358         2.6516
## nodematch.sum.age.2           -9.2107  24.712   0.3861         0.8665
## nodematch.sum.age.3          -13.0647  51.449   0.8039         3.3073
## nodematch.sum.sex.1           -3.2537  31.226   0.4879         1.1180
## nodematch.sum.sex.2           13.9446 154.661   2.4166         8.7340
## nodeofactor.sum.sex.2         21.1609 165.776   2.5903         9.8058
## nodeofactor.sum.age.2         -7.3035 114.108   1.7829         7.0224
## nodeofactor.sum.age.3          5.0552 107.347   1.6773         6.8325
##
## 2. Quantiles for each variable:
##
##                                 2.5%  25% 50% 75%  97.5%
## sum                           -340.00 -117  -4 114 360.00
## nonzero                        -20.00   -8  -2   4  16.00
## cyclicalweights.min.max.min    -64.00  -27  -3  22  76.00
## transitiveweights.min.max.min -276.25 -105 -13  81 294.00
## nodematch.sum.age.1           -105.00  -41  -1  40 128.00
## nodematch.sum.age.2            -55.00  -26 -10   7  41.00
## nodematch.sum.age.3            -96.00  -50 -18  18 103.00
## nodematch.sum.sex.1            -59.62  -25  -5  17  61.62
## nodematch.sum.sex.2           -270.62  -94   5 116 336.62
```

```
[illegible]
## nodeofactor.sum.sex.2         -284.00  -95  14 131 357.62
## nodeofactor.sum.age.2         -221.62  -84 -10  68 223.00
## nodeofactor.sum.age.3         -191.62  -71   3  76 220.62
##
##
## Sample statistics cross-correlations:
##                                      sum    nonzero
## sum                           1.00000000 0.32266363
## nonzero                       0.32266363 1.00000000
## cyclicalweights.min.max.min  -0.05090698 0.45694590
## transitiveweights.min.max.min  0.92558297 0.37610724
## nodematch.sum.age.1           0.42338384 0.12534652
## nodematch.sum.age.2           0.19308733 0.22683278
## nodematch.sum.age.3           0.42183896 0.03069231
## nodematch.sum.sex.1           0.23708387 0.19921069
## nodematch.sum.sex.2           0.91297960 0.18799018
## nodeofactor.sum.sex.2         0.92436100 0.23562499
## nodeofactor.sum.age.2         0.71412477 0.28857420
## nodeofactor.sum.age.3         0.64573880 0.14808177
##                              cyclicalweights.min.max.min
## sum                                        -0.050906981
## nonzero                                     0.456945900
## cyclicalweights.min.max.min                 1.000000000
## transitiveweights.min.max.min              -0.038435204
## nodematch.sum.age.1                        -0.013782960
## nodematch.sum.age.2                        -0.007310408
## nodematch.sum.age.3                        -0.048274847
## nodematch.sum.sex.1                         0.014864805
## nodematch.sum.sex.2                        -0.017762988
## nodeofactor.sum.sex.2                      -0.045565129
## nodeofactor.sum.age.2                      -0.050909933
## nodeofactor.sum.age.3                      -0.048940991
##                              transitiveweights.min.max.min
## sum                                              0.9255830
## nonzero                                          0.3761072
## cyclicalweights.min.max.min                     -0.0384352
## transitiveweights.min.max.min                    1.0000000
## nodematch.sum.age.1                              0.3847728
## nodematch.sum.age.2                              0.2005729
## nodematch.sum.age.3                              0.4105414
## nodematch.sum.sex.1                              0.1981788
## nodematch.sum.sex.2                              0.8408121
## nodeofactor.sum.sex.2                            0.8547059
## nodeofactor.sum.age.2                            0.6670279
## nodeofactor.sum.age.3                            0.5975429
##                              nodematch.sum.age.1 nodematch.sum.age.2
## sum                                  0.423383845         0.193087335
## nonzero                              0.125346519         0.226832776
## cyclicalweights.min.max.min         -0.013782960        -0.007310408
## transitiveweights.min.max.min        0.384772754         0.200572934
## nodematch.sum.age.1                  1.000000000         0.009989435
## nodematch.sum.age.2                  0.009989435         1.000000000
## nodematch.sum.age.3                 -0.053818822         0.053304028
## nodematch.sum.sex.1                  0.101291631         0.090360948
## nodematch.sum.sex.2                  0.347407917         0.137454094
## nodeofactor.sum.sex.2                0.377789718         0.137763692
## nodeofactor.sum.age.2                0.115876636         0.248216558
## nodeofactor.sum.age.3                0.012011957         0.045769781
##                              nodematch.sum.age.3 nodematch.sum.sex.1
## sum                                   0.42183896          0.23708387
## nonzero                               0.03069231          0.19921069
## cyclicalweights.min.max.min          -0.04827485          0.01486480
## transitiveweights.min.max.min         0.41054143          0.19817880
## nodematch.sum.age.1                  -0.05381882          0.10129163
## nodematch.sum.age.2                   0.05330403          0.09036095
## nodematch.sum.age.3                   1.00000000          0.06695321
## nodematch.sum.sex.1                   0.06695321          1.00000000
## nodematch.sum.sex.2                   0.42759362          0.05832062
## nodeofactor.sum.sex.2                 0.39149597          0.05999484
## nodeofactor.sum.age.2                 0.12865395          0.22347954
## nodeofactor.sum.age.3                 0.58292194          0.09783078
##                              nodematch.sum.sex.2 nodeofactor.sum.sex.2
[illegible]
```

```
## sum                                    0.91297960           0.92436100
## nonzero                                0.18799018           0.23562499
## cyclicalweights.min.max.min           -0.01776299          -0.04556513
## transitiveweights.min.max.min          0.84081214           0.85470587
## nodematch.sum.age.1                    0.34740792           0.37778972
## nodematch.sum.age.2                    0.13745409           0.13776369
## nodematch.sum.age.3                    0.42759362           0.39149597
## nodematch.sum.sex.1                    0.05832062           0.05999484
## nodematch.sum.sex.2                    1.00000000           0.94071122
## nodeofactor.sum.sex.2                  0.94071122           1.00000000
## nodeofactor.sum.age.2                  0.62460685           0.63397015
## nodeofactor.sum.age.3                  0.63623409           0.63523429
##                               nodeofactor.sum.age.2 nodeofactor.sum.age.3
## sum                                      0.71412477            0.64573880
## nonzero                                  0.28857420            0.14808177
## cyclicalweights.min.max.min             -0.05090993           -0.04894099
## transitiveweights.min.max.min            0.66702795            0.59754289
## nodematch.sum.age.1                      0.11587664            0.01201196
## nodematch.sum.age.2                      0.24821656            0.04576978
## nodematch.sum.age.3                      0.12865395            0.58292194
## nodematch.sum.sex.1                      0.22347954            0.09783078
## nodematch.sum.sex.2                      0.62460685            0.63623409
## nodeofactor.sum.sex.2                    0.63397015            0.63523429
## nodeofactor.sum.age.2                    1.00000000            0.05118255
## nodeofactor.sum.age.3                    0.05118255            1.00000000
##
## Sample statistics auto-correlation:
## Chain 1
##                 sum    nonzero cyclicalweights.min.max.min
## Lag 0    1.0000000 1.00000000                   1.0000000
## Lag 1024 0.8376469 0.47683436                   0.7006666
## Lag 2048 0.7150325 0.27475553                   0.5098650
## Lag 3072 0.6152855 0.18061446                   0.3740407
## Lag 4096 0.5408875 0.13042269                   0.2817193
## Lag 5120 0.4760713 0.08332317                   0.2218014
##          transitiveweights.min.max.min nodematch.sum.age.1
## Lag 0                        1.0000000           1.0000000
## Lag 1024                     0.8504756           0.7784332
## Lag 2048                     0.7438787           0.6094519
## Lag 3072                     0.6582835           0.4883593
## Lag 4096                     0.5894299           0.3994163
## Lag 5120                     0.5208399           0.3260192
##          nodematch.sum.age.2 nodematch.sum.age.3 nodematch.sum.sex.1
## Lag 0              1.0000000           1.0000000           1.0000000
## Lag 1024           0.6445848           0.8518110           0.6799726
## Lag 2048           0.4400536           0.7386438           0.4708903
## Lag 3072           0.3076681           0.6497811           0.3190977
## Lag 4096           0.2236784           0.5745384           0.2128472
## Lag 5120           0.1555649           0.5212535           0.1479736
##          nodematch.sum.sex.2 nodeofactor.sum.sex.2 nodeofactor.sum.age.2
## Lag 0              1.0000000             1.0000000             1.0000000
## Lag 1024           0.8485745             0.8497946             0.8233145
## Lag 2048           0.7294779             0.7356664             0.6962182
## Lag 3072           0.6305272             0.6435069             0.6014380
## Lag 4096           0.5477555             0.5660988             0.5311895
## Lag 5120           0.4797174             0.5009546             0.4763349
##          nodeofactor.sum.age.3
## Lag 0                1.0000000
## Lag 1024             0.8462092
## Lag 2048             0.7348217
## Lag 3072             0.6519226
## Lag 4096             0.5857714
## Lag 5120             0.5314350
##
## Sample statistics burn-in diagnostic (Geweke):
## Chain 1
##
## Fraction in 1st window = 0.1
## Fraction in 2nd window = 0.5
##
##                           sum                       nonzero
##                      -0.69407                      -0.70192
##   cyclicalweights.min.max.min transitiveweights.min.max.min
```

```
##                          -1.31608                         -0.64230
##               nodematch.sum.age.1              nodematch.sum.age.2
##                           0.10303                         -0.13865
##               nodematch.sum.age.3              nodematch.sum.sex.1
##                          -2.20094                          0.29065
##               nodematch.sum.sex.2            nodeofactor.sum.sex.2
##                          -0.88687                          0.07874
##             nodeofactor.sum.age.2            nodeofactor.sum.age.3
##                           0.05911                         -1.06765
##
## Individual P-values (lower = worse):
##                               sum                          nonzero
##                        0.48763767                       0.48272933
##    cyclicalweights.min.max.min transitiveweights.min.max.min
##                        0.18814777                       0.52068126
##               nodematch.sum.age.1              nodematch.sum.age.2
##                        0.91793856                       0.88972657
##               nodematch.sum.age.3              nodematch.sum.sex.1
##                        0.02773992                       0.77131871
##               nodematch.sum.sex.2            nodeofactor.sum.sex.2
##                        0.37514942                       0.93723928
##             nodeofactor.sum.age.2            nodeofactor.sum.age.3
##                        0.95286736                       0.28567652
## Joint P-value (lower = worse):  0.151106 .
```

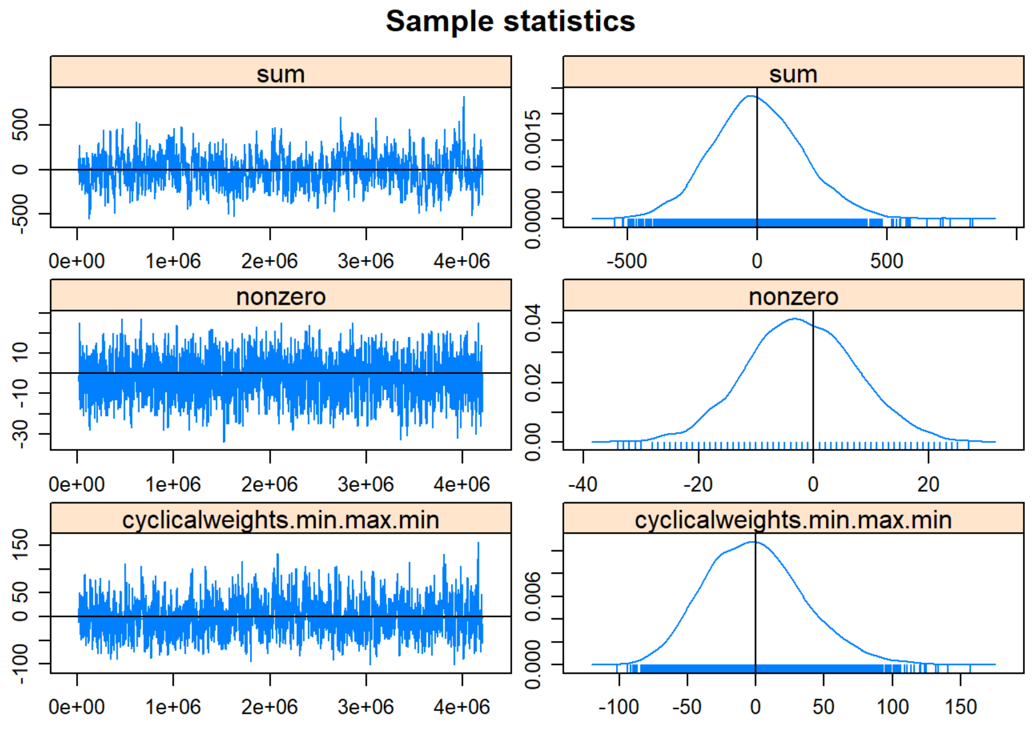

Sample statistics

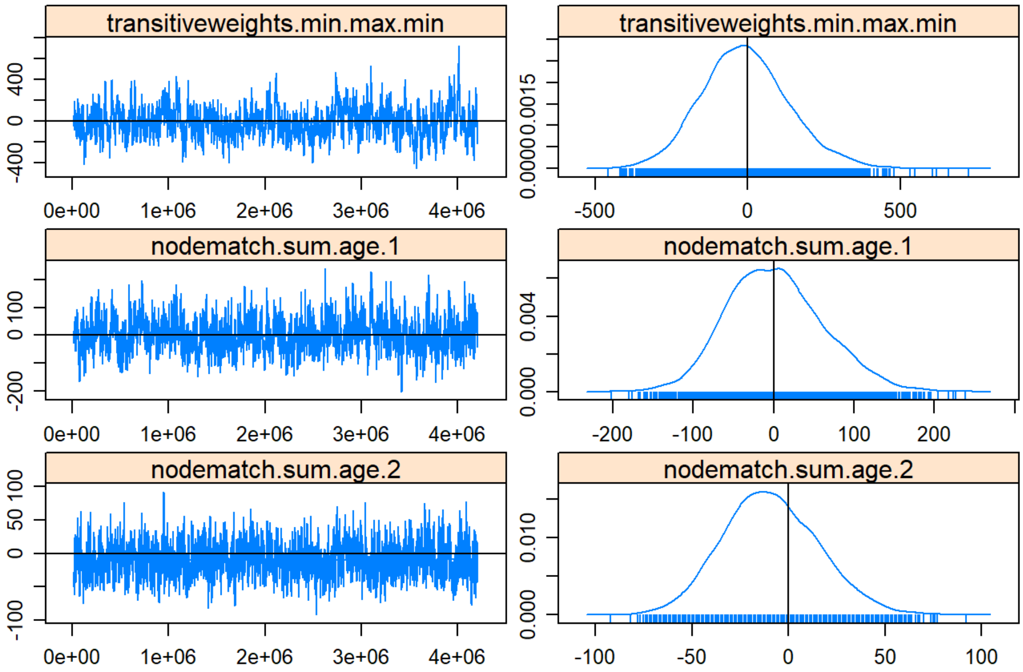

Sample statistics

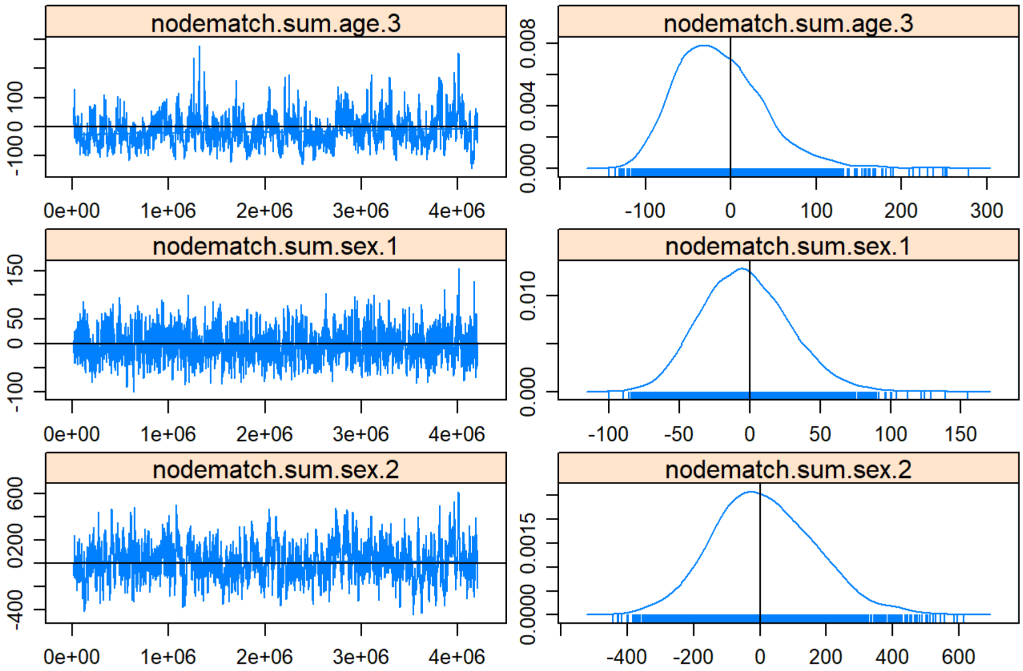

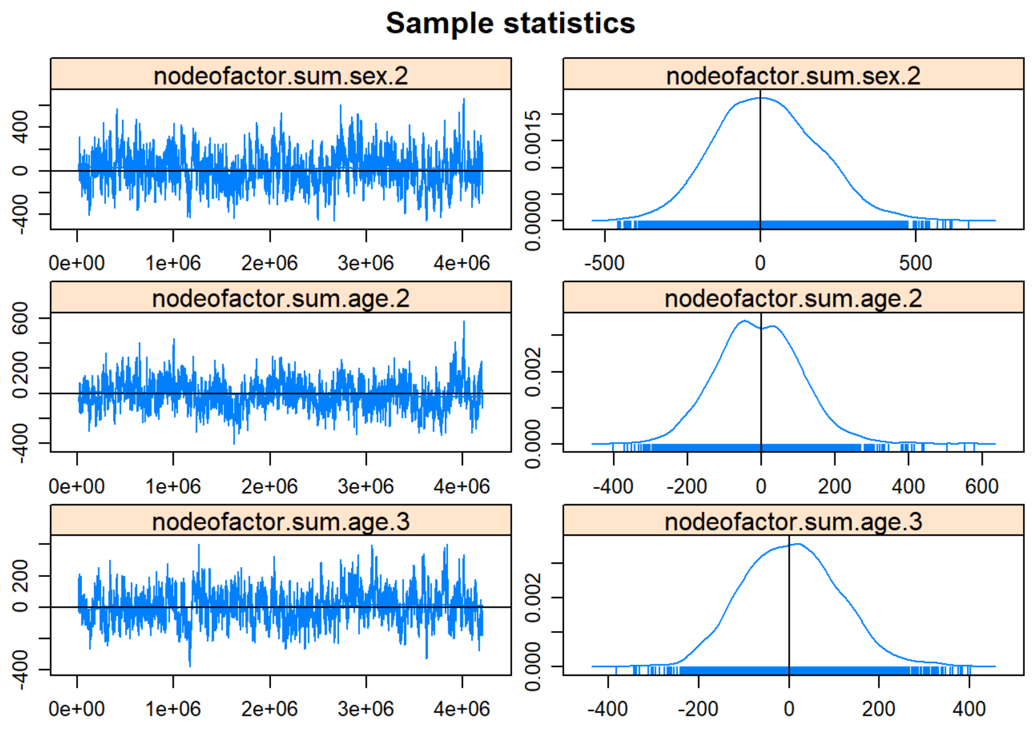


```
##
## MCMC diagnostics shown here are from the last round of simulation, prior to computation of final paramete
r estimates. Because the final estimates are refinements of those used for this simulation run, these diagno
stics may understate model performance. To directly assess the performance of the final model on in-model st
atistics, please use the GOF command: gof(ergmFitObject, GOF=~model).
```

```
summary(FDg)
```

```
## Note: Null model likelihood calculation is not implemented for valued ERGMs at this time.
```

```
##
## ==========================
## Summary of model fit
## ==========================
##
## Formula:   domint ~ sum + nonzero + cyclicalweights(twopath = "min", combine = "max",
##     affect = "min") + transitiveweights(twopath = "min", combine = "max",
##     affect = "min") + nodematch("age", diff = TRUE) + nodematch("sex",
##     diff = TRUE) + nodeofactor("sex") + nodeofactor("age")
##
## Iterations:  3 out of 20
##
## Monte Carlo MLE Results:
##                                 Estimate Std. Error MCMC %  p-value
## sum                            -0.449262   0.050523      0  < 1e-04 ***
## nonzero                        -0.341594   0.148123      0 0.021398 *
## cyclicalweights.min.max.min   -0.350597   0.041218      0  < 1e-04 ***
## transitiveweights.min.max.min  0.133303   0.038966      0 0.000661 ***
## nodematch.sum.age.1             0.035566   0.033461      0 0.288196
## nodematch.sum.age.2             1.625771   0.914238      0 0.075798 .
## nodematch.sum.age.3             0.201064   0.061825      0 0.001201 **
## nodematch.sum.sex.1             0.003357   0.068874      0 0.961143
## nodematch.sum.sex.2             0.087235   0.031660      0 0.006018 **
## nodeofactor.sum.sex.2           0.118296   0.045875      0 0.010124 *
## nodeofactor.sum.age.2          -2.033172   0.915970      0 0.026764 *
## nodeofactor.sum.age.3          -0.142322   0.044856      0 0.001576 **
## ---
## Signif. codes:  0 '***' 0.001 '**' 0.01 '*' 0.05 '.' 0.1 ' ' 1
##
##      Null Deviance:     0  on 702  degrees of freedom
##  Residual Deviance: -5481  on 690  degrees of freedom
##
## Note that the null model likelihood and deviance are defined to be 0.
##
## AIC: -5457    BIC: -5402    (Smaller is better.)
```

```
summary(FAg)
```

```
## Note: Null model likelihood calculation is not implemented for valued ERGMs at this time.
```

```
##
## ==========================
## Summary of model fit
## ==========================
##
## Formula:   agint ~ sum + nonzero + cyclicalweights(twopath = "min", combine = "max",
##     affect = "min") + transitiveweights(twopath = "min", combine = "max",
##     affect = "min") + nodematch("age", diff = TRUE) + nodematch("sex",
##     diff = TRUE) + nodeofactor("sex") + nodeofactor("age")
##
## Iterations:  20 out of 20
##
## Monte Carlo MLE Results:
##                                Estimate Std. Error MCMC %  p-value
## sum                           -0.524255   0.063129      0  < 1e-04 ***
## nonzero                       -1.061722   0.138815      0  < 1e-04 ***
## cyclicalweights.min.max.min   -0.113264   0.027572      0  < 1e-04 ***
## transitiveweights.min.max.min  0.123122   0.034218      0 0.000343 ***
## nodematch.sum.age.1           -0.002295   0.035276      0 0.948142
## nodematch.sum.age.2            0.688246   0.170257      0  < 1e-04 ***
## nodematch.sum.age.3            0.203340   0.042909      0  < 1e-04 ***
## nodematch.sum.sex.1            0.028053   0.088392      0 0.751059
## nodematch.sum.sex.2            0.510204   0.083167      0  < 1e-04 ***
## nodeofactor.sum.sex.2         -0.171618   0.087710      0 0.050792 .
## nodeofactor.sum.age.2         -0.815976   0.170205      0  < 1e-04 ***
## nodeofactor.sum.age.3         -0.124674   0.044546      0 0.005273 **
## ---
## Signif. codes:  0 '***' 0.001 '**' 0.01 '*' 0.05 '.' 0.1 ' ' 1
##
##      Null Deviance:     0  on 702  degrees of freedom
##  Residual Deviance: -5955  on 690  degrees of freedom
##
## Note that the null model likelihood and deviance are defined to be 0.
##
## AIC: -5931    BIC: -5877    (Smaller is better.)
```

```
summary(FSg)
```

```
## Note: Null model likelihood calculation is not implemented for valued ERGMs at this time.
```

```
##
## ==========================
## Summary of model fit
## ==========================
##
## Formula:   subint ~ sum + nonzero + cyclicalweights(twopath = "min", combine = "max",
##     affect = "min") + transitiveweights(twopath = "min", combine = "max",
##     affect = "min") + nodematch("age", diff = TRUE) + nodematch("sex",
##     diff = TRUE) + nodeofactor("sex") + nodeofactor("age")
##
## Iterations:  5 out of 20
##
## Monte Carlo MLE Results:
##                              Estimate Std. Error MCMC %  p-value
## sum                          -0.47441    0.11602      0  < 1e-04 ***
## nonzero                      -0.65805    0.14808      0  < 1e-04 ***
## cyclicalweights.min.max.min  -0.35799    0.03497      0  < 1e-04 ***
## transitiveweights.min.max.min  0.07351    0.01916      0 0.000136 ***
## nodematch.sum.age.1           0.24209    0.11731      0 0.039428 *
## nodematch.sum.age.2          -0.12885    0.04276      0 0.002679 **
## nodematch.sum.age.3           0.04302    0.02467      0 0.081627 .
## nodematch.sum.sex.1          -0.02622    0.03745      0 0.483977
## nodematch.sum.sex.2           0.13491    0.02225      0  < 1e-04 ***
## nodeofactor.sum.sex.2        -0.02628    0.02159      0 0.223939
## nodeofactor.sum.age.2         0.24040    0.11490      0 0.036785 *
## nodeofactor.sum.age.3         0.23714    0.11553      0 0.040497 *
## ---
## Signif. codes:  0 '***' 0.001 '**' 0.01 '*' 0.05 '.' 0.1 ' ' 1
##
##      Null Deviance:     0  on 702  degrees of freedom
##  Residual Deviance: -5824  on 690  degrees of freedom
##
## Note that the null model likelihood and deviance are defined to be 0.
##
## AIC: -5800    BIC: -5745    (Smaller is better.)
```

Now to run rank-based models to test variation in stability of dominance relationships with rank

The initial code includes modelling, simulating networks and calculating heterogeneity in goodness of fit from networks of submissive interactions but these are not plotted in the paper because submissive interactions were used to calculate the rank data in the first place

```
FAg_R<-ergm(agint~sum+nonzero+cyclicalweights(twopath="min",combine="max",affect="min")+transitiveweights(twopath="min",combine="max",affect="min")+mutual(form="nabsdiff")+nodeocov("rank")+nodeicov("rank")+absdiff("rank"),reference=~Geometric,response="weight",silent=TRUE)
FDg_R<-ergm(domint~sum+nonzero+cyclicalweights(twopath="min",combine="max",affect="min")+transitiveweights(twopath="min",combine="max",affect="min")+mutual(form="nabsdiff")+nodeocov("rank")+nodeicov("rank")+absdiff("rank"),reference=~Geometric,response="weight",silent=TRUE)
FSg_R<-ergm(subint~sum+nonzero+cyclicalweights(twopath="min",combine="max",affect="min")+transitiveweights(twopath="min",combine="max",affect="min")+mutual(form="nabsdiff")+nodeocov("rank")+nodeicov("rank")+absdiff("rank"),reference=~Geometric,response="weight",silent=TRUE)
```

and use these models to simulate networks

```
Agnets<-simulate(FAg_R,nsim=1000,response="weight",reference=~Geometric)
Domnets<-simulate(FDg_R,nsim=1000,response="weight",reference=~Geometric)
Simnets<-simulate(FSg_R,nsim=1000,response="weight",reference=~Geometric)
```

This code will calculate the comparison to the observed outcomes and plot Figure 2

It compares the modelled outcomes with observed outcomes as per the methods of the paper and then plots heterogeneity in model fit for the whole hierarchy

```
AG.RES<-array(0,dim=c(27,27,1000))

for ( i in 1:1000){

   AG.RES[,,i]<-Agnets[[i]][,]
   tmp.el<-as.matrix(Agnets[[i]],matrix.type="edgelist")
   tmp.e.att<-network::get.edge.attribute(Agnets[[i]],"weight")
```

```
   for (j in 1:length(tmp.el[,1])){

      AG.RES[tmp.el[j,1],tmp.el[j,2],i]<-tmp.e.att[j]*AG.RES[tmp.el[j,1],tmp.el[j,2],i]

   }

}

DOM.RES<-array(0,dim=c(27,27,1000))

for ( i in 1:1000){

   DOM.RES[,,i]<-Domnets[[i]][,]
   tmp.el<-as.matrix(Domnets[[i]],matrix.type="edgelist")
   tmp.e.att<-network::get.edge.attribute(Domnets[[i]],"weight")

   for (j in 1:length(tmp.el[,1])){

      DOM.RES[tmp.el[j,1],tmp.el[j,2],i]<-tmp.e.att[j]*DOM.RES[tmp.el[j,1],tmp.el[j,2],i]

   }

}

SUB.RES<-array(0,dim=c(27,27,1000))

for ( i in 1:1000){

   SUB.RES[,,i]<-Simnets[[i]][,]
   tmp.el<-as.matrix(Simnets[[i]],matrix.type="edgelist")
   tmp.e.att<-network::get.edge.attribute(Simnets[[i]],"weight")

   for (j in 1:length(tmp.el[,1])){

      SUB.RES[tmp.el[j,1],tmp.el[j,2],i]<-tmp.e.att[j]*SUB.RES[tmp.el[j,1],tmp.el[j,2],i]

   }

}



PR.AG.RES<-array(NA,dim=c(27,27,1000))
PR.DOM.RES<-array(NA,dim=c(27,27,1000))
PR.SUB.RES<-array(NA,dim=c(27,27,1000))

for ( i in 1:1000){

   for (j in 1:27){
      for (k in 1:27){

         if (AG.RES[j,k,i]>0|AG.RES[k,j,i]>0){
            PR.AG.RES[j,k,i]<-AG.RES[j,k,i]/(sum(AG.RES[j,k,i]+AG.RES[k,j,i]))
         }
         else{
            PR.AG.RES[j,k,i]<-NA
         }

      }
   }

}

for ( i in 1:1000){

   for (j in 1:27){
      for (k in 1:27){

         if (DOM.RES[j,k,i]>0|DOM.RES[k,j,i]>0){
            PR.DOM.RES[j,k,i]<-DOM.RES[j,k,i]/(sum(DOM.RES[j,k,i]+DOM.RES[k,j,i]))
         }
         else{
```

```
            PR.DOM.RES[j,k,i]<-NA
         }

      }
   }

}

for ( i in 1:1000){

   for (j in 1:27){
      for (k in 1:27){

         if (SUB.RES[j,k,i]>0|SUB.RES[k,j,i]>0){
            PR.SUB.RES[j,k,i]<-SUB.RES[j,k,i]/(sum(SUB.RES[j,k,i]+SUB.RES[k,j,i]))
         }
         else{
            PR.SUB.RES[j,k,i]<-NA
         }

      }
   }

}

propag2<-matrix(0,nr=27,nc=27)
propdom2<-matrix(0,nr=27,nc=27)
propsub2<-matrix(0,nr=27,nc=27)

for (i in 1:27){
   for (j in 1:27){

      if ( propag[i,j]>0|propag[j,i]>0 ){
         propag2[i,j]<-propag[i,j]
      }
      else{
         propag2[i,j]<-NA
      }
      if ( propdom[i,j]>0|propdom[j,i]>0 ){
         propdom2[i,j]<-propdom[i,j]
      }
      else{
         propdom2[i,j]<-NA
      }
      if ( propsub[i,j]>0|propsub[j,i]>0 ){
         propsub2[i,j]<-propsub[i,j]
      }
      else{
         propsub2[i,j]<-NA
      }

   }
}


diffsag<-array(NA,dim=c(27,27,1000))
diffsdom<-array(NA,dim=c(27,27,1000))
diffssub<-array(NA,dim=c(27,27,1000))

for(i in 1:1000){
for(j in 1:27){
for(k in 1:27){

if(is.na(PR.AG.RES[j,k,i])==FALSE&is.na(propag2[j,k])==FALSE){
diffsag[j,k,i]<-PR.AG.RES[j,k,i]-propag2[j,k]
}
if(is.na(PR.DOM.RES[j,k,i])==FALSE&is.na(propdom2[j,k])==FALSE){
diffsdom[j,k,i]<-PR.DOM.RES[j,k,i]-propdom2[j,k]
}
if(is.na(PR.SUB.RES[j,k,i])==FALSE&is.na(propsub2[j,k])==FALSE){
diffssub[j,k,i]<-PR.SUB.RES[j,k,i]-propsub2[j,k]
}
```

```
}
}
}

med.diffs.ag<-matrix(0,nr=27,nc=27)
med.diffs.dom<-matrix(0,nr=27,nc=27)
med.diffs.sub<-matrix(0,nr=27,nc=27)

medna<-function(a){
median(a,na.rm=TRUE)
}

med.diffs.ag<-apply(diffsag,1:2,medna)
med.diffs.dom<-apply(diffsdom,1:2,medna)
med.diffs.sub<-apply(diffssub,1:2,medna)

#---------------------------------------------


Savsag3<-med.diffs.ag
Savsdom3<-med.diffs.dom
Savssub3<-med.diffs.sub

Savsag3<-t(Savsag3)[,nrow(Savsag3):1]
Savsdom3<-t(Savsdom3)[,nrow(Savsdom3):1]
Savssub3<-t(Savssub3)[,nrow(Savssub3):1]


#Node 1 ID
N1.ID<-rep(1:27,each=27)
#Node 2 ID
N2.ID<-rep(1:27,27)
#Ranking difference
N.DIFF<-N1.ID-N2.ID
#N1 distance from centre
N1.CENT<-abs(N1.ID-median(seq(1:27)))
#N2 distance from centre
N2.CENT<-abs(N2.ID-median(seq(1:27)))

SforMOD<-data.frame(N1.ID,N2.ID,N1.CENT,N2.CENT,N.DIFF)

SAgPs<-rep(0,length(N1.ID))
SDomPs<-rep(0,length(N1.ID))
SSubPs<-rep(0,length(N1.ID))

for ( i in 1:length(SAgPs) ){
   SAgPs[i]<-Savsag3[N1.ID[i],N2.ID[i]]
   SDomPs[i]<-Savsdom3[N1.ID[i],N2.ID[i]]
   SSubPs[i]<-Savssub3[N1.ID[i],N2.ID[i]]
}

SforMOD<-data.frame(SforMOD,SAgPs,SDomPs,SSubPs)

Sagmod2<-as.image(Z=SforMOD$SAgPs,ind=SforMOD[,1:2])
dx<- Sagmod2$x[2]- Sagmod2$x[1]
dy<-  Sagmod2$y[2] - Sagmod2$y[1]
Sagmod3<-image.smooth(Sagmod2$z, dx=dx, dy=dy, theta= 0.25, xwidth=0*dx,ywidth=0*dy)
Sdommod2<-as.image(Z=SforMOD$SDomPs,ind=SforMOD[,1:2])
dx<- Sdommod2$x[2]- Sdommod2$x[1]
dy<-  Sdommod2$y[2] - Sdommod2$y[1]
Sdommod3<-image.smooth(Sdommod2$z, dx=dx, dy=dy, theta= 0.25, xwidth=0*dx,ywidth=0*dy)

fade<-colorRampPalette(c("blue","white","red"), space = "rgb")

par(mfrow=c(1,2))

image(Sdommod3,col=fade(200),las=1,main="Ritualised Dominance Behaviour",xaxt="n",yaxt="n",xlab="Rank",ylab=
"Rank",cex.lab=1.5,cex.main=2)
axis(side=1,at=seq(5,25,5),labels=seq(5,25,5))
axis(side=2,at=seq(2,22,5),labels=rev(seq(5,25,5)),las=1)
lines(c(0.2063492,0.2063492),c(0,27))
lines(c(0,27),c(26.4126984+0.2063492,26.4126984+0.2063492))
par(xpd=NA)
```

```
par(xpd=NA)
text(-0.4,28,"b)",cex=2)
par(xpd=FALSE)
image(Sagmod3,col=fade(200),las=1,main="Aggressive Interactions",xaxt="n",yaxt="n",xlab="Rank",ylab="Rank",c
ex.lab=1.5,cex.main=2)
axis(side=1,at=seq(5,25,5),labels=seq(5,25,5))
axis(side=2,at=seq(2,22,5),labels=rev(seq(5,25,5)),las=1)
lines(c(0.2063492,0.2063492),c(0,27))
lines(c(0,27),c(26.4126984+0.2063492,26.4126984+0.2063492))
par(xpd=NA)
text(-0.4,28,"c)",cex=2)
```

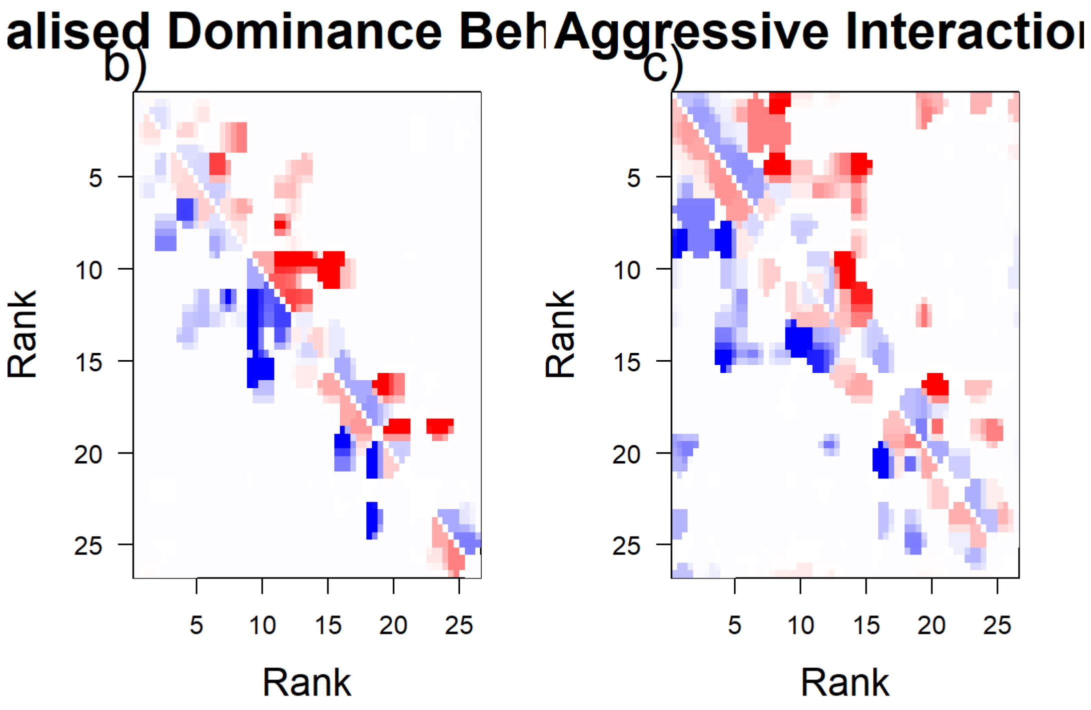

Now for undirected weighted networks to model

1. Create undirected networks
2. Model undirected networks

```
data2<-matrix(0,nr=27,nc=27)
rownames(data2)<-colnames(data2)<-colnames(data)

for(i in 1:26){
   for(j in (i+1):27){
      data2[i,j]<-data2[j,i]<-dom[i,j]+dom[j,i]
   }
}


domint.edgelist<-as.tnet(data2)
domint2<-network(domint.edgelist[,1:2],directed=FALSE)
network::set.edge.attribute(domint2,"weight",as.vector(domint.edgelist[,3]))

network::set.vertex.attribute(domint2,"rank",as.vector(attributes$Rank))
network::set.vertex.attribute(domint2,"ranksq",as.vector(ranksquared))
network::set.vertex.attribute(domint2,"rankce",as.vector(centrank))
network::set.vertex.attribute(domint2,"sex",as.numeric(attributes$Sex))
network::set.vertex.attribute(domint2,"age",as.numeric(attributes$AgeClass))
network::set.vertex.attribute(domint2,"rankcecat",as.vector(centrankcat))
network::set.vertex.attribute(domint2,"rankcecat2",as.vector(centrankcat2))


data2<-matrix(0,nr=27,nc=27)
rownames(data2)<-colnames(data2)<-colnames(data)
```

```
for(i in 1:26){
   for(j in (i+1):27){
      data2[i,j]<-data2[j,i]<-ag[i,j]+ag[j,i]
   }
}

agint.edgelist<-as.tnet(data2)
agint2<-network(agint.edgelist[,1:2],directed=FALSE)
network::set.edge.attribute(agint2,"weight",as.vector(agint.edgelist[,3]))

network::set.vertex.attribute(agint2,"rank",as.vector(attributes$Rank))
network::set.vertex.attribute(agint2,"ranksq",as.vector(ranksquared))
network::set.vertex.attribute(agint2,"rankce",as.vector(centrank))
network::set.vertex.attribute(agint2,"sex",as.numeric(attributes$Sex))
network::set.vertex.attribute(agint2,"age",as.numeric(attributes$AgeClass))
network::set.vertex.attribute(agint2,"age2",as.numeric(as.numeric(attributes$AgeClass)>1)+1)
network::set.vertex.attribute(agint2,"rankcecat",as.vector(centrankcat))
network::set.vertex.attribute(agint2,"rankcecat2",as.vector(centrankcat2))


data2<-matrix(0,nr=27,nc=27)
rownames(data2)<-colnames(data2)<-colnames(data)

for(i in 1:26){
   for(j in (i+1):27){
      data2[i,j]<-data2[j,i]<-sub[i,j]+sub[j,i]
   }
}

subint.edgelist<-as.tnet(data2)
subint2<-network(subint.edgelist[,1:2])
network::set.edge.attribute(subint2,"weight",as.vector(subint.edgelist[,3]))

network::set.vertex.attribute(subint2,"rank",as.vector(attributes$Rank))
network::set.vertex.attribute(subint2,"ranksq",as.vector(ranksquared))
network::set.vertex.attribute(subint2,"rankce",as.vector(centrank))
network::set.vertex.attribute(subint2,"sex",as.numeric(attributes$Sex))
network::set.vertex.attribute(subint2,"age",as.numeric(attributes$AgeClass))
network::set.vertex.attribute(subint2,"age2",as.numeric(as.numeric(attributes$AgeClass)>1)+1)
network::set.vertex.attribute(subint2,"rankcecat",as.vector(centrankcat))
network::set.vertex.attribute(subint2,"rankcecat2",as.vector(centrankcat2))


USF<-ergm(subint2~nonzero+sum+nodecov("rank")+nodecov("rankce")+absdiff("rank"),reference=~Geometric,respons
e="weight")
UDF<-ergm(domint2~nonzero+sum+nodecov("rank")+nodecov("rankce")+absdiff("rank"),reference=~Geometric,respons
e="weight")
UAF<-ergm(agint2~nonzero+sum+nodecov("rank")+nodecov("rankce")+absdiff("rank"),reference=~Geometric,response
="weight")

mcmc.diagnostics(USF)
```

```
## Sample statistics summary:
##
## Iterations = 16384:4209664
## Thinning interval = 1024
## Number of chains = 1
## Sample size per chain = 4096
##
## 1. Empirical mean and standard deviation for each variable,
##    plus standard error of the mean:
##
##                             Mean      SD Naive SE Time-series SE
## nonzero                   0.1113   10.96   0.1712         0.3111
## sum                       8.2419  209.08   3.2669         8.4185
## nodecov.sum..rank       116.8516 4531.14  70.7991       174.3632
## nodecov.sum..rankce     155.1709 3302.45  51.6007       135.6299
## absdiff.sum.rank       -120.7471 2098.27  32.7854        84.7477
##
## 2. Quantiles for each variable:
##
##                             2.5%     25%     50%    75%  97.5%
```

```
##                         2.5%   25%    50%  75% 97.5%
## nonzero               -22.0    -7    0.0    8    21
## sum                  -383.2  -140    7.0  146   422
## nodecov.sum..rank   -8376.0 -2952   23.0 3120  9574
## nodecov.sum..rankce -5945.2 -2134  120.5 2372  6754
## absdiff.sum.rank    -4028.4 -1599 -169.0 1228  4127
##
##
## Sample statistics cross-correlations:
##                       nonzero       sum nodecov.sum..rank
## nonzero             1.0000000 0.3785178         0.4714970
## sum                 0.3785178 1.0000000         0.8875285
## nodecov.sum..rank   0.4714970 0.8875285         1.0000000
## nodecov.sum..rankce 0.3233814 0.9402526         0.7659319
## absdiff.sum.rank    0.3643950 0.8145304         0.8319294
##                     nodecov.sum..rankce absdiff.sum.rank
## nonzero                       0.3233814        0.3643950
## sum                           0.9402526        0.8145304
## nodecov.sum..rank             0.7659319        0.8319294
## nodecov.sum..rankce           1.0000000        0.7961239
## absdiff.sum.rank              0.7961239        1.0000000
##
## Sample statistics auto-correlation:
## Chain 1
##             nonzero       sum nodecov.sum..rank nodecov.sum..rankce
## Lag 0    1.00000000 1.0000000         1.0000000           1.0000000
## Lag 1024 0.45527737 0.7170914         0.7022226           0.7237296
## Lag 2048 0.24318810 0.5359331         0.5080038           0.5479163
## Lag 3072 0.17359884 0.4003933         0.3645139           0.4171664
## Lag 4096 0.10453095 0.2975852         0.2567240           0.3183813
## Lag 5120 0.07206562 0.2302437         0.1802457           0.2482641
##          absdiff.sum.rank
## Lag 0           1.0000000
## Lag 1024        0.7147027
## Lag 2048        0.5365661
## Lag 3072        0.4020915
## Lag 4096        0.2968280
## Lag 5120        0.2277672
##
## Sample statistics burn-in diagnostic (Geweke):
## Chain 1
##
## Fraction in 1st window = 0.1
## Fraction in 2nd window = 0.5
##
##             nonzero                 sum   nodecov.sum..rank
##              0.1681              0.7655              1.2446
## nodecov.sum..rankce    absdiff.sum.rank
##              0.3348              1.1958
##
## Individual P-values (lower = worse):
##             nonzero                 sum   nodecov.sum..rank
##           0.8665004           0.4439698           0.2132605
## nodecov.sum..rankce    absdiff.sum.rank
##           0.7377768           0.2317574
## Joint P-value (lower = worse):  0.742898 .
```

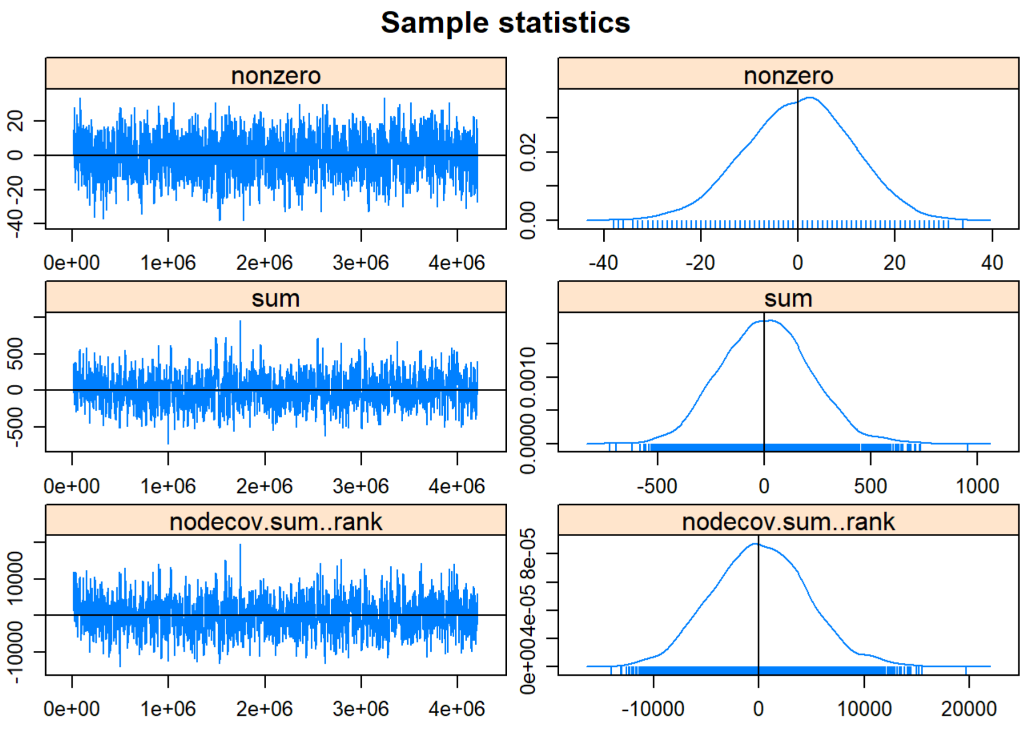

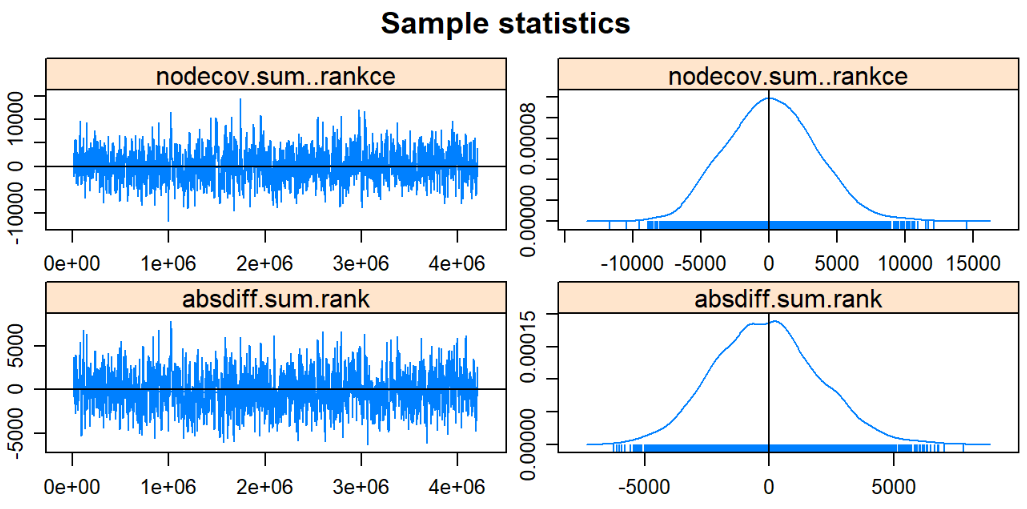


```
##
## MCMC diagnostics shown here are from the last round of simulation, prior to computation of final paramete
r estimates. Because the final estimates are refinements of those used for this simulation run, these diagno
stics may understate model performance. To directly assess the performance of the final model on in-model st
atistics, please use the GOF command: gof(ergmFitObject, GOF=~model).
```

```
mcmc.diagnostics(UDF)
```

```
## Sample statistics summary:
##
## Iterations = 16384:4209664
## Thinning interval = 1024
## Number of chains = 1
## Sample size per chain = 4096
##
## 1. Empirical mean and standard deviation for each variable,
##    plus standard error of the mean:
```

```
##
##                         Mean       SD Naive SE Time-series SE
## nonzero              -0.3372    8.269   0.1292         0.1575
## sum                   3.9092   76.872   1.2011         1.9233
## nodecov.sum..rank    36.0137 1574.811  24.6064        35.9933
## nodecov.sum..rankce  75.9053 1253.734  19.5896        32.4552
## absdiff.sum.rank      1.3735  707.559  11.0556        16.7598
##
## 2. Quantiles for each variable:
##
##                       2.5%     25% 50%    75%  97.5%
## nonzero                -17    -6.0   0    5.0   16.0
## sum                   -142   -50.0   2   56.0  160.6
## nodecov.sum..rank    -2956 -1027.2   6 1117.2 3142.0
## nodecov.sum..rankce -2246  -778.2  26  898.8 2636.2
## absdiff.sum.rank    -1378  -488.0 -19  477.0 1408.5
##
##
## Sample statistics cross-correlations:
##                       nonzero       sum nodecov.sum..rank
## nonzero             1.0000000 0.3962395         0.5227887
## sum                 0.3962395 1.0000000         0.8669928
## nodecov.sum..rank   0.5227887 0.8669928         1.0000000
## nodecov.sum..rankce 0.3259736 0.9352281         0.7062986
## absdiff.sum.rank    0.4036926 0.7942816         0.8167455
##                     nodecov.sum..rankce absdiff.sum.rank
## nonzero                       0.3259736        0.4036926
## sum                           0.9352281        0.7942816
## nodecov.sum..rank             0.7062986        0.8167455
## nodecov.sum..rankce           1.0000000        0.7412875
## absdiff.sum.rank              0.7412875        1.0000000
##
## Sample statistics auto-correlation:
## Chain 1
##             nonzero        sum nodecov.sum..rank nodecov.sum..rankce
## Lag 0    1.00000000 1.00000000        1.00000000          1.00000000
## Lag 1024 0.13505993 0.42053010        0.36288327          0.44695038
## Lag 2048 0.02079872 0.19511163        0.14675701          0.21875444
## Lag 3072 0.01798538 0.09501220        0.05665587          0.12295613
## Lag 4096 0.04749802 0.05521972        0.03440553          0.07878288
## Lag 5120 0.03127224 0.01516754        0.01288955          0.03771716
##          absdiff.sum.rank
## Lag 0          1.00000000
## Lag 1024       0.39348845
## Lag 2048       0.15427930
## Lag 3072       0.06070734
## Lag 4096       0.02740657
## Lag 5120       0.01408811
##
## Sample statistics burn-in diagnostic (Geweke):
## Chain 1
##
## Fraction in 1st window = 0.1
## Fraction in 2nd window = 0.5
##
##             nonzero                 sum   nodecov.sum..rank
##            -1.28206             0.15270             0.08338
## nodecov.sum..rankce    absdiff.sum.rank
##             0.11828            -0.33494
##
## Individual P-values (lower = worse):
##             nonzero                 sum   nodecov.sum..rank
##           0.1998219           0.8786327           0.9335521
## nodecov.sum..rankce    absdiff.sum.rank
##           0.9058472           0.7376693
## Joint P-value (lower = worse):  0.6255043 .
```

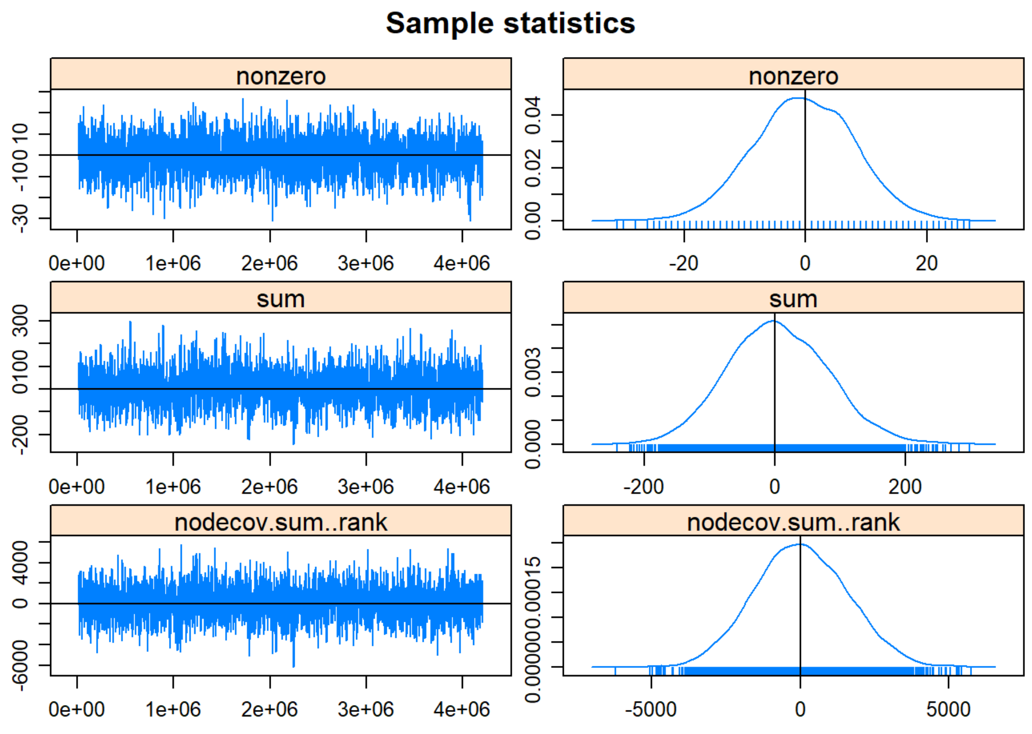

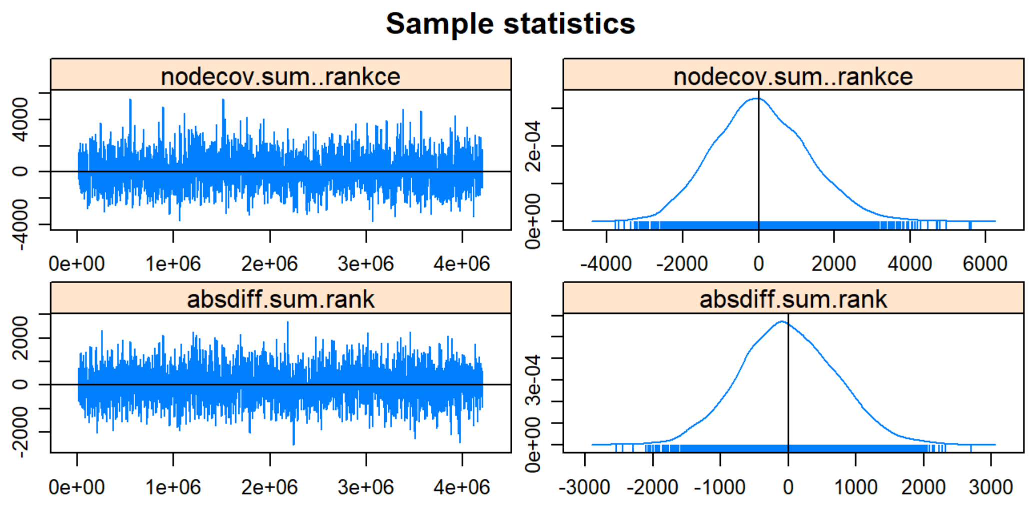


```
##
## MCMC diagnostics shown here are from the last round of simulation, prior to computation of final paramete
r estimates. Because the final estimates are refinements of those used for this simulation run, these diagno
stics may understate model performance. To directly assess the performance of the final model on in-model st
atistics, please use the GOF command: gof(ergmFitObject, GOF=~model).
```

```
mcmc.diagnostics(UAF)
```

```
## Sample statistics summary:
##
## Iterations = 16384:4209664
## Thinning interval = 1024
## Number of chains = 1
## Sample size per chain = 4096
##
## 1. Empirical mean and standard deviation for each variable,
##    plus standard error of the mean:
```

```
##
##                          Mean      SD Naive SE Time-series SE
## nonzero               0.1292    8.92   0.1394         0.1879
## sum                  13.2041  101.24   1.5818         2.9109
## nodecov.sum..rank   230.2388 1910.49  29.8514        51.3515
## nodecov.sum..rankce 180.5269 1628.66  25.4478        48.2196
## absdiff.sum.rank     61.1445  694.43  10.8504        18.0251
##
## 2. Quantiles for each variable:
##
##                      2.5%      25%   50%    75% 97.5%
## nonzero               -17    -6.00   0.0    6.0    17
## sum                  -174   -59.25  10.0   80.0   218
## nodecov.sum..rank   -3324 -1113.50 170.0 1518.8  4059
## nodecov.sum..rankce -2753  -978.00  89.5 1249.2  3521
## absdiff.sum.rank    -1215  -425.25  50.0  524.2  1497
##
##
## Sample statistics cross-correlations:
##                       nonzero       sum nodecov.sum..rank
## nonzero             1.0000000 0.4374803         0.5629635
## sum                 0.4374803 1.0000000         0.8580190
## nodecov.sum..rank   0.5629635 0.8580190         1.0000000
## nodecov.sum..rankce 0.3518129 0.9220474         0.6492163
## absdiff.sum.rank    0.5063839 0.7646199         0.7882914
##                     nodecov.sum..rankce absdiff.sum.rank
## nonzero                       0.3518129        0.5063839
## sum                           0.9220474        0.7646199
## nodecov.sum..rank             0.6492163        0.7882914
## nodecov.sum..rankce           1.0000000        0.6788059
## absdiff.sum.rank              0.6788059        1.0000000
##
## Sample statistics auto-correlation:
## Chain 1
##               nonzero        sum nodecov.sum..rank nodecov.sum..rankce
## Lag 0    1.000000000 1.00000000        1.00000000          1.00000000
## Lag 1024 0.289870202 0.52589135        0.46551548          0.54580938
## Lag 2048 0.096264950 0.29477594        0.24636715          0.31650770
## Lag 3072 0.045532914 0.16159554        0.12136999          0.18633489
## Lag 4096 0.007629155 0.08173069        0.05174461          0.11526504
## Lag 5120 0.021747931 0.05343132        0.02107705          0.09043547
##          absdiff.sum.rank
## Lag 0          1.00000000
## Lag 1024       0.44329658
## Lag 2048       0.22140583
## Lag 3072       0.11168733
## Lag 4096       0.05606521
## Lag 5120       0.03846662
##
## Sample statistics burn-in diagnostic (Geweke):
## Chain 1
##
## Fraction in 1st window = 0.1
## Fraction in 2nd window = 0.5
##
##             nonzero                 sum   nodecov.sum..rank
##             0.96646             0.73477             0.98026
## nodecov.sum..rankce    absdiff.sum.rank
##             0.07979             0.32392
##
## Individual P-values (lower = worse):
##             nonzero                 sum   nodecov.sum..rank
##           0.3338132           0.4624775           0.3269578
## nodecov.sum..rankce    absdiff.sum.rank
##           0.9364065           0.7459954
## Joint P-value (lower = worse):  0.1749243 .
```

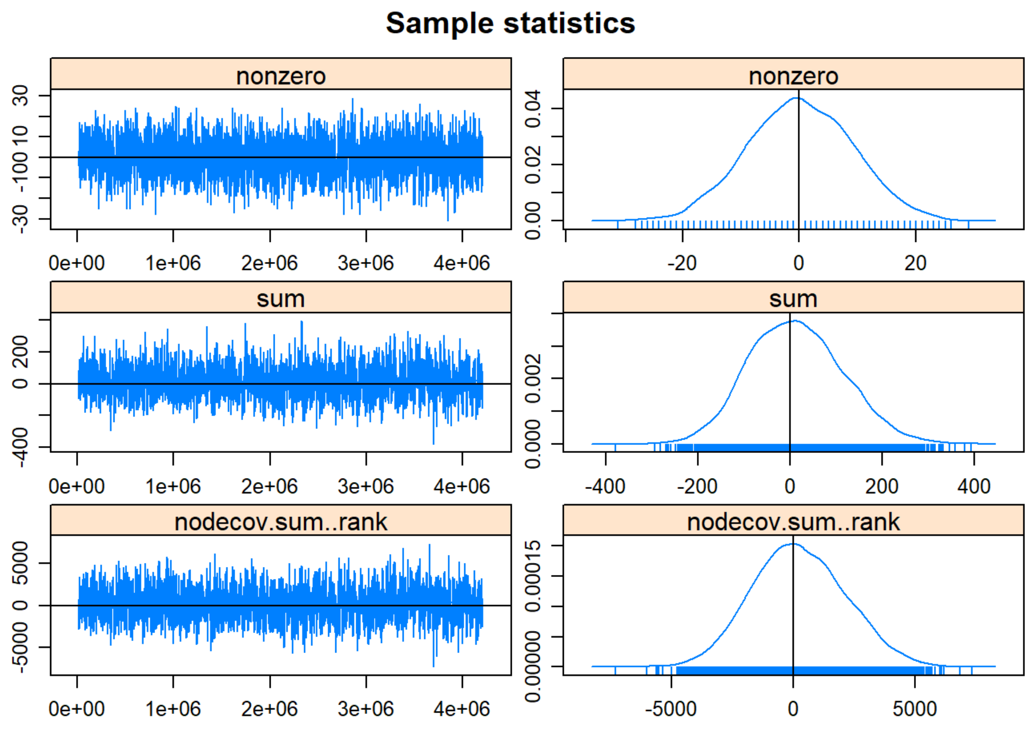

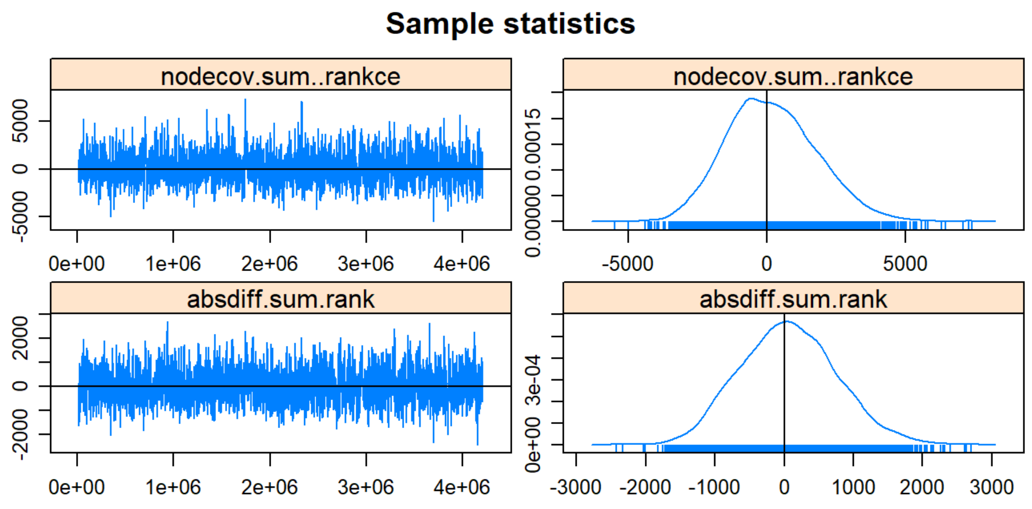


```
##
## MCMC diagnostics shown here are from the last round of simulation, prior to computation of final paramete
r estimates. Because the final estimates are refinements of those used for this simulation run, these diagno
stics may understate model performance. To directly assess the performance of the final model on in-model st
atistics, please use the GOF command: gof(ergmFitObject, GOF=~model).
```

```
summary(USF)
```

```
##
## ==========================
## Summary of model fit
## ==========================
##
## Formula:   subint2 ~ nonzero + sum + nodecov("rank") + nodecov("rankce") +
##     absdiff("rank")
##
## Iterations:  3 out of 20
##
## Monte Carlo MLE Results:
##                       Estimate Std. Error MCMC % p-value
## nonzero              -0.5153558  0.1042194      0  <1e-04 ***
## sum                   0.0045636  0.0224516      0   0.839
## nodecov.sum..rank   -0.0056419  0.0006577      0  <1e-04 ***
## nodecov.sum..rankce -0.0016238  0.0010833      0   0.134
## absdiff.sum.rank     0.0014614  0.0009853      0   0.138
## ---
## Signif. codes:  0 '***' 0.001 '**' 0.01 '*' 0.05 '.' 0.1 ' ' 1
##
##      Null Deviance:     0  on 702  degrees of freedom
##  Residual Deviance: -5524  on 697  degrees of freedom
##
## Note that the null model likelihood and deviance are defined to be 0.
##
## AIC: -5514    BIC: -5491    (Smaller is better.)
```

```
summary(UDF)
```

```
##
## ==========================
## Summary of model fit
## ==========================
##
## Formula:   domint2 ~ nonzero + sum + nodecov("rank") + nodecov("rankce") +
##     absdiff("rank")
##
## Iterations:  2 out of 20
##
## Monte Carlo MLE Results:
##                      Estimate Std. Error MCMC % p-value
## nonzero              -0.065997   0.144576      0   0.648
## sum                   0.073698   0.067034      0   0.272
## nodecov.sum..rank   -0.012627   0.001988      0  <1e-04 ***
## nodecov.sum..rankce -0.004583   0.002996      0   0.127
## absdiff.sum.rank     0.001103   0.002728      0   0.686
## ---
## Signif. codes:  0 '***' 0.001 '**' 0.01 '*' 0.05 '.' 0.1 ' ' 1
##
##      Null Deviance:     0  on 351  degrees of freedom
##  Residual Deviance: -2765  on 346  degrees of freedom
##
## Note that the null model likelihood and deviance are defined to be 0.
##
## AIC: -2755    BIC: -2735    (Smaller is better.)
```

```
summary(UAF)
```

```
##
## ==========================
## Summary of model fit
## ==========================
##
## Formula:   agint2 ~ nonzero + sum + nodecov("rank") + nodecov("rankce") +
##     absdiff("rank")
##
## Iterations:  3 out of 20
##
## Monte Carlo MLE Results:
##                      Estimate Std. Error MCMC % p-value
## nonzero             -0.993788   0.140707      0  <1e-04 ***
## sum                  0.123012   0.056169      0  0.0292 *
## nodecov.sum..rank   -0.009553   0.001759      0  <1e-04 ***
## nodecov.sum..rankce -0.005523   0.002403      0  0.0221 *
## absdiff.sum.rank    -0.006190   0.002554      0  0.0159 *
## ---
## Signif. codes:  0 '***' 0.001 '**' 0.01 '*' 0.05 '.' 0.1 ' ' 1
##
##      Null Deviance:     0  on 351  degrees of freedom
##  Residual Deviance: -3005  on 346  degrees of freedom
##
## Note that the null model likelihood and deviance are defined to be 0.
##
## AIC: -2995    BIC: -2976    (Smaller is better.)
```

This code will produce the plot of model estimates (Fig. 4)

It reproduces the plots shown in the paper and model estimates may change fractionally depending on the simulation step of the ERGMs

```
ys2<-c(-0.012547,-0.009605,-0.004622,-0.005642,0.001092,-0.006071)
sds2<-c(0.001987,0.001761,0.002889,0.002395,0.002784,0.002532)
xs2<-c(1,2,4,5,7,8)

cols<-rep(c("grey","gray32"),3)
par(xpd=FALSE)
plot(NULL,xlim=c(0,9),ylim=c(-0.02,0.01),xaxt="n",las=1,xlab="",ylab="",bty="n")
lines(x=c(-10,100),y=c(0,0))

points(y=ys2,x=xs2,cex=2,pch=18,col=cols)

for ( i in 1:6){
arrows(xs2[i],ys2[i]-1.96*sds2[i],xs2[i],ys2[i]+1.96*sds2[i],angle=90,code=3,lwd=2,col=cols[i])
}
lines(c(-5,20),c(0,0))

text(1,ys2[1]-1.96*sds2[1]-0.002,"***",cex=2)
text(2,ys2[2]-1.96*sds2[2]-0.002,"***",cex=2)
text(5,ys2[4]-1.96*sds2[6]-0.002,"*",cex=2)
text(8,ys2[6]-1.96*sds2[6]-0.002,"*",cex=2)

legend(x=0.75,y=0.012,legend=c("Ritualised Dominance interactions","Aggressive interactions"),fill=c("grey",
"gray32"),border=NA,bty="n",y.intersp=1.5,x.intersp=0.5)
```

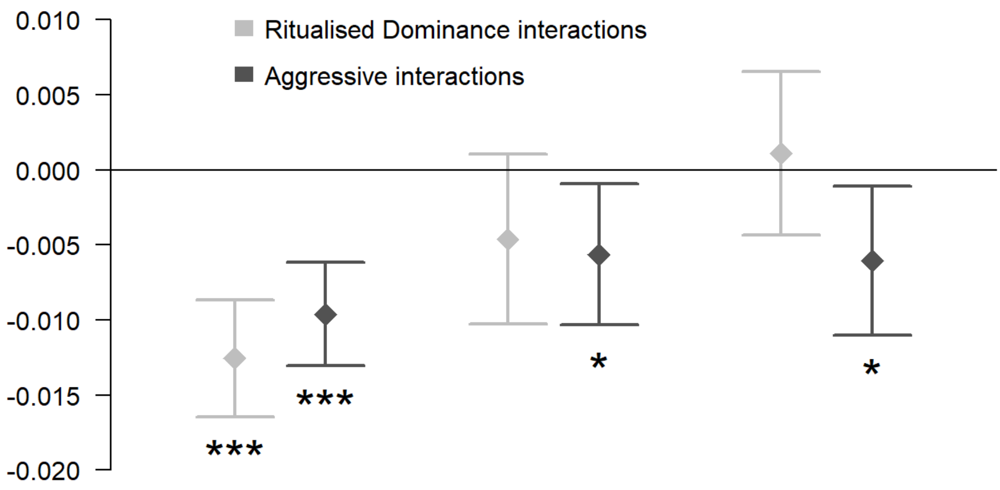

Code to produce network plots (both the hierarchy plot and undirected interactions plot)

This has been taken from a separate R script and can be run separately to the rest of the code

```
library(igraph)

data<-read.csv(paste0(direc,"DogDataagmat.csv"),header=T)
data<-as.matrix(data[,2:28])
rownames(data)<-colnames(data)

data2<-matrix(0,nr=27,nc=27)

for(i in 1:26){
   for(j in (i+1):27){
      data2[i,j]<-data[i,j]-data[j,i]
   }
}

for(i in 1:26){
   for(j in (i+1):27){
      if(data2[i,j]<0){
         data2[j,i]<-abs(data2[i,j])
         data2[i,j]<-0
      }
   }
}

data3<-matrix(0,nr=27,nc=27)

for(i in 1:26){
   for(j in (i+1):27){
      data3[i,j]<-data[i,j]+data[j,i]
      data3[j,i]<-data3[i,j]
   }
}

netA<-graph.adjacency(data,mode="directed",weighted=TRUE,diag=FALSE)

netA2<-graph.adjacency(data3,mode="undirected",weighted=TRUE,diag=FALSE)


atts<-read.csv(paste0(direc,"attributedat.csv"))
```

```r
mytriangle <- function(coords, v=NULL, params) {
  vertex.color <- params("vertex", "color")
  if (length(vertex.color) != 1 && !is.null(v)) {
    vertex.color <- vertex.color[v]
  }
  vertex.size <- 1/200 * params("vertex", "size")
  if (length(vertex.size) != 1 && !is.null(v)) {
    vertex.size <- vertex.size[v]
  }

  symbols(x=coords[,1], y=coords[,2], bg=vertex.color,
          stars=cbind(vertex.size, vertex.size, vertex.size),
          add=TRUE, inches=FALSE)
}
# clips as a circle
add.vertex.shape("triangle", clip=vertex.shapes("circle")$clip,
                 plot=mytriangle)

shapes<-rep(NA,27)
for (i in 1:27){
   if(atts[i,4]=="AD"){
      shapes[i]<-"square"
   }
   if(atts[i,4]=="SUB"){
      shapes[i]<-"circle"
   }
   if(atts[i,4]=="JUV"){
      shapes[i]<-"triangle"
   }
}

Mcols<-heat.colors(16,alpha=1)

blue.fade <- colorRampPalette(c("darkblue","royalblue3","turquoise3","honeydew"), space = "rgb")
red.fade <- colorRampPalette(c("red", "white"), space = "rgb")
#Mcols<-red.fade(16)
Fcols<-blue.fade(11)

cols<-rep(NA,27)
for (i in 1:27){
   if(atts[i,3]=="M"){
      cols[i]<-Mcols[1]
      Mcols<-Mcols[-1]
   }
   if(atts[i,3]=="F"){
      cols[i]<-Fcols[1]
      Fcols<-Fcols[-1]
   }
}

a<-plot(netA,edge.width=sqrt(E(netA)$weight),vertex.color=cols,vertex.shape=shapes,layout=cbind(seq(4,108,4)
,seq(108,4,-4)),vertex.size=6,vertex.label=NA,edge.curved=1,edge.arrow.size=0.8)
```

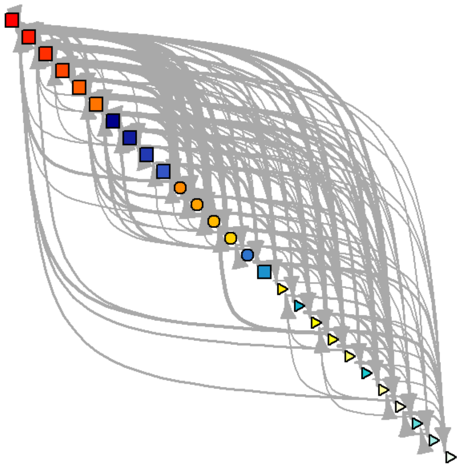

```
data<-read.csv(paste0(direc,"DogDatadommat.csv"),header=T)
data<-as.matrix(data[,2:28])
rownames(data)<-colnames(data)

data2<-matrix(0,nr=27,nc=27)

for(i in 1:26){
   for(j in (i+1):27){
      data2[i,j]<-data[i,j]-data[j,i]
   }
}

for(i in 1:26){
   for(j in (i+1):27){
      if(data2[i,j]<0){
         data2[j,i]<-abs(data2[i,j])
         data2[i,j]<-0
      }
   }
}

netB<-graph.adjacency(data,mode="directed",weighted=TRUE,diag=FALSE)

data3<-matrix(0,nr=27,nc=27)

for(i in 1:26){
   for(j in (i+1):27){
      data3[i,j]<-data[i,j]+data[j,i]
      data3[j,i]<-data3[i,j]
   }
}

netB2<-graph.adjacency(data3,mode="undirected",weighted=TRUE,diag=FALSE)

atts<-read.csv(paste0(direc,"attributedat.csv"))

mytriangle <- function(coords, v=NULL, params) {
  vertex.color <- params("vertex", "color")
  if (length(vertex.color) != 1 && !is.null(v)) {
    vertex.color <- vertex.color[v]
  }
  vertex.size <- 1/200 * params("vertex", "size")
  if (length(vertex.size) != 1 && !is.null(v)) {
    vertex.size <- vertex.size[v]
  }

  symbols(x=coords[,1], y=coords[,2], bg=vertex.color,
          stars=cbind(vertex.size, vertex.size, vertex.size),
          add=TRUE, inches=FALSE)
}
# clips as a circle
add.vertex.shape("triangle", clip=vertex.shapes("circle")$clip,
                 plot=mytriangle)

shapes<-rep(NA,27)
for (i in 1:27){
   if(atts[i,4]=="AD"){
      shapes[i]<-"square"
   }
   if(atts[i,4]=="SUB"){
      shapes[i]<-"circle"
   }
   if(atts[i,4]=="JUV"){
      shapes[i]<-"triangle"
   }
}

b<-plot(netB,edge.width=sqrt(E(netB)$weight),vertex.color=cols,vertex.shape=shapes,layout=cbind(seq(4,108,4)
,seq(108,4,-4)),vertex.size=6,vertex.label=NA,edge.curved=1,edge.arrow.size=0.8)
```

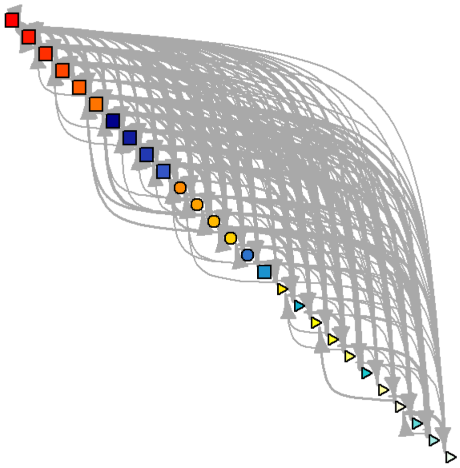

```r
#-------------------------------------

data<-read.csv(paste0(direc,"DogDatasubmat.csv"),header=T)
data<-as.matrix(data[,2:28])
rownames(data)<-colnames(data)

data2<-matrix(0,nr=27,nc=27)

for(i in 1:26){
   for(j in (i+1):27){
      data2[i,j]<-data[i,j]-data[j,i]
   }
}

for(i in 1:26){
   for(j in (i+1):27){
      if(data2[i,j]<0){
         data2[j,i]<-abs(data2[i,j])
         data2[i,j]<-0
      }
   }
}

netC<-graph.adjacency(data,mode="directed",weighted=TRUE,diag=FALSE)

data3<-matrix(0,nr=27,nc=27)

for(i in 1:26){
   for(j in (i+1):27){
      data3[i,j]<-data[i,j]+data[j,i]
      data3[j,i]<-data3[i,j]
   }
}

netC2<-graph.adjacency(data3,mode="undirected",weighted=TRUE,diag=FALSE)

atts<-read.csv(paste0(direc,"attributedat.csv"))

mytriangle <- function(coords, v=NULL, params) {
  vertex.color <- params("vertex", "color")
  if (length(vertex.color) != 1 && !is.null(v)) {
    vertex.color <- vertex.color[v]
  }
  vertex.size <- 1/200 * params("vertex", "size")
  if (length(vertex.size) != 1 && !is.null(v)) {
    vertex.size <- vertex.size[v]
  }

  symbols(x=coords[,1], y=coords[,2], bg=vertex.color,
          stars=cbind(vertex.size, vertex.size, vertex.size),
          add=TRUE, inches=FALSE)
}
# clips as a circle
add.vertex.shape("triangle", clip=vertex.shapes("circle")$clip,
                 plot=mytriangle)

shapes<-rep(NA,27)
for (i in 1:27){
   if(atts[i,4]=="AD"){
      shapes[i]<-"square"
   }
   if(atts[i,4]=="SUB"){
      shapes[i]<-"circle"
   }
   if(atts[i,4]=="JUV"){
      shapes[i]<-"triangle"
   }
}

c<-plot(netC,edge.width=sqrt(E(netC)$weight),vertex.color=cols,vertex.shape=shapes,layout=cbind(seq(4,108,4)
,seq(108,4,-4)),vertex.size=6,vertex.label=NA,edge.curved=1,edge.arrow.size=0.6)
```

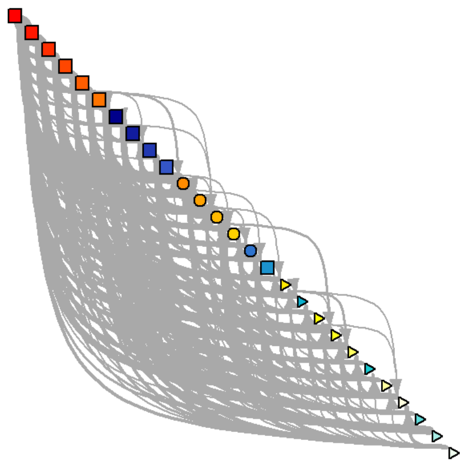

```
layout=layout.fruchterman.reingold(netC2)
#submission
pb<-plot(netC2,edge.width=sqrt(E(netC2)$weight/2),vertex.color=cols,vertex.shape=shapes,vertex.size=8,vertex
.label=NA,edge.curved=0.2,edge.arrow.size=0.5,layout=layout)
```

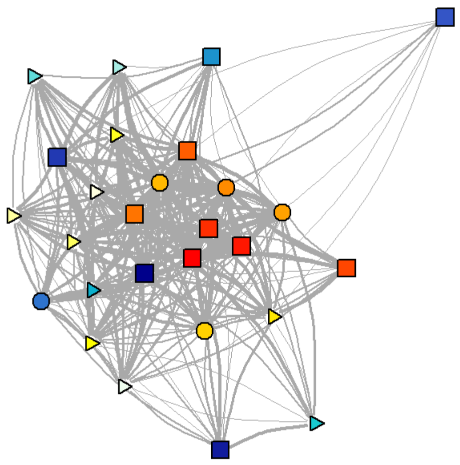

```
#dominance
pa<-plot(netB2,edge.width=sqrt(E(netB2)$weight/2),vertex.color=cols,vertex.shape=shapes,vertex.size=8,vertex
.label=NA,edge.curved=0.2,edge.arrow.size=0.5,layout=layout)
```

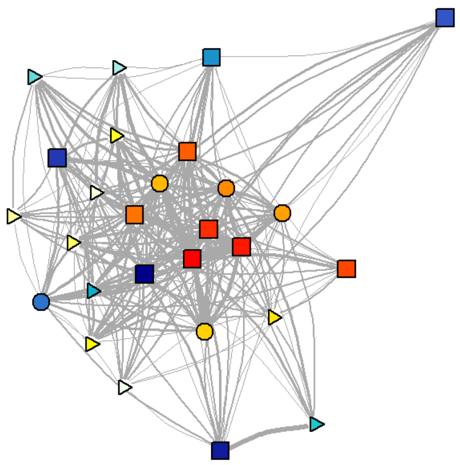

```
#aggression
pc<-plot(netA2,edge.width=sqrt(E(netA2)$weight/2),vertex.color=cols,vertex.shape=shapes,vertex.size=8,vertex
.label=NA,edge.curved=0.2,edge.arrow.size=0.5,layout=layout)
```

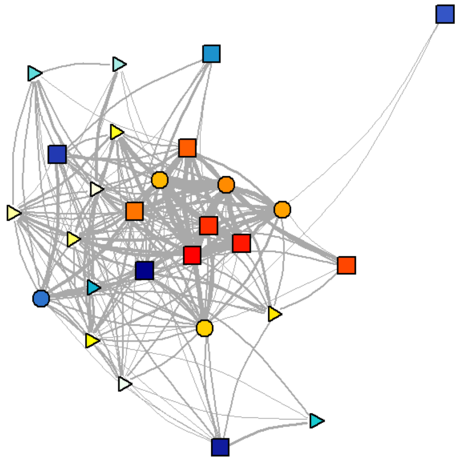

Supplement: Supplementary Methods and Code [file rspb20190536supp1.pdf]
